# Gliadin-specific CD4^+^ T cells exhibit functional diversity independent of TCR usage or epitope specificity

**DOI:** 10.1126/sciadv.aei3175

**Published:** 2026-10-02

**Authors:** Claerwen M. Jones, Jia Jia Lim, Stefan A. Schattgen, Tiing Jen Loh, Hien Thy Dao, Mai T. Tran, Angela Nguyen, Pirooz Zareie, Nathan J. Felix, Paul G. Thomas, Melinda Y. Hardy, Jason A. Tye-Din, Hugh H. Reid, Jamie Rossjohn, Nicole L. La Gruta

**Affiliations:** ^1^ Immunity Program and Department of Biochemistry and Molecular Biology, Biomedicine Discovery Institute, Monash University, Clayton, Victoria, Australia.; ^2^ Department of Host-Microbe Interactions, St Jude Children’s Research Hospital, Memphis, TN, USA.; ^3^ Department of Microbiology and Immunology, The University of Melbourne at the Peter Doherty Institute for Infection and Immunity, Melbourne, Victoria, Australia.; ^4^ Janssen Research and Development, Spring House, PA, USA.; ^5^ Immunology Division, The Walter and Eliza Hall Institute, Parkville, Victoria, Australia.; ^6^ Department of Medical Biology, University of Melbourne, Parkville, Victoria, Australia.; ^7^ Department of Gastroenterology, The Royal Melbourne Hospital, Parkville, Victoria, Australia.

## Abstract

Celiac disease (CeD) is driven by pathogenic CD4^+^ T cell responses, primarily targeting four HLA-DQ2.5–restricted immunodominant epitopes derived from α-gliadin and ω-gliadin. While gliadin-specific CD4^+^ T cells have been investigated, how transcriptional and effector phenotypes in CeD vary across distinct epitope specificities remains unclear, as do the signature TCR motifs governing gliadin epitope specificity and cross-reactivity. Single-cell transcriptomic and TCR profiling of gliadin epitope-specific peripheral blood CD4^+^ T cells from patients with CeD after gluten challenge revealed T cells with an activated T_H_1 effector, conventional regulatory T cell (T_reg_ cell) or cytotoxic phenotypes across specificities, with no evidence of functional specialization driven by specific epitopes. Moreover, we identified core TCR motifs that dictate gliadin epitope specificity and cross-reactivity and elucidated the structural basis of their cognate pMHC recognition. These motifs enabled accurate prediction of gliadin specificity from unassigned TCRs. Collectively, these findings advance our understanding of immune responses in CeD and provide a foundation for tracking disease-relevant T cells.

## INTRODUCTION

Celiac disease (CeD) is a chronic, immune-mediated small intestinal enteropathy triggered in genetically susceptible individuals by the ingestion of cereal gluten proteins (*1*). The disease, which affects ∼1% of the population (*2*), is largely restricted to individuals expressing HLA-DQ2.5, HLA-DQ2.2, or HLA-DQ8, with ∼90% of patients with CeD expressing HLA-DQ2.5 (*3*). These HLA-DQ molecules present deamidated gluten peptides to CD4^+^ T cells (*4*), and there is compelling evidence that the gluten-specific CD4^+^ T cells drive the immune processes that lead to CeD pathogenesis, including intraepithelial cytotoxic T cell–mediated enterocyte destruction (*5*). Further, these cells are selectively present in the gut and blood of patients with CeD but not in healthy individuals (*6*–*8*), and gluten-specific CD4^+^ T cell clonotypes can persist in both circulation and intestinal tissue for decades even on a gluten-free diet (*9*). In HLA-DQ2.5–associated CeD, while more than 20 gluten T cell epitopes have been identified (*10*), approximately half of the gluten-reactive CD4^+^ T cell clones in most patients respond to four conserved immunodominant epitopes DQ2.5–glia-α1a (PFPQPELPY), glia-α2 (PQPELPYPQ), glia-ω1 (PFPQPEQPF), and glia-ω2 (PQPEQPFPW) (*11*, *12*). Because of the homologous nature of the epitopes, glia-α1a/ω1 and glia-α2/ω2 cross-reactive CD4^+^ T clones have also been identified (*11*–*14*). Moreover, we have described the molecular basis of T cell receptor (TCR) cross-reactivity between bacterial mimics of gliadin epitopes that may be related to CeD pathogenesis (*15*). CeD represents a unique paradigm in human T cell–mediated autoimmunity, given that it is the only common autoimmune disease in which the initiating antigens and disease-driving CD4^+^ T cell epitopes are precisely defined.

TCR signatures have the potential to serve as T cell–based diagnostics (*16*, *17*), and TCR repertoires of CD4^+^ T cells specific for individual immunodominant gluten epitopes are indeed characterized by biased V gene usage and some use of identical TCR alpha and/or beta chain sequences across multiple patients with CeD (*9*, *12*–*14*, *18*–*20*). However, while reproducible (public) TCR CDR3 sequences have been described, only one to date has been shown to define the molecular basis for DQ2.5-restricted immunodominant epitope recognition—a conserved nongermline encoded arginine within the CDR3β region of the dominant *TRAV26-1/TRBV7-2* CD4^+^ T cell clonal response to DQ2.5–glia-α2 (*20*). The critical residues or core CDR3 motifs defining antigen specificity and cross-reactivity to other DQ2.5-restricted immunodominant gliadin epitopes in CeD have not yet been described.

Understanding the molecular and functional properties of these pathogenic T cells is essential for developing targeted therapies aimed at inducing tolerance or selectively depleting these populations. Recent RNA sequencing (RNA-seq) studies of gluten-specific CD4^+^ T cells isolated from the gut and/or peripheral blood of untreated patients with CeD or treated patients on a short-term oral gluten challenge using pooled tetramers to immunodominant epitopes have shown that these cells have a distinct activated effector memory phenotype consistent with their disease-driving potential (*21*–*23*). To date, however, single-cell RNA-seq (scRNA-seq) studies of CD4^+^ T cells with known antigen specificity in CeD are lacking (*24*), leaving a critical gap in understanding whether individual disease-relevant epitopes imprint distinct transcriptional programs at the level of single T cells across patients.

In this study, we have used DNA-barcoded major histocompatibility complex (MHC) dextramers and antibodies combined with single-cell RNA and TCR sequencing to comprehensively map the transcriptional profile and paired αβTCR usage of CD4^+^ T cells specific for each of the four immunodominant DQ2.5 gliadin epitopes in CeD donor peripheral blood mononuclear cells (PBMCs) mobilized from the gut after oral wheat challenge. We show that the previously described activated effector memory phenotype is highly consistent both within and across the four distinct gliadin epitope specificities. Further, populations of regulatory T cells and cytotoxic CD4 T cells were also observed across specificities; however, there was no evidence that distinct gliadin epitope specificities drove functional specialization among CD4^+^ T cells, in contrast to other instances in human immune responses where epitope specificity biases lineage differentiation (*25*–*29*). TCR repertoire analysis highlighted core CDR3 motifs—enriched within epitope-specific populations independently of TRAV/J and TRBV/J biases, and across multiple CeD donors—associated with conferring epitope specificity and cross-reactivity. Moreover, we provide the molecular basis of a core CDR3 motif that enabled cross-reactivity to DQ2.5–glia-α1a and DQ2.5–glia-ω1 epitopes and specificity toward the DQ2.5–glia-ω2 determinant. Further, we show that the core CDR3 motifs were predictive of epitope specificity and cross-reactivity in a large unassigned TCR database of gluten-specific CD4^+^ T cells, indicating their potential as diagnostic indicators of CeD.

## RESULTS

### Multiomic single-cell profiling of CD4^+^ T cell responses to HLA-DQ2.5-gliadin epitopes in celiac disease

To comprehensively map the gluten epitope-specific CD4^+^ T cell response in patients with CeD and interrogate associated transcriptional profiles, we performed multiomic single-cell profiling of CD4^+^ T cells specific for four immunodominant wheat epitopes (HLA-DQ2.5–gliadin-α1a, HLA-DQ2.5–gliadin-α2, HLA-DQ2.5–gliadin-ω1, and HLA-DQ2.5–gliadin-ω2) after gluten challenge. CD14^−^CD19^−^CD3^+^CD4^+^dextramer^+^ cells were sorted from four CeD donor PBMCs collected 6 days after gluten challenge (table S1 and figs. S1, A and B). The pooled cells were subjected to single-cell Chromium 10x immune profiling with TotalSeq-C antibody barcoding and VDJ (TCR sequencing) analysis to simultaneously characterize gene expression, paired TCRαβ sequences, epitope specificity, and donor identity from individual cells from each of the four donors. After quality control (QC) filtering to remove low-quality cells and those with below-threshold dextramer binding, transcriptional and paired TCRαβ information was obtained from a total of 3317 cells across the four patients (Fig. 1A and fig. S1C), with most cells contributed by donor 1621 [hashtag oligo 4 (HTO4)] (fig. S1B). We identified cells specific for each of the unique epitopes DQ2.5–glia-α1a, DQ2.5–glia-α2, DQ2.5–glia-ω1, and DQ2.5–glia-ω2 as well as cells that were cross-reactive across the highly homologous DQ2.5–glia-α1a (PFPQPELPY) and DQ2.5–glia-ω1 (PFPQPEQPF) epitopes within each donor (Fig. 1B). Although TCR cross-reactivity between DQ2.5–glia-α2 (PQPELPYPQ) and DQ2.5–glia-ω2 (PQPEQPFPW) epitopes has been reported (*30*), the frequency of these T cells in our dataset was rare, with only a single clone (with two cells) from one donor identified and subsequently confirmed with this reactivity. The frequency of epitope-specific and cross-reactive T cells to the four DQ2.5-gliadin epitopes varied between each donor and corresponded to previously described frequencies (*14*, *31*–*34*). Although the cohort size precludes statistical assessment, epitope-specific T cell frequencies did not show obvious variation with HLA-DQ2.5 zygosity (Fig. 1B), consistent with prior reports (*31*). Expanded T cell clones were observed to all gliadin epitopes in each of the four patients with CeD, with ∼50% of clonotypes from each patient showing expansions after wheat challenge (fig. S1D). Thus, these profiles were characteristic of CD4^+^ T cells responding to all four gliadin-derived epitopes.

**Fig. 1.**
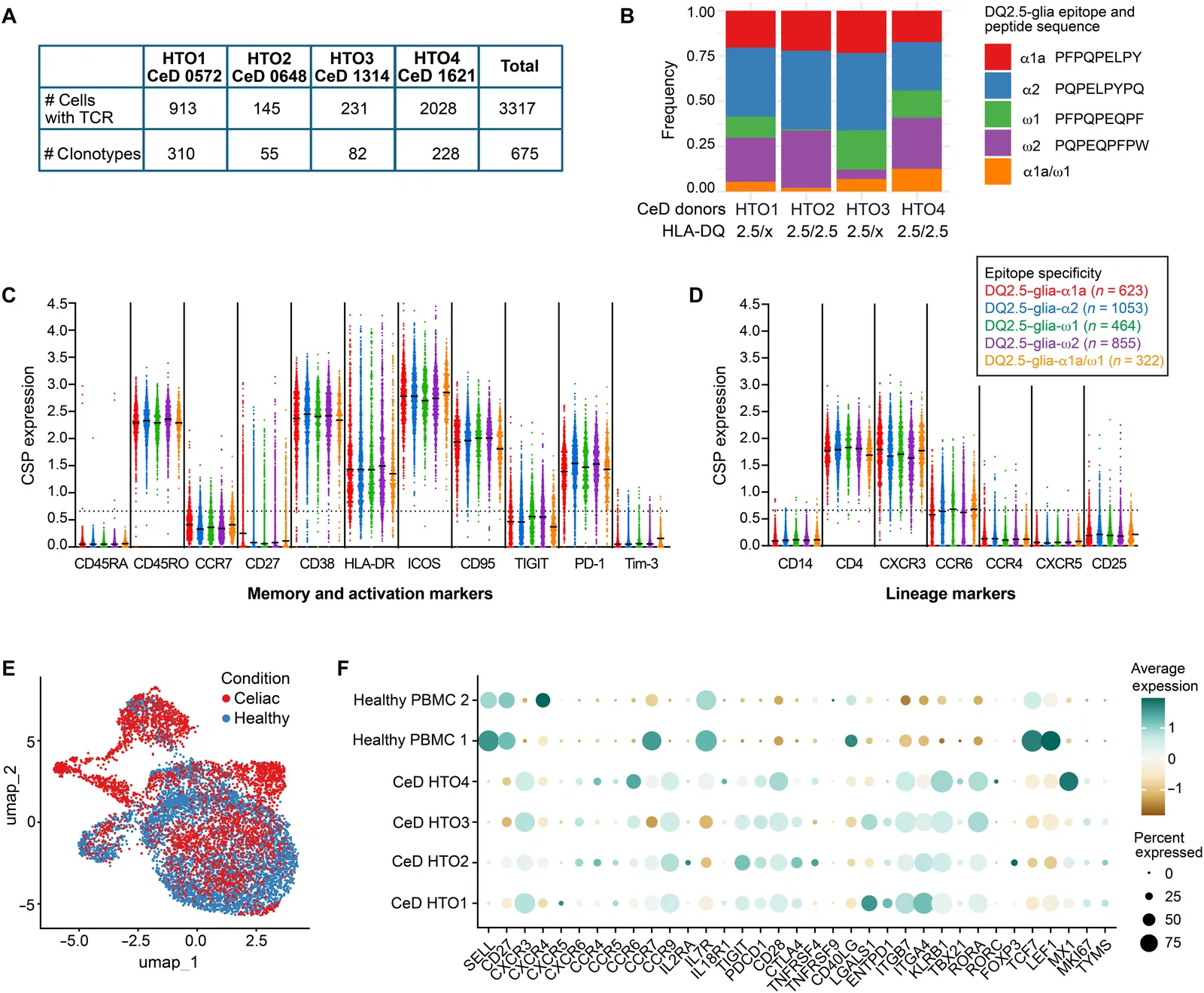
Gluten-specific CD4^+^ T cells express an activated T_H_1 phenotype. (**A**) Cells for which transcriptional, TCR sequence and TCR clonotype information was obtained for each patient (HTO1 to HTO4). (**B**) Distribution of epitope-specific cells within each donor (HTO1 to HTO4) as determined by DQ2.5–glia-α1a, DQ2.5–glia-α2, DQ2.5–glia-ω1, and DQ2.5–glia-ω2 dextramer binding. HLA-DQ2.5 zygosity is indicated where *x* denotes a haplotype other than HLA-DQ2.5. CSP expression of (**C**) memory and activation markers and (**D**) cell lineage markers as measured by TotalSeq-C antibody barcoding on DQ2.5–glia-α1a, DQ2.5–glia-α2, DQ2.5–glia-ω1, DQ2.5–glia-ω2, or DQ2.5–glia-α1a/ω1 dextramer-binding CD4^+^ T cells. Horizontal lines indicate median values. Dotted line indicates a nominal cutoff for positive expression based on known negative markers (CD45RA and CD14) and known positive markers (CD45RO and CD4). (**E**) Uniform Manifold Approximation and Projection (UMAP) of single cells from merged datasets containing DQ2.5-gliadin epitope-specific CD4^+^ T cells from the four celiac donors (red) and nonantigen-specific CD4^+^ T cells from two healthy control donors (blue). (**F**) Bubble plot showing the expression of representative differentially expressed genes in the two healthy control and four CeD patient donors. Circle size shows the proportion of cells expressing the gene, and color shows gene expression level.

### Gluten epitope-specific CD4^+^ T cells exhibit an activated, effector memory T_H_1 phenotype

The analysis of activation state and T helper (T_H_) lineage was initially determined via cell surface phenotype as detected by TotalSeq-C antibody tags. CD4^+^ T cells across all epitope specificities showed remarkable reproducibility in their cell surface phenotype (Fig. 1, C and D). They broadly exhibited an effector memory phenotype (CD45RA^−^ and CCR7^−^) with high expression of CD38 and other markers of activation (ICOS, CD95, and PD-1) (*35*–*37*) and low expression of Tim-3 (*38*) (Fig. 1C). Most cells expressed the chemokine receptor CXCR3 indicating a T_H_1 phenotype (Fig. 1D). Approximately half of these CXCR3^+^ cells also expressed the gut-homing marker CCR6 (Fig. 1D) (*39*), a subset that has been shown to be enriched for interferon-γ (IFN-γ)–producing T_H_1 cells with some cells additionally producing interleukin-17 (IL-17) (*40*). The memory, activation, and lineage marker expression were in concordance with that previously described for pooled gluten-specific CD4^+^ T cells in the blood of patients with CeD following wheat challenge (*22*) and was also virtually identical to that of pooled gluten-specific CD4^+^ T cells in the gut and blood of untreated patients with CeD (*21*). We show that this activated T_H_1 effector phenotype is highly stable across the four distinct specificities (Fig. 1, C and D).

The activated effector memory T_H_1 phenotypic signature was further confirmed by analyzing the transcriptional profile of the dextramer-binding CD4^+^ cells in patients with CeD compared with CD4^+^ T cell publicly available scRNA-seq datasets for two healthy control subjects, of which healthy PBMC 2 was HLA-DQ2.5 heterozygous. The CeD patient scRNA-seq data were integrated with that of the healthy subjects using the Harmony algorithm, treating each sample as a batch variable, thereby correcting for chemistry- and processing-associated effects while preserving biological variation. Integration showed that gliadin-specific CD4^+^ T cells from the four patients with CeD had a largely distinct gene expression profile to total CD4^+^ cells from healthy donor PBMCs (Fig. 1E). In contrast to CD4^+^ T cells from both DQ2.5-negative and DQ2.5-positive healthy donors, gliadin-specific CD4^+^ T cells from all four DQ2.5^+^ patients with CeD displayed a uniform down-regulation of *SELL* (CD62L), *CD27*, and *IL7R* and up-regulation of *CD28*, *CTLA4*, *KLRB1* (CD161), and *CCR5*, characteristic of the induced effector phenotype (Fig. 1F). We also observed the up-regulation of *PDCD1* (PD-1) and *TIGIT*,suggestive of chronic activation (*21*, *41*). The up-regulation of *CCR9*, *CXCR6*, *ITGB7*, and *ITGA4* was indicative of gluten-specific cells homing to the gut (*22*, *23*) (Fig. 1F). Last, we observed uniform up-regulation of T_H_1 markers *CXCR3* and *TBX21* (Tbet) and some up-regulation of T_H_17 markers *CCR6* and *RORC* (Fig. 1F). Thus, gluten epitope-specific CD4^+^ T cells in each of the CeD donors exhibited an activated effector memory T_H_1 phenotype.

### Transcriptional profile differs by patient and disease severity, but not epitope specificity

To determine whether distinct gene expression profiles were associated with epitope specificity, we performed unsupervised clustering on the dextramer-binding CD4^+^ T cells from the patients with CeD. Eight clusters were identified, composed of cell numbers ranging from 1061 cells in cluster 0 to 25 cells in cluster 7 (Fig. 2A). Cells across most clusters shared similarly the up-regulated expression of genes related to the mobilized, activated effector memory T_H_1 phenotypic signature (Fig. 2B). However, differences in transcriptional profiles were observed, relating to cellular differentiation and metabolic function, which were most strongly aligned to donor source (Fig. 2C). In particular, cells from donor HTO4, which dominated clusters 1 and 2, expressed a more inflammatory and differentiated profile, with cells in cluster 1 particularly up-regulated for genes associated with interferon stimulations (ISGs; e.g., *MX1*, *OAS1*, *ISG15*, *IFIT1*, and *IFI6*) (Fig. 2B and fig. S2A) (*42*), while cells in cluster 2 expressed a relatively decreased differentiation/activation state with relatively higher transcript levels for stemness genes *TCF7*, *LEF1*, and *CCR7* and lower expression of ISGs (Fig. 2B and fig. S2A). The more inflammatory profile observed in cells from donor HTO4 coincided with higher-level production of IFN-γ by CD4^+^ T cells in response to stimulation with gliadin peptides in vitro and more severe patient symptoms post wheat challenge compared to donors HTO1-3 (tables S1 and S2). While far from definitive, this observation suggests a potential link between inflammatory T cell states and disease severity that warrants further investigation in larger, appropriately powered cohorts. Further, it indicates that most of the transcriptional and, by inference, functional variation is associated with patient-specific factors.

**Fig. 2.**
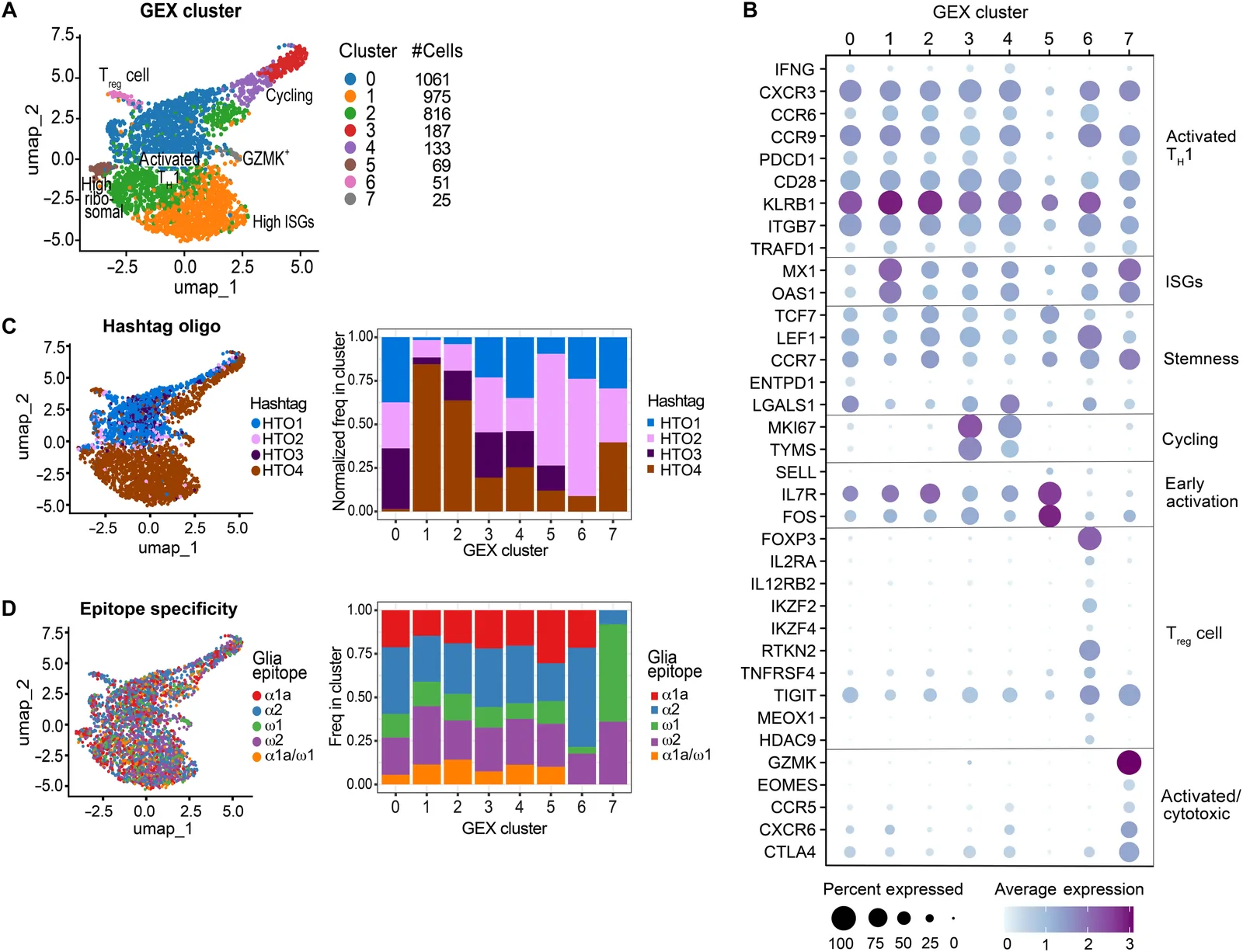
Transcriptional profiling unveils interferon-stimulated genes, cytotoxic CD4^+^ T cells and regulatory T cells. (**A**) UMAP representation of all gluten-specific CD4^+^ T cells based on gene expression (GEX). Graph-based unsupervised clustering performed with the Seurat package. (**B**) Bubble plot showing the expression of selected genes defining specific cluster identities. Circle size shows the proportion of cells expressing the gene, and color shows the gene expression level (scale 0 to 3). (**C** and **D**) UMAP plots showing the association of GEX clustering to (C) donor identity as determined by hashtag oligo (HTO) labeling and (D) epitope specificity. The distribution of cells within each GEX cluster based on donor identity and epitope specificity is also shown, with distribution in (C) normalized for the different number of cells sourced from each donor.

Cells in cluster 0, predominantly sourced from donors HTO1-3 and largely absent from HTO4 (Fig. 2C), appeared to be more typical T_H_1 cells, with lower expression of ISG transcripts and higher expression of mitochondrial genes involved in oxidative phosphorylation (*MT-CO2*, *MT-CYB*, and *MT-CO3*), associated with activation and proliferation of memory T cells (fig. S2A) (*43*, *44*), as well as higher expression of negative regulators CD39 (*ENTPD1*) and *LGALS* (Fig. 2B). A more even distribution of cells from the four patients with CeD was observed in clusters 3 and 4 (Fig. 2C), which were enriched for genes related to cell cycling (e.g., *MKI67*, *TYMS*, *PLK1*, and *CDC20*; Fig. 2B and fig. S2A) (*45*–*47*).

Intriguingly, GEX clusters were found to be largely independent of epitope specificity, with relatively even distributions of the various epitope specificities across most clusters (Fig. 2D). One exception was cluster 7, which was dominated by cells from an expanded DQ2.5–glia-ω1–specific clone from HTO4 (56% of cells). This small cluster, extending from cluster 0, was found to have up-regulated expression of genes associated with activated T cells (e.g., *CCR5*, *CXCR6*, and *CTLA4*; Fig. 2B) as well as up-regulated expression of *GZMK*, *GZMA*, *CCL4*, *PLEK*, and *NKG7* and the transcription factor *EOMES* (Fig. 2B and fig. S2, A to C), indicative of a cytotoxic signature in CD4^+^ T cells (*48*–*50*). The up-regulation of long intergenic noncoding RNAs (lincRNAs), including *IFNG-AS1* (*TMEVPG1*) that drives IFN-γ expression in T_H_1 cells (*51*), was also observed in this cluster (fig. S2, A and B). An absence of CD8 transcript (fig. S2C) indicated that these cells were not CD4^+^CD8^+^ T cells previously described in CeD (*52*, *53*). While dominated by the expanded clone from HTO4, cluster 7 contained cells from six clones identified across three patients, indicating the generality of this T_H_1-like cytotoxic effector cell phenotype.

In contrast to cells in most other clusters, cells in cluster 5—sourced from all four donors—had lower expression of genes associated with effector function and showed marked up-regulation of genes associated with protein synthesis (e.g., *RPS27*, *RPS30*, *RPL34*, *RLP13*; fig. S2A) as well as higher expression of *FOS*, induced early after TCR signaling (*54*) and *IL7R*, critical for T cell survival, homeostasis, and memory T cell formation (*55*) (Fig. 2B). Alongside the fact that 56% of clones in this cluster were unexpanded, this suggests that cells in cluster 5 have an early activation profile.

### Identification of gliadin epitope-specific T_reg_ cells across all specificities

Intriguingly, cells in cluster 6 expressed transcripts associated with a classical regulatory T cell (T_reg_ cell) phenotype (*56*–*59*), including the up-regulated expression of *FOXP3*, *IL2RA* (CD25), *IL12RB2*, *IKZF2* (HELIOS), *IKZF4*, *RTKN2*, *TNFRSF4* (OX40), *TIGIT*, *MEOX1*, and *HDAC9* along with low *IL7R* (CD127) expression (Fig. 2B) compared to the other clusters. The mean level of CD25 cell surface protein (CSP) expression was also highest in cells associated with cluster 6, relative to CD4 CSP expression in GEX clusters 0 to 7 (fig. S2D). Most of the cells in cluster 6 were specific for glia-α2 (Fig. 2D), corresponding to the dominance of this epitope in the overall CD4^+^ T cell hierarchy (Fig. 1B). The epitope specificity hierarchy of the T_reg_ cells generally reflected that observed for the effector T cells largely found in clusters 0 to 2, with the exception of an absence of glia-α1a/ω1–specific cells (Fig. 2D). Thus, we demonstrate the presence of gliadin epitope–specific CD4^+^ T_reg_ cells that exhibit a T_H_1-like phenotype—characteristic of T_reg_ cells induced by T_H_1 inflammation and primarily involved in the suppression of T_H_1 responses (*60*, *61*)—in the context of CeD and reveal that this phenotype can be found across multiple specificities.

While cells in cluster 6 were derived from three donors, donor HTO2 had the highest relative frequency of cells in this cluster compared to other donors, equating to 6.9% of their gliadin-specific cells (fig. S2E), and also displayed the highest expression of T_reg_ cell transcripts (*IL2RA*, *TNFRSF4*, and *FOXP3*) (Fig. 1F). Intriguingly, this higher relative frequency of T_reg_ cells in donor HTO2 was coincident with low-level production of IFN-γ by CD4^+^ T cells in response to stimulation with gliadin peptides in vitro and the mildest symptoms post wheat challenge compared to the other CeD donors (tables S1 and S2). This finding suggests a potential link between the relative frequency of T_reg_ cell within a patient and disease severity; however, this is not significant association and warrants further investigation in larger, appropriately powered cohorts.

Unlike previous studies (*21*, *22*, *62*), we did not observe clear evidence of follicular B helper T cells (T_FH_) among gluten-specific CD4^+^ T cells in our cohort, with minimal or no expression of characteristic T_FH_ markers *CXCR5*, *BCL6*, *TOX2*, *IL21*, *CXCL13*, and *CD200* (fig. S2F), although *CXCR5* is known to be reduced on T_FH_ cells in active CeD (*21*, *22*). The high expression of canonical T_FH_ markers *ICOS* and *PDCD1* in most GEX clusters is also indicative of activated effector cells.

In summary, gluten-specific CD4^+^ T cells in the blood of the four wheat-challenged CeD patients had not only a transcriptionally mixed phenotype consisting largely of activated T_H_1 effector cells but also a small number of cells, across donors and specificities, with regulatory or cytotoxic properties. While small patient numbers prevent definitive conclusions, differences in the inflammatory profile of activated effector cells and of T_reg_ cell frequency tended to reflect the severity of patient symptoms post-wheat challenge.

### TCR diversity varies between epitope specificities with the DQ2.5–glia-α2 repertoire being most restricted

We performed a detailed analysis of the epitope-specific paired TCRαβ repertoires (table S3) using TCRdist (*63*). Pairwise similarity scores were generated for all unique paired TCR clonotypes in our dataset. The dimensionality of the resulting distance matrix was then further reduced by performing kernel principal components analysis (kPCA) to illustrate the similarity-based TCR clustering of clonotypes within epitope-specific repertoires and their TCRα V (TRAV) gene and TCRβ V (TRBV) gene usage (Fig. 3A). We observed a large, compact cluster of clonotypes characterized by the dominant coexpression of *TRAV26-1* and *TRBV7-2* gene elements in the DQ2.5–glia-α2–specific TCR repertoire (*19*, *20*), as well as a smaller cluster coexpressing *TRAV5* and *TRBV5-1* gene elements (Fig. 3A). We next calculated the weighted average nearest neighbor distances for each clonotype in a given epitope-specific repertoire to measure the density of similar TCRs. This analysis showed DQ2.5–glia-α2–specific TCRs had a lower N-N distance score compared to the other epitopes, indicating a higher degree of convergence in TCRs recognizing DQ2.5–glia-α2 relative to the others (Fig. 3B). Using a similarity-weighted Simpson’s diversity index (TCRdiv) (*63*), we found the DQ2.5–glia-α1a–, DQ2.5–glia-ω2–, DQ2.5–glia-α1a/ω1– and DQ2.5–glia-ω1–specific TCR repertoires were increasingly more diverse, with larger average N-N distances (Fig. 3B) and with dominant clusters being characterized by only one or neither chain (Fig. 3A). For example, TCR similarity (clusters) in the DQ2.5–glia-ω2–specific repertoire appeared to be dictated by the TCRα chain, with clusters of similar TCRs largely defined by *TRAV19*, *TRAV1-2*, or *TRAV4* gene usage (Fig. 3A). While some of these clusters were enriched for particular TRBV elements (e.g., *TRBV2* and *TRBV20-1*), these elements did not define the TCR similarity to the same extent as TRAV usage. In contrast, the DQ2.5–glia-α1a–specific TCR clusters were principally characterized by *TRBV29-1*, *TRBV5*, or *TRBV19* gene elements (Fig. 3A) that were not associated with any particular TRAV gene usage. Preferential usage of the *TRAV1-2*, *TRAV4*, *TRBV29-1*, *TRBV5*, and *TRBV19* gene elements has been described previously for the DQ2.5–glia-ω2– and DQ2.5–glia-α1a–specific repertoires (*13*, *14*). Our clustering analysis provides insights into the similarity of those TCRs within the repertoire that are likely to contain features important for pMHC recognition and extends beyond the expression of particular TRAV or TRBV genes. TCRs generally appeared to be more diverse in both the DQ2.5–glia-ω1– and DQ2.5–glia-α1a/ω1–specific repertoires with larger N-N distances between individual TCRs (Fig. 3, A and B).

**Fig. 3.**
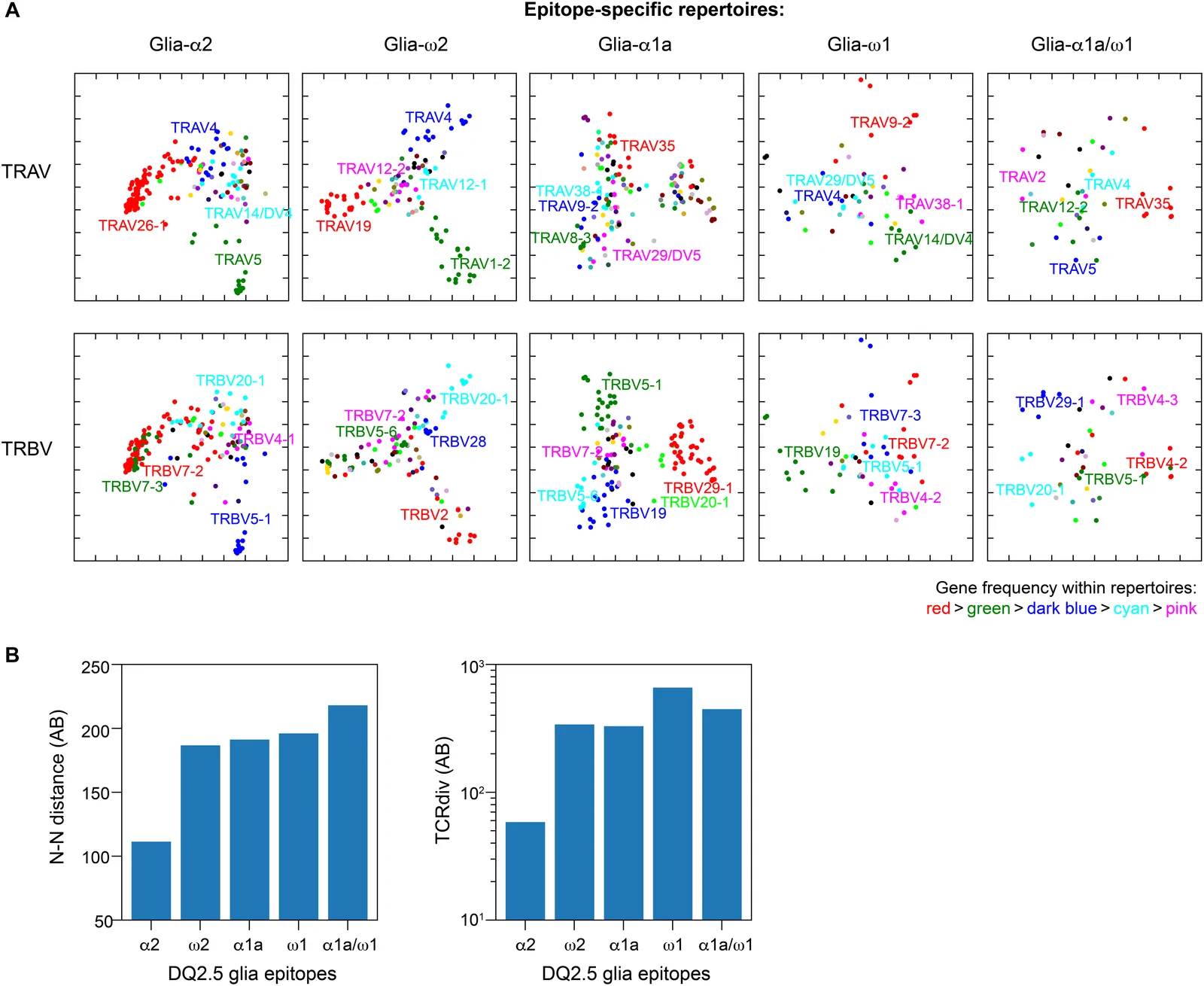
Epitope-specific TCR repertoire diversity reflects distinct features of pMHC recognition. (**A**) Two-dimensional kPCA projection of the TCRdist landscape for each epitope-specific repertoire colored by TCRα V gene (TRAV; top) and TCRβ gene (TRBV; bottom) segment usage. Each point corresponds to a single TCR clone. Colours are based on gene frequency within the repertoire as depicted in key, followed by assorted colours for rare frequencies. (**B**) Nearest-neighbor (N-N) distance between each TCRαβ within an epitope-specific repertoire with its nearest 10% of neighbors, averaged across the entire repertoire (left), and TCRdiv diversity measure, a modified version of the Simpson’s diversity index, incorporating TCR similarity and identity (right), for DQ2.5-gliadin–specific TCRαβ repertoires from table S3.

To extend our observation of TCR clustering and to determine any relationship between TCR clusters and gene expression, we applied the TCR clumping algorithm from the clonotype neighbor graph analysis (CoNGA) package (*26*). We identified 17 TCR clusters (containing >2 clonotypes), each of which corresponded to an epitope specificity, indicating TCR clumping accurately captured particular modes of binding for each of the pMHCs (fig. S2G). Each of the TCR clusters corresponded to at least two of the nine different GEX clusters identified in this analysis and did not show any preferential association with a particular transcriptional profile. Collectively, these data indicate that within epitope-specific TCR repertoires, there are numerous TCR clusters but none of these TCR clusters appeared to direct gliadin-specific CD4^+^ T cells to a particular gene expression profile, suggesting substantial T cell plasticity in CeD, with the transcriptional state appearing to be only weakly constrained by antigen specificity or TCR clonotype.

### Identification of TCR CDR3 motifs defining epitope specificity

For T cell recognition of antigen, the CDR3 region is the most critical and variable loop of the TCR, encompassing the junction between germline-encoded V, (D), and J regions in the TCRα and β chains (fig. S3A). The identification of nongermline-encoded TCR CDR3 motifs statistically enriched within epitope-specific repertoires using the TCRdist algorithm allows the discovery of key conserved residues that have a critical role in mediating TCR recognition (*63*). In contrast to previous studies (*9*, *30*), we have restricted the core motif to residues enriched in the antigen-specific response occurring significantly more frequently than in background sets of V and J gene–matched receptor sequences (*63*). In this way, we identify critical residues that are enriched independently of V- and J-germline–encoded residues. The analysis of CDR3 sequence motifs within the DQ2.5–glia-α2–specific repertoire revealed a strong enrichment of the previously defined nongermline-encoded arginine residue within the CDR3β loop of *TRBV7-2/7-3^+^* TCRs (Fig. 4A) (*18*). We have previously shown this arginine residue acts as the lynchpin for docking of TCRs onto HLA-DQ2.5–glia-α2 (*20*). The enrichment of TCRs using this arginine motif was specific to the DQ2.5–glia-α2–specific repertoire, present across all CeD donors, and was expressed by >50% of clones in the DQ2.5–glia-α2–specific repertoire in two of the four donors (Fig. 4A). A second lower prevalence CDR3β motif was also enriched specifically within the DQ2.5–glia-α2–specific repertoire (Fig. 4B), associated with the TRBV5-1/TRAV5 gene element cluster (Fig. 3A and fig. S3B). While TRAV gene usage was restricted in both the TRBV7-2/TRAV26-1 and TRBV5-1/TRAV5 clusters, we did not identify any enriched nongermline-encoded CDR3α sequence motifs, consistent with our previous demonstration that it is the germline-encoded regions of the TRAV26-1 TCRα chain, rather than its CDR3α loop, that mediates contacts with DQ2.5–glia-α2 (*20*).

**Fig. 4.**
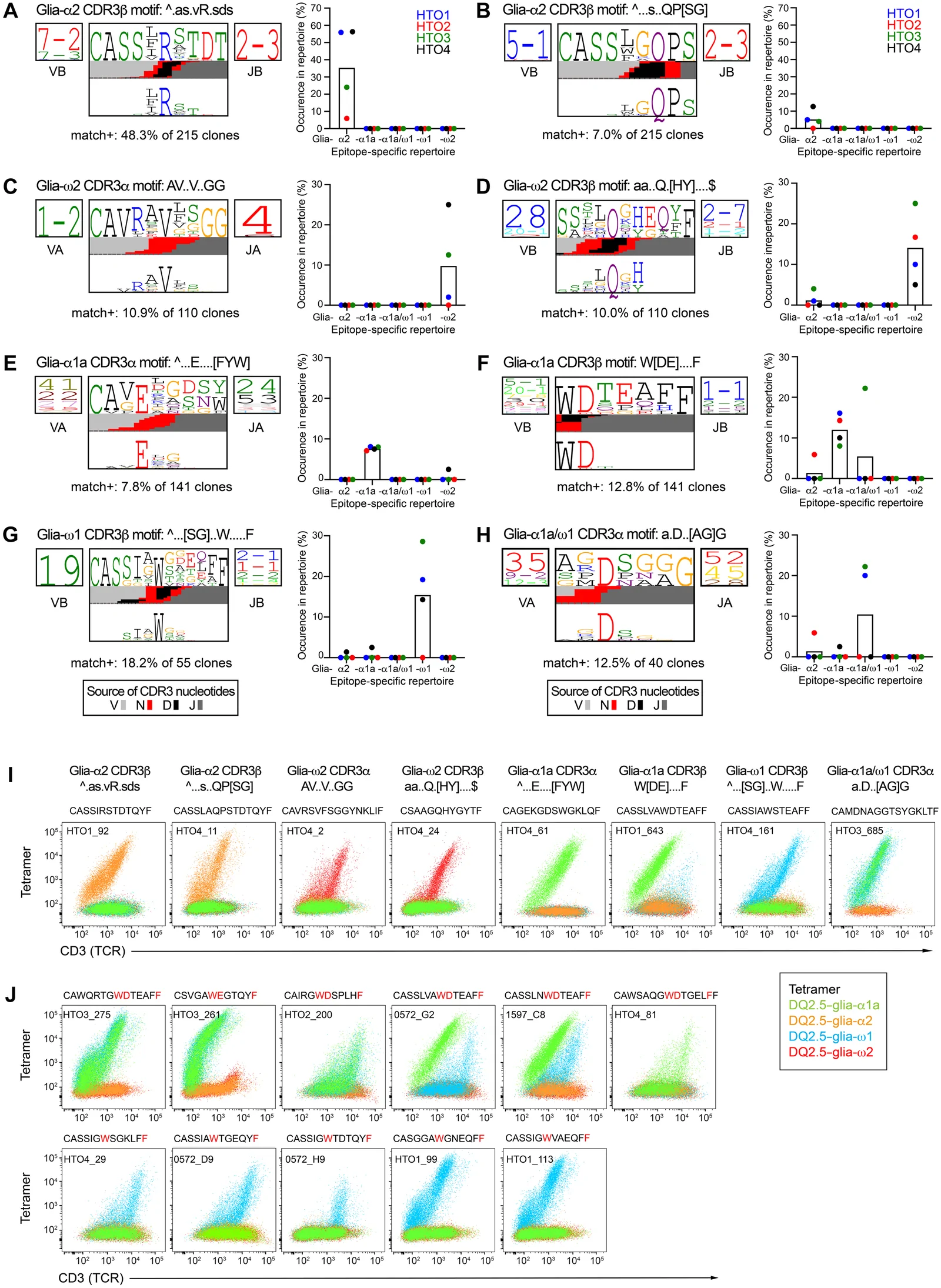
Identification and verification of TCR CDR3 α and β sequence motifs defining epitope specificity. (**A** to **H**) The top scoring CDR3α and/or CDR3β sequence motifs are shown for the DQ2.5–glia-α2– [(A) and (B)], DQ2.5–glia-ω2– [(C) and (D)], DQ2.5–glia-α1a– [(E) and (F)], DQ2.5–glia-ω1– (G), and DQ2.5–glia-α1a/ω1– (H) specific repertoires from CeD patients HTO1 to HTO4 (table S3). Motif symbols include ^ = start of CDR3; a = A,G or S; s = S or T; v = L, F, I, or V; d = D or E; and $ = end of CDR3. All amino acids are indicated in single-letter abbreviations. The TCR logo summarizes V and J usage, CDR3 amino acid enrichment, and inferred rearrangement structures. Motif sequence logos are presented both at full scale (top) and scaled by per-column relative entropy against background frequencies from TCRs with matched gene-segment composition (bottom), highlighting motif positions under selection. For each motif, the proportion of clones expressing it within each epitope-specific repertoire for CeD donor HTO1 (blue), HTO2 (red), HTO3 (green), and HTO4 (black) is also shown. The total number of epitope-specific clones within each donor is shown in table S3A. (**I** and **J**) 293 T cells transiently cotransfected with selected TCR and CD3γδεζ were costained with anti-CD3ε antibody and either DQ2.5–glia-α1a tetramer (green), DQ2.5–glia-α2 tetramer (orange), DQ2.5–glia-ω1 tetramer (blue), or DQ2.5–glia-ω2 tetramer (red), and profiles overlaid. Selected TCRs included (I) representative examples of TCR expressing the CDR3β and CDR3α motifs identified in (A) to (H), isolated from epitope-specific repertoires from CeD donors HTO1 to HTO4 or (J) multiple examples of TCR expressing the CDR3β W[D/E]….F motif (top) or the CDR3β W.….F motif (bottom). TCR details are listed in table S4. Transfected 293T gating strategy is shown in fig. S4.

Given this validation of TCRdist motif identification, we next sought to identify sequence motifs enriched within the other epitope-specific TCR repertoires. Significantly enriched (above background repertoire), non-V/J gene-encoded TCR CDR3α and/or CDR3β motifs were found across the DQ2.5–glia-ω2–, DQ2.5–glia-α1a–, DQ2.5–glia-ω1–, and DQ2.5–glia-α1a/ω1–specific repertoires and in most donors (Fig. 4, C to H). As was observed for the DQ2.5–glia-α2–specific repertoire, the motifs were typically associated with, but not encoded by, a specific V gene element, except for those found in the CDR3α and CDR3β region of the DQ2.5–glia-α1a–specific repertoire, where V gene usage was quite diverse (Fig. 4, E and F). Identified TCR CDR3α and CDR3β motifs within an epitope-specific TCR repertoire were generally found to occur independently of each other. For example, within the DQ2.5–glia-ω2–specific repertoire, TCRs using an enriched valine (V) residue in the CDR3α region that associated with *TRAV1-2* usage (Fig. 4C), clustered independently from TCRs using an enriched glutamine residue followed one amino acid later by a histidine or tyrosine residue (Q.[H/Y]) in the CDR3β region associated largely with *TRBV28* usage (Figs. 4D and 3A). However, codependence between chains was observed; TCRα chains with a glutamic acid (E) residue in the CDR3α region, enriched in the DQ2.5–glia-α1a–specific repertoire (Fig. 4E), appeared to pair almost exclusively with β chains using a *TRBV29-1* gene segment (fig. S3C). No significantly enriched CDR3β sequence motif was identified from these paired β chains, suggesting that, for these TCRs, the CDR3α glutamic acid residue may coordinate with germline encoded regions of the TRBV29-1 TCRβ chain to mediate recognition of the DQ2.5–glia-α1a epitope, similar to that observed between the CDR3β arginine residue and germline-encoded regions of the TRAV26-1 TCRα chain mediating contacts with DQ2.5–glia-α2 (*20*). While TCRs using the CDR3α or CDR3β motif in the DQ2.5–glia-α1a–specific repertoire were present in all four donors (Fig. 4, E and F), in the DQ2.5–glia-ω2–specific repertoire, donors appeared to preferentially use either the CDR3α or CDR3β motif (Fig. 4, C and D).

Two overlapping but distinct CDR3β motifs were revealed for the DQ2.5–glia-α1a- and DQ2.5–glia-ω1–specific repertoires. These consisted of a tryptophan and aspartic (or glutamic) acid (WD or WE) motif in the DQ2.5–glia-α1a–specific repertoire (Fig. 4F) and only a tryptophan (W) residue in the DQ2.5–glia-ω1–specific repertoire (Fig. 4G). The enriched residues for both motifs were encoded by either N additions or the D region and, in both, the tryptophan residue was situated five or six amino acids from the final phenylalanine (F) at the C-terminal end of the CDR3 loop (Fig. 4, F and G). The analysis of the usage of these two CDR3β motifs in all epitope-specific repertoires showed that the W[D/E]….F motif was preferentially used by dextramer-enriched CD4^+^ T cells from the DQ2.5–glia-α1a–specific repertoire (Fig. 4F), while the W…..F motif was preferentially used in the DQ2.5–glia-ω1–specific repertoire (Fig. 4G). While the DQ2.5–glia-ω1–specific W…..F motif was exclusively associated with TCRs using the *TRBV19* gene element, the DQ2.5–glia-α1a–specific W[D/E]….F motif was not linked to any specific TRBV gene element (Fig. 4, F and G). Last, a CDR3α motif consisting of a nongermline-encoded aspartic acid residue (D) was enriched in the DQ2.5–glia-α1a/ω1–specific repertoire, constituting ∼20% of this repertoire in two of the four CeD donors (Fig. 4H). Public TCR sequences previously reported in patients with CeD (*30*) contained some of these identified CDR3 motifs. Here, our precise motif identification pinpoints the specific enriched amino acid motifs, their antigen specificity, and their likely relevance in mediating TCR recognition.

To verify the association between the motifs and specificity, we expressed a selection of motif-bearing TCRs (table S4) in cell lines and stained the TCR transfectants separately with DQ2.5–glia-α1a, DQ2.5–glia-α2, DQ2.5–glia-ω1, and DQ2.5–glia-ω2 tetramers. Specific tetramer binding was observed for each of the motif-bearing TCRs (Fig. 4I and fig. S4). However, a TCR expressing the Glia-α1a–associated CDR3β W[D/E]….F motif (HTO1_643) also bound DQ2.5–glia-ω1 tetramer (Fig. 4I), suggesting that this motif might confer dual DQ2.5–glia-α1a/ω1 specificity. Collectively, these data revealed several novel nongermline-encoded CDR3 motifs strongly associated with DQ2.5-restricted gliadin epitope specificity.

### Enriched TCR CDR3β W[D/E]….F motif predicts DQ2.5–glia-α1a/ω1 cross-reactivity

To explore whether the W[D/E]….F motif drove cross-reactivity to both the DQ2.5–glia-α1a and DQ2.5–glia-ω1 epitopes, we generated cell lines expressing other CDR3β W[D/E] motif-containing TCRs (including two identified from wheat-challenged CeD PBMC by DQ2.5–glia-α1a tetramer-based magnetic enrichment and single-cell index sorting) (table S4) and tested their binding to individual DQ2.5–glia-α1a, DQ2.5–glia-α2, DQ2.5–glia-ω1, and DQ2.5–glia-ω2 tetramers (Fig. 4J, top). All six additional TCR transfectants showed moderate to high levels of DQ2.5–glia-α1a tetramer binding and five of the six also bound equivalent or lower amounts of DQ2.5–glia-ω1 tetramer (Fig. 4J, top). The analysis of steady-state binding affinity of two of the TCR by surface plasmon resonance (SPR) (0572_G2 and HTO2_200) confirmed the high-affinity binding of DQ2.5–glia-α1a and relatively lower-affinity binding of DQ2.5–glia-ω1, i.e., *K*_D_ (dissociation constant) of 6.1 μM versus 37.8 μM and 45.8 μM versus 61.8 μM (Fig. 5A and fig. S5A), corresponding with tetramer staining intensities. These data suggest that the W[D/E]….F motif does confer cross-reactivity and indeed, two of the clonotypes with seemingly equivalent affinity for DQ2.5–glia-α1a and DQ2.5–glia-ω1 tetramers (HTO3_275 and HTO3_261; Fig. 4J, top) showed dual dextramer staining (table S5), contributing 22% (two of nine clones) to the DQ2.5–glia-α1a/ω1–specific repertoire in donor HTO3 (Fig. 4F).

**Fig. 5.**
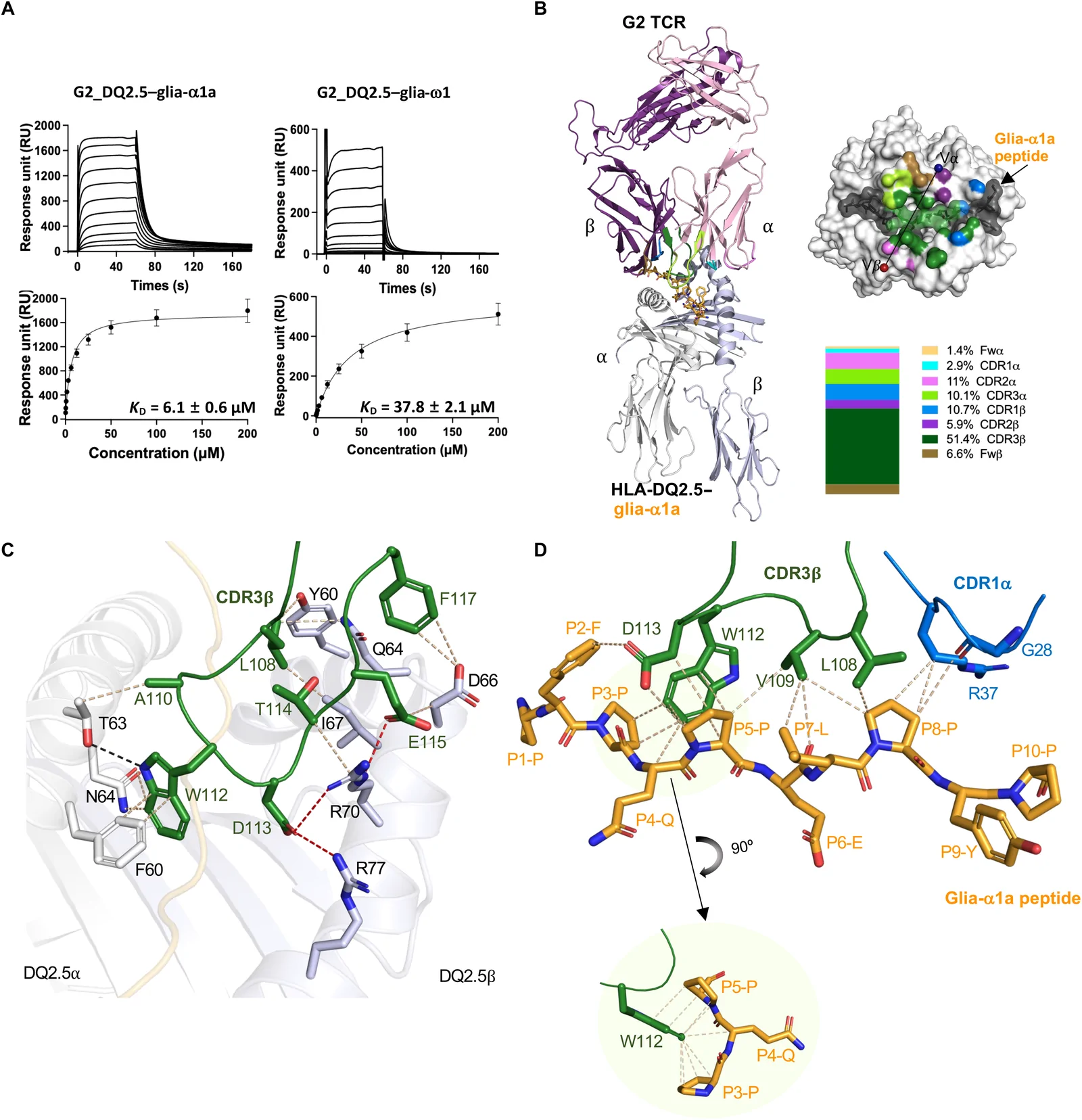
“WD”-motif containing G2 TCR recognition of DQ2.5–glia-α1a. (**A**) Binding analysis of G2 TCR toward DQ2.5–glia-α1a and DQ2.5–glia-ω1 were assessed by SPR. Data were derived from three independent experiments, with equilibrium constant (*K*_D_) calculated using a single ligand-binding model. HLA-DQ8–glia-α1 served as a control for nonspecific binding. Equilibrium response curves were normalized to the theoretical maximum response, and measurements were combined. Data points represent mean values from replicate experiments, with error bars indicating ± SEM. (**B**) Overall structure of the G2 TCR bound to DQ2.5–glia-α1a, with the TCR footprint colored according to the nearest TCR contact residue. The center of mass for Vα and Vβ position is indicated by red and blue spheres, respectively, connected via a black line. Bar chart represents the relative contribution of each CDR loop and FW residues of the G2 TCR to the interface with DQ2.5–glia-α1a. The HLA-DQ2.5 α and β chains are shown in white and light blue, respectively, while the glia-α1a peptide is depicted in orange sticks. CDR loops are highlighted as follows: CDR1α (cyan), CDR2α (violet), CDR3α (light green), CDR1β (blue), CDR2β (purple), and CDR3β (dark green). The FWβ residue is shown in brown. Detailed interactions of G2 TCR with (**C**) DQ2.5 α and β chains and (**D**) glia-α1a peptide are shown. Interface CDR loops, HLA-DQ2.5, and glia-α1a residues are displayed in sticks and colored as in (B). P-W^112^-P stacking is highlighted in pale yellow and rotated at 90°. The H bonds, vdW, and salt bridges are displayed as black, light beige, and red dashed lines, respectively. All amino acids are indicated in single-letter abbreviations.

Despite this association with DQ2.5–glia-α1a and DQ2.5–glia-ω1 cross-reactivity, most of the CD4^+^ T cells expressing the TCR CDR3β W[D/E]….F motif were designated as uniquely DQ2.5–glia-α1a specific based on dextramer binding (table S5). As the TCR transfectants revealed, most of these TCRs bound the DQ2.5–glia-α1a epitope with an equivalent or higher affinity. Thus, we speculate that competition between the pooled dextramers for TCR binding during the original staining (fig. S1B) may have resulted in undetectable DQ2.5–glia-ω1 dextramer binding by many of the clones.

TCRs using the overlapping but distinct W.….F sequence motif identified within the DQ2.5–glia-ω1–specific repertoire (Fig. 4G) were observed to bind the DQ2.5–glia-ω1 tetramer exclusively (Fig. 4J, bottom), with analysis of steady-state binding affinity of two CDR3β W.….F motif–expressing TCRs (0572_D9 and 0572_H9) showing moderate affinity for DQ2.5–glia-ω1 and very weak or negligible binding affinity for DQ2.5–glia-α1a, i.e., *K*_D_ of 65 μM versus 246 μM and 132 μM versus not detected (fig. S5, B and C). Collectively, this analysis has unveiled at a high degree of resolution, core CDR3 motifs that preferentially confer gliadin-epitope specificity and one that confers TCR cross-reactivity between DQ2.5–glia-α1a and DQ2.5–glia-ω1.

### Structural basis for cross-reactivity conferred by the CDR3β W[D/E] motif

To investigate the structural bases of DQ2.5–glia-α1a and DQ2.5–glia-ω1 cross-reactivity by TCRs containing the W[D/E]….F motif, we first determined the crystal structure of the TRAV35^+^-TRBV5-1^+^ 0572A1_G2 (WD) TCR (table S4), hereafter called G2 TCR, in complex with DQ2.5–glia-α1a at a resolution of 3.2 Å, with a well-defined electron density map at the TCR-pHLA interface (Fig. 5B, fig. S6A, and table S6). The G2 TCR adopted a canonical docking orientation over the central region of the pHLA complex, with a total buried surface area (BSA) of ∼2000 Å^2^ (Fig. 5B and table S7). The interaction was predominantly mediated by the TCR Vβ chain, which contributed ∼74% of the total BSA, while the Vα chain accounted for the remaining 26% (Fig. 5B). Within the Vβ chain, the CDR3β loop was the major contributor, accounting for ∼51% of the total BSA (Fig. 5B and table S7). The CDR3β loop traversed the central axis of both the glia-α1a peptide and HLA-DQ2.5, underscoring its critical role in pHLA recognition (Fig. 5, C and D). Extensive interactions were observed between seven CDR3β residues and both the HLA-DQ2.5 α chain and β chain (Fig. 5C and table S8). In addition to the HLA-interactions, CDR3β residues also made extensive contacts with glia-α1a peptide. Notably, within the ^112^WD^113^ motif, W^112^ engaged in rigid π-π stacking with F^60^ and hydrogen bonding (H bond) with T^63^ of DQ2.5 α chain, while simultaneously contacting P3 and P5 proline residues of glia-α1a peptide via a P-W-P aromatic complex (Fig. 5, C and D, and table S8). The cyclic arrangement of prolines minimizes dispersive contacts with the aromatic partner, thereby enhancing interface stability and molecular recognition (Fig. 5D) (*64*). Concurrently, CDR3β D^113^ formed salt bridges with R^70^ and R^77^ of the DQ2.5 β chain and established van der Waals contacts with glia-α1a P2-F and P5-P residues (Fig. 5, C and D, and table S8). Thus, the ^112^WD^113^ motif played a central role in engaging the N-terminal region of the peptide, a region which is conserved between the glia-α1a and glia-ω1 peptides. Further stabilizing contacts were contributed by V^109^ and L^108^ of CDR3β with P5-P, P7-L, and P8-P of glia-α1a peptide, while CDR1β residues (G^28^ and R^37^) also reinforced peptide binding via interactions with P8-P (Fig. 5D and table S8). Together, these interactions create a dense noncovalent network that locks the G2 TCR onto the DQ2.5–glia-α1a complex.

We next determined the crystal structure of G2 TCR-DQ2.5–glia-ω1 complex at 3.2 Å resolution (fig. S6, B and C, and table S6). This complex adopted a docking topology nearly identical to that of the G2 TCR-DQ2.5–glia-α1a complex (fig. S6C). Structural alignment of the two complexes based on the Cα backbone of DQ2.5 revealed a highly conserved recognition pattern, with a root mean square deviation value of 0.3 Å (fig. S6D and table S7). Notably, the glia-α1a and glia-ω1 peptides share ∼82% sequence identity (*14*, *65*), differing only at positions P7 and P9, whereby P7-L and P9-Y in glia-α1a are substituted with P7-Q and P9-F in glia-ω1 peptide, respectively (Fig. 6A). Because of high sequence identity at the N-terminal part of peptide (P1-P5), the G2 TCR-DQ2.5–glia-ω1 complex displayed a conserved mode of recognition, mediated by the ^112^WD^113^ motif, in both complexes (Fig. 6B and table S9). While the N-terminal regions of both peptides docked similarly, the glia-ω1 peptide was positioned deeper within the antigen binding groove, driven by the hydrophobic P9-F residue, resulting in a ∼1.2-Å shift (Fig. 6C). Moreover, the polar P7-Q side chain of glia-ω1 disrupted the hydrophobic moiety formed by V^109^ and L^108^ in the CDR3β loop, leading to partial contact loss (Fig. 6C). These structural differences likely account for the ∼6-fold discrepancy in TCR-pHLA affinity between the interactions. To functionally validate the structural role of the ^112^WD^113^ motif, we performed alanine mutagenesis of individual residues within the motif and assessed their binding to DQ2.5–glia-α1a and DQ2.5–glia-ω1 using SPR (Fig. 6D and fig. S7). The mutation of CDR3β S^107^, which does not interact with either the peptide or HLA, was included as a negative control. As expected, alanine substitution of either W^112^ or D^113^ severely impaired binding, completely abolishing recognition of both pHLA complexes (Fig. 6D and fig. S7).

**Fig. 6.**
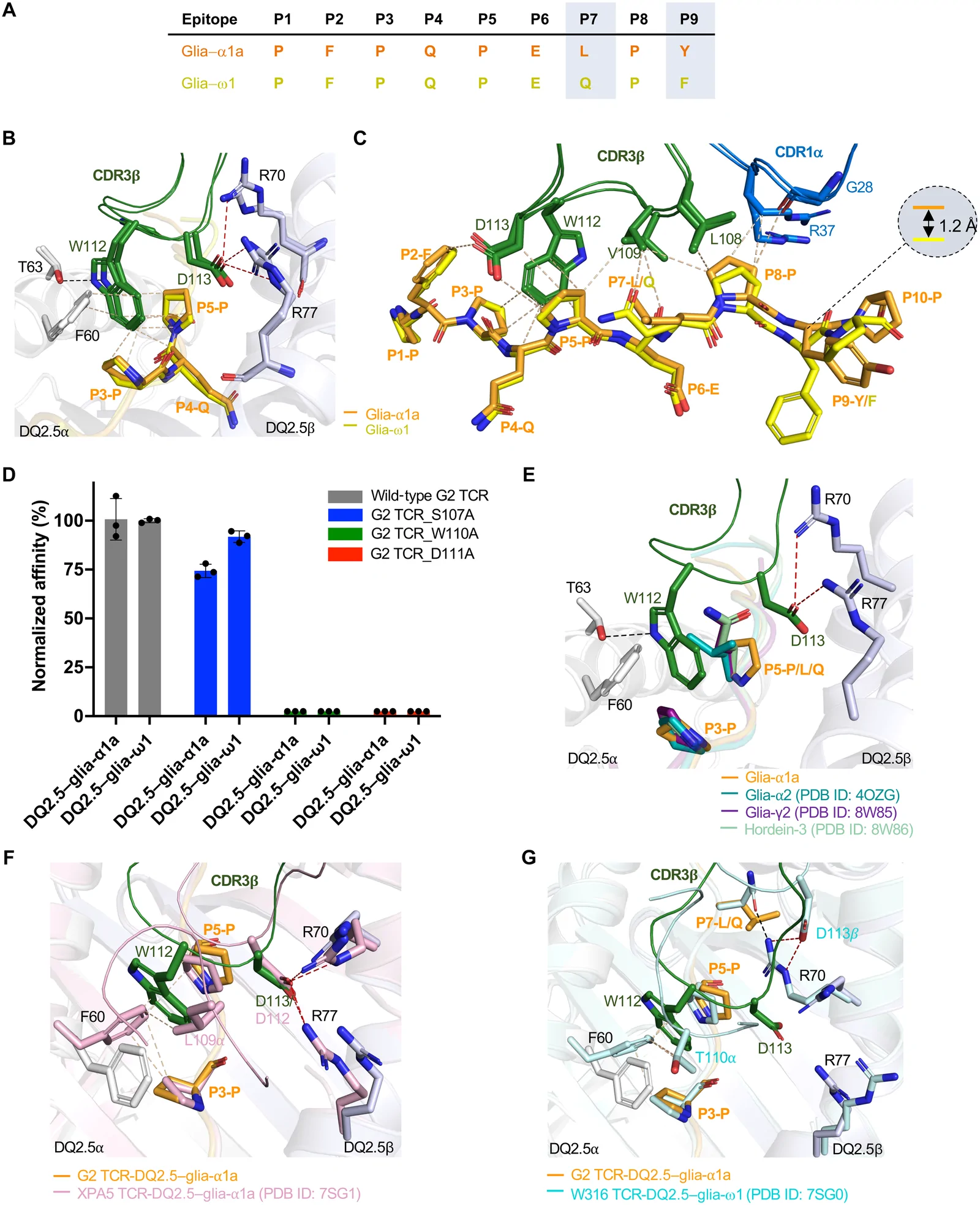
G2 TCR cross-reactivity with DQ2.5–glia-α1a and DQ2.5–glia-ω1 and structural comparison with related TCRs. (**A**) Peptide sequences of DQ2.5–glia-α1a and DQ2.5–glia-ω1 spanning pockets 1 to 9. The discrepancy between DQ2.5–glia-α1a and DQ2.5–glia-ω1 at position 7 and 9 are highlighted in gray. (**B**) Overlay of G2 TCR ^112^WD^113^ motif (CDR3β, dark green) interactions with DQ2.5–glia-α1a and DQ2.5–glia-ω1 complexes. The HLA-DQ2.5 α and β chains are shown in white and light blue, respectively, while the glia-α1a and glia-ω1 peptides are depicted in orange and yellow sticks, respectively. (**C**) Structural comparison of G2 TCR-peptide contacts in the glia-α1a and glia-ω1 complexes. CDR loop and peptide residues are displayed as sticks [colored as in (B)], with CDR1β in blue. Deviations between glia-α1a and glia-ω1 peptides are highlighted and measured (unit in Å). The H bonds (black), vdW (beige), and salt bridges (red) are indicated by dashed lines. Residues are annotated using single-letter amino acid codes. (**D**) Effect of single point mutations within the ^112^WD^113^ motif on binding affinity to DQ2.5–glia-α1a and DQ2.5–glia-ω1 as measured by SPR. Data represent normalized affinity strength relative to wild-type G2 TCR (*n* = 3 independent experiments) on the *y* axis. The *x* axis indicates the mutated positions. (**E**) Structural overlay of ^112^WD^113^ motif interactions in the G2 TCR-DQ2.5–glia-α1a with published TCR-DQ2.5 complexes involving alternative gluten peptides: glia-α2 (deep teal; PDB ID: 4OZG), glia-γ2 (purple; PDB ID: 8 W85), and hordein-3 (pale green; PDB ID: 8 W86). (**F** and **G**) Structural comparison of TCR-pMHC interactions in the G2 TCR-DQ2.5–glia-α1a complex with the (F) XPA5 TCR-DQ2.5–glia-α1a complex (light pink; PDB ID: 7SG1) and (G) W316 TCR-DQ2.5–glia-ω1 complex (pale cyan; PDB ID: 7SG0). CDR loops and peptide residues are displayed as sticks (colored as in (B). The H-bonds, vdW, and salt bridges are depicted as black, light beige, and red dash lines, respectively.

To understand why the cross-reactivity of the G2 TCR is confined to glia-α1a and glia-ω1, we structurally aligned other DQ2.5-gluten peptide complexes, including glia-α2, glia-γ2, and hordein-3 (Fig. 6E and table S10). In these peptides, the P5-P residue is replaced by P5-L (glia-α2) or P5-Q (glia-γ2 and hordein-3), thereby disrupting the ^112^WD^113^ mediated interactions and limiting cross-reactivity (Fig. 6E). Together, these findings demonstrate that ^112^WD^113^ motif plays a central role in mediating high-affinity binding and cross-reactivity between glia-α1a and glia-ω1 peptides presented by HLA-DQ2.5.

To contextualize these findings, we aligned the structures of the two G2 TCR complexes (with glia-α1a and glia-ω1) with published DQ2.5–glia-α1a/ω1 complexes involving XPA5 and W316 TCRs (*66*). The TRAV4^+^-TRBV29-1^+^ (CDR3α CLVGGLARDMRF, CDR3β CSVALGSDTGELFF) XPA5 TCR exhibits DQ2.5–glia-α1a/ω1 cross-reactivity, contains no W residue, and has a D residue that sits in a −1 position relative to the WD motif in the G2 TCR. The TRAV9-2^+^–TRBV7-3^+^ (CDR3α CALSGGTSYGKLTF and CDR3β CASSQGQDTEAFF) W316 TCR on the other hand is uniquely reactive to DQ2.5–glia-ω1 and contains a D residue in the exact same position as the G2 TCR but no W residue. In the XPA5 TCR complex, the conserved CDR3β D^112^ residue formed salt bridges with DQ2.5 β chain R^70^ and R^77^ (Fig. 6F), as was observed for G2 TCR. Notably, while lacking the W^112^ observed in the G2 TCR, the XPA5 TCR harbors a hydrophobic L^109^ residue within its CDR3α loop at an equivalent structural position, which provides compensatory interactions with F^60^ of the DQ2.5 α chain (Fig. 6, E and F). Although L^109^ did not contact the peptide, the smaller side chain induced a rearrangement of the F^60^ side chain to coaccommodate XPA5 TCR binding to P3-P and P5-P of glia-α1a peptide, thereby maintaining the hydrophobic network between the TCR-DQ2.5–glia-α1a peptide complex and hence cross-reactivity to glia-ω1 peptide (Fig. 6F).

Although the W316 TCR also encodes D^113^ in CDR3β loop, its Vα and Vβ domains are completely repositioned relative to the G2 TCR (fig. S8). The CDR3α loop of W316 TCR occupies the spatial position of the ^112^WD^113^ motif in G2 TCR, while the CDR3β loop bridges peptide positions P5 to P7 (Fig. 6G). Here, D^113^ formed a salt bridge with R^70^ of the DQ2.5 β chain, which reoriented to H-bond with P7-Q of the glia-ω1 peptide (Fig. 6G). These interactions would be disrupted by P7-L (as in the glia-α1 peptide), thereby preventing W316 TCR cross-reactivity (Fig. 6G). In addition, the smaller side chain of T^110^ (CDR3α) in the W316 TCR, which occupies the position of G2 TCR’s W^112^, allowed F^60^ repositioning akin to XPA5 TCR complex, and did not contact with peptide directly, reaffirming the recognition is exclusive to glia-ω1 peptide (Fig. 6G).

Collectively, the positioning of D^113^ within the CDR3β loop enables salt bridge formation with R^70^ of the DQ2.5 β chain, thereby inducing a rearrangement of R^70^ that underlies cross-reactivity between glia-α1a and glia-ω1 peptide (Fig. 6, F and G). In G2 and XPA5 TCRs, D^113/112^ is orientated at the P3 to P5 region, stabilized by complementary hydrophobic counterparts—W^112^ in G2 or L^109^ in XPA5, which lock between P3 to P5 prolines of the peptide and F^60^ of the DQ2.5 α chain. This configuration promotes a shift of R^70^ toward the peptide N terminus, allowing corecognition of both glia-α1a and glia-ω1 peptides. Whereas in the W316 TCR, D^113^ docks at P5 to P7, where its salt bridge contact with R^70^ reorients the R^70^ side chain toward the peptide C terminus. This orientation enables hydrogen bonding with P7-Q of glia-ω1 while discriminating against P7-L of glia-α1a, thereby conferring peptide selectivity.

### Structural basis of DQ2.5–glia-ω2 recognition by a TCR encoding a conserved CDR3β glutamine residue

To investigate how the conserved CDR3β glutamine residue (Fig. 4D) contributes to DQ2.5–glia-ω2 recognition, we selected a TCR using TRAV14/DV4 and TRBV5-4 gene segments, which appeared in both HT01 (clone C82) and HT04 (clone 227) patients. This clone is hereafter referred to as the C82 TCR (table S4). The C82 TCR CDR3β also contains a tyrosine residue, also enriched in the glia-ω2 CDR3β motif defined by TCRdist (aa.Q.[HY]….$) (Fig. 4D), although positioned adjacent to the glutamine residue and five residues from the C-terminal end of the CDR3 loop. To determine the affinity and specificity of this C82 TCR, we performed SPR steady-state binding analyses against DQ2.5-glia presenting either the glia-ω2 or glia-α2 epitope. The C82 TCR bound DQ2.5–glia ω2 with relatively high affinity (*K*_D_ ∼ 11 μM) but show no detectable binding to DQ2.5–glia-α2, indicating epitope specificity (Fig. 7A).

**Fig. 7.**
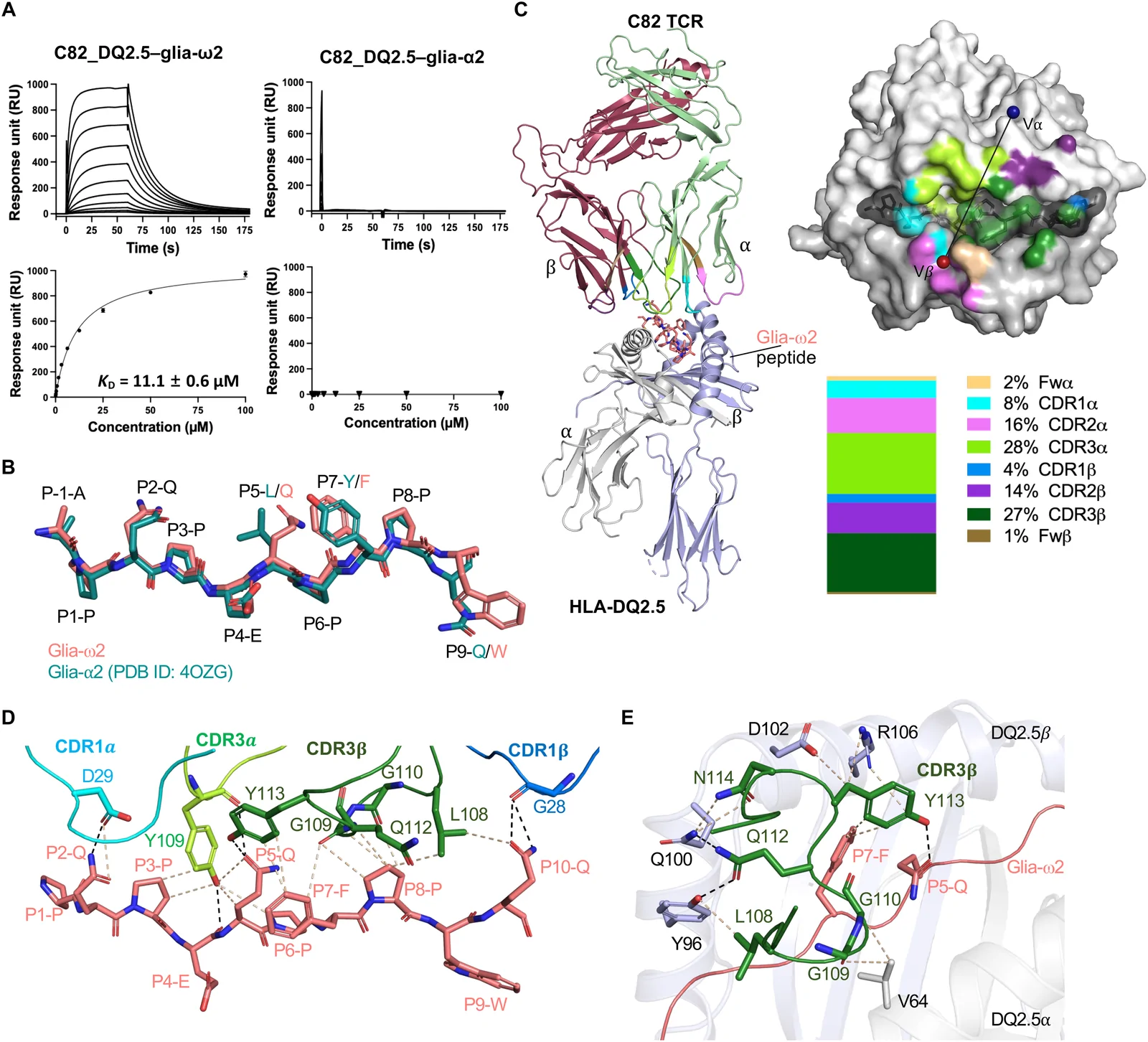
Recognition of DQ2.5–glia-ω2 by C82 TCR containing “Q” residue. (**A**) Binding analysis of C82 TCR toward DQ2.5–glia-ω2 and DQ2.5–glia-α2 were assessed by SPR. Data were derived from three independent experiments with equilibrium constant (*K*_D_) calculated using a single ligand binding model. HLA-DQ8–glia-α1 served as a control for nonspecific binding. Equilibrium response curves were normalized to the theoretical maximum response, and measurements were combined. Data points represent mean values from replicate experiments, with error bars indicating ± SEM. (**B**) Structural overlay of glia-ω2 peptide with the published D2 TCR-DQ2.5–glia-α2 peptide (deep teal; PDB ID: 4OZG). (**C**) Overall structure of the C82 TCR bound to DQ2.5–glia-ω2, with the TCR footprint colored according to the nearest TCR contact residue. The center of mass for Vα and Vβ position is indicated by red and blue spheres, respectively, connected via a black line. Bar chat represents the relative contribution of each CDR loop and FW residues of the C82 TCR to the interface with DQ2.5–glia-ω2. The HLA-DQ2.5 α and β chains are colored white and light blue, respectively, with the glia-ω2 peptide in pink sticks. CDR loops are highlighted as follows: CDR1α (cyan), CDR2α (violet), and CDR3α (light green), FWα (wheat), CDR1β (blue), CDR2β (purple), CDR3β (dark green), and FWβ (brown). (**D**) Detailed interface of C82 TCR with glia-ω2 peptide and (**E**) CDR3β loop with DQ2.5 α and β chains. The H bonds and vdW are displayed as black and beige lines, respectively. All amino acids are indicated in single-letter abbreviations.

Among the four immunodominant gluten epitopes, glia-ω2 and glia-α2 are highly homologous, sharing ∼64% sequence identity and differing at only three positions (P5, P7, and P9). Specifically, P5-Q, P7-F, and P9-W in glia-ω2 are substituted by P5-L, P7-Y, and P9-Q in glia-α2, respectively (Fig. 7B). While the P9 residue serves primarily as an MHC anchor, the P5 and P7 side chains protrude from the peptide-binding groove and are accessible for TCR engagement. Hence, the altered physicochemical properties introduced by the P5-Q/L and P7-F/Y substitutions are likely to underpin the differential recognition of glia-ω2 and glia-α2 by the C82 TCR (fig. S9A).

To define the molecular basis of this specificity, we determined the crystal structure of the C82 TCR-DQ2.5–glia-ω2 ternary complex at 3.25-Å resolution (Fig. 7C and table S6). The glia-ω2 peptide was resolved in the electron density map, enabling unambiguous modeling of peptide conformation and TCR contacts (fig. S9B). The C82 TCR adopted a canonical docking mode over the peptide-MHC complex, burying ∼1960 Å^2^ of solvent-accessible surface area at the interface. The Vα and Vβ domains contributed 54 and 46% of the BSA, respectively (Fig. 7C and table S7). The analysis of the TCR footprint revealed comparable contributions from the CDR3α and CDR3β loops, accounting for 28 and 27% of the total BSA, respectively. Within the glia-ω2 peptide-TCR contacting interface, the solvent-exposed P5-Q and P7-F formed extensive interactions with both CDR3 loops. P5-Q formed H bonds with Y^109^ and Y^113^ from the CDR3α and CDR3β loops, respectively. In contrast, P7-F and the adjacent P8-P residues were predominantly engaged by CDR3β residues including L^108^, G^109^, G^110^, and Q^112^ (Fig. 7D and table S11).

Although the conserved CDR3β residue Q^112^ did not directly contact the peptide, it occupied a central position within an extensive interaction network involving the CDR3β residue loop, the DQ2.5 β chain, and the key peptide residues P5-Q and P7-F (Fig. 7E). Specifically, Q^112^ and N^114^ were oriented toward a polar environment formed by DQ2.5β residues Y^96^ and Q^100^, establishing favorable electrostatic interactions. In contrast, Y^113^ was positioned between P5-Q and P7-F of the glia-ω2 peptide. The hydroxyl group of Y^113^ formed an H bond with P5-Q, while its aromatic ring was stabilized through hydrophobic packing against P7-F (Fig. 7E). This arrangement creates a highly complementary interaction network across the center of the peptide-binding groove, enabling precise recognition of glia-ω2 by C82 TCR. Substitution of P5-Q and P7-F with P5-L and P7-Y, as found in glia-α2, would be expected to alter both the electrostatic and steric environment surrounding Y^113^, thereby disrupting this optimized interaction network. Collectively, these findings suggest that the conserved CDR3β glutamine residue contributes indirectly to antigen discrimination by positioning the adjacent Y^113^ residue for productive engagement of the glia-ω2 epitope, thereby enabling selective recognition of glia-ω2.

### Enriched CDR3 TCR motifs predict epitope specificity

We have identified enriched TCR CDR3 motifs within epitope-specific repertoires and validated their specificity through TCR expression studies, indicating their potential to serve as predictors of epitope specificity. To test their predictive potential, we analyzed a previously published TCR sequence database derived from 50 patients with CeD (*30*) that included 2129 clonotypes isolated using a pooled cocktail of tetramers for all four immunodominant wheat epitopes and smaller numbers of TCRs of defined epitope specificity due to their isolation with individual tetramers. All of the TCR CDR3 motifs identified in our study (Fig. 4, A to H) could be observed at varying prevalences in the pool of clones captured with all four tetramers, and from multiple patients, with the DQ2.5–glia-α2–specific CDR3β arginine motif in 11.8% of clonotypes, matching the previously described frequency of 10.5 to 13.3% in the entire dataset (Fig. 8A) (*30*). Notably, the frequency of clonotypes using six of the eight epitope-specific CDR3 motifs increased specifically in the pool of cells captured with the individual tetramer(s) associated with that epitope specificity and generally at the frequency we observed in our TCR repertoire dataset (Figs. 8A and 4, A and D to H). Interestingly, the CDR3β W[D/E]….F motif that we have shown to be cross-reactive to the DQ2.5–glia-α1a and DQ2.5–glia-ω1 epitopes was again preferentially selected with the DQ2.5–glia-α1a tetramer and, to a lesser extent, with DQ2.5–glia-ω1 tetramer (Fig. 8A). The frequency of patients with CDR3 motif-positive clonotypes varied by the motif, with 93% of the 30 patients with CeD who contributed to the pool of cells captured with all tetramers containing the DQ2.5–glia-α2–associated CDR3β R motif (Fig. 8A). Two-thirds of these 30 patients had clonotypes expressing at least four of the identified motifs, indicating that these motifs are broadly prevalent across individuals. The CDR3 motifs were detected in both single and expanded clonotypes. To confirm that the CDR3 motifs observed in the pooled tetramer-binding dataset (*30*) conferred the predicted epitope specificities, we selected motif-bearing TCRs from this set (table S4) and tested their specificity after in vitro expression by individual DQ2.5–glia-α1a, DQ2.5–glia-α2, DQ2.5–glia-ω1, and DQ2.5–glia-ω2 tetramer staining (Fig. 8B). The DQ2.5–glia-α2–specific enriched arginine motif was not included as its specificity is well established (*18*, *20*). Epitope specificity was confirmed for each of the TCR transfectants (Fig. 8B), including cross-reactivity for DQ2.5–glia-α1a/ω1 for the TCR expressing the CDR3β W[D/E]….F motif, once again validating that our identified CDR3 motifs can predict epitope specificity. Unexpectedly, the TCR using the Glia-ω2 CDR3β Q.[H/Y] motif showed a degree of cross-reactivity with DQ2.5–glia-α2, in contrast to the TCR using the same CDR3β motif in Fig. 4I, which was specific for just DQ2.5–glia-ω2. The individual tetramer staining of further TCRs using the CDR3β Q.[H/Y] motif suggests that cross-reactivity with DQ2.5–glia-α2 is better associated with the expression of *TRAV12-1–TRAJ3–TRBV28* gene elements, rather than the CDR3β Q.[H/Y] motif itself (fig. S10). In summary, our results suggest that the enriched TCR core CDR3 sequence motifs can predict epitope specificity and cross-reactivity and could prove useful as diagnostic indicators for CeD.

**Fig. 8.**
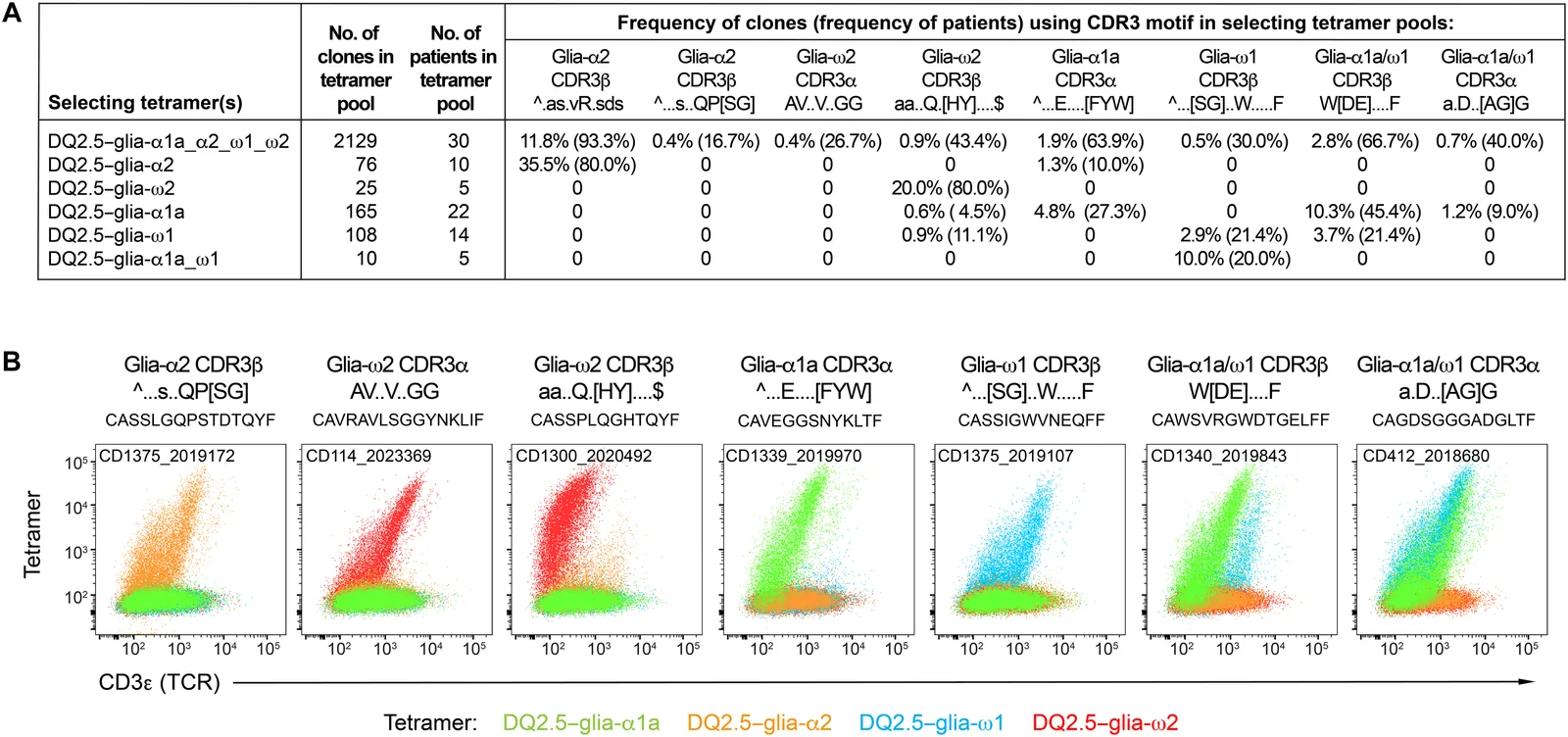
Enriched TCR CDR3 motifs predict epitope specificity in a partially assigned gluten-specific TCR database. (**A**) Frequency of clonotypes using the epitope-specific enriched TCR CDR3α and CDR3β sequence motifs from Fig. 4A-H in CD4^+^ T cells selected from PBMC or lamina propria T cells of gut biopsies from CeD patients or in vitro cultured T cell lines with a pooled cocktail of tetramers to DQ2.5–glia-α1a, to DQ2.5–glia-α2, to DQ2.5–glia-ω1, and to DQ2.5–glia-ω2 (DQ2.5–glia-α1a_α2_ω1_ω2) or with pooled tetramers to DQ2.5–glia-α1a and -ω1 (DQ2.5–glia-α1a_ω1) or with individual tetramers, published in (*30*). Also shown in brackets is the frequency of patients with specific motif-positive clonotypes among all patients with CeD who contributed to the selecting tetramer pool. (**B**) 293T cells were transiently cotransfected with CD3γδεζ and selected TCRs isolated from the DQ2.5–glia-α1a_α2_ω1_ω2 pool, which contained the CDR3 motifs from Fig. 4(B to H). TCR-transfected cells were costained with anti-CD3ε and either DQ2.5–glia-α1a tetramer (green), DQ2.5–glia-α2 tetramer (orange), DQ2.5–glia-ω1 tetramer (blue), or DQ2.5–glia-ω2 tetramer (red), and profiles were overlaid. TCR details are listed in table S4. Gating strategy is shown in fig. S4.

## DISCUSSION

Although more than 20 gluten-derived CD4^+^ T cell epitopes have been identified in CeD, a substantial fraction of the gluten-specific response targets the four HLA-DQ2.5–restricted immunodominant gliadin epitopes studied here. These T cell responses are broadly codominant within individuals, although the relative dominance can vary depending on the source of gluten exposure (*11*, *12*, *32*). To date, transcriptional analyses of these CD4^+^ T cells has largely focused on nonantigen-specific (*41*, *62*, *67*–*70*) or pooled antigen-specific populations from patients with CeD (*21*–*23*, *41*, *67*–*69*). While informative, this has prevented detailed analyses of transcriptional or effector signatures associated with particular epitope specificities. Moreover, while TCR usage has been extensively characterized in gliadin-specific T cells, defining signatures underlying epitope specificity and epitope cross-reactivity remain incompletely resolved. Here, we have comprehensively mapped the transcriptional profile and paired αβTCR usage of CD4^+^ T cells specific for each of the four immunodominant DQ2.5 gliadin epitopes in CeD patient PBMCs mobilized from the gut after oral wheat challenge. As with all droplet-based scRNA-seq approaches, the 10x Genomics platform has lower per-cell transcript capture efficiency compared with plate-based methods, which may underrepresent low-abundance transcripts. However, the substantially higher throughput afforded by 10x enables robust sampling across the full spectrum of gliadin-specific CD4^+^ T cell states, which is essential for resolving phenotypic heterogeneity and rare subsets. At the sequencing depth used here, key populations and T cell transcriptional programs were reproducibly detected, supporting the suitability of this approach for comparative and population-level analyses.

While diversity in transcriptional profiles was observed for antigen-specific CD4^+^ T cells, this was not associated with the various epitope specificities. Most of the profiles identified—effector T_H_1 and T_H_1/T_H_17, T_reg_ cell, and early activated T cells—were found across all epitope specificities and at roughly similar proportions indicating that the distinct nature of epitope recognition in CD4^+^ T cells was not driving functional specialization. There was also little association between transcriptional profile and TCR usage even within epitope specificities. By contrast, there are examples in both infectious and autoimmune settings where CD4 T cell lineage and functional differentiation are constrained by epitope specificity and/or TCR repertoire (*25*–*29*). Our finding suggests that, in CeD, environmental cues or local tissue factors may exert an unusually dominant influence over CD4 T cell function, effectively decoupling TCR clonotype from transcriptional phenotype. Most of the epitope-specific CD4^+^ T cells reflected a highly differentiated, inflammatory phenotype previously described across blood and gut of untreated patients with CeD and in blood after gluten challenge (*21*–*23*), as well as in other autoimmune conditions including systemic lupus erythematosus, systemic sclerosis (*21*), and rheumatoid arthritis (*71*). We show here that this activated effector memory phenotype is highly consistent both within and across the four distinct gliadin epitope specificities.

In contrast to a previous study suggesting that up to 80% of ex vivo circulating gluten-specific antigen-stimulated CD4^+^ T cells in wheat-challenged CeD patients were Foxp3^+^ CD39^+^ T_reg_ cells (*72*), our study identified only a small population of CD4^+^CD25^high^ T cells with a transcriptional phenotype of classical CD4^+^CD25(*IL2RA*)^+^CD127(*IL7R*)^−^Foxp3^+^ regulatory T cells (∼2% of total gluten-specific CD4^+^ T cells). The similar frequency of T_reg_ cells across specificities indicated that no epitope-specific population showed preferential differentiation into T_reg_ cells. Intriguingly, at least based on dextramer UMI information, the avidity of the T_reg_ cells for antigen was not higher than their conventional T cell counterparts, suggesting that they are likely to have been peripherally induced, rather than developing via high affinity TCR-pMHC interactions in the thymus (*73*). Multiple previous studies have shown that oral exposure to dietary antigens is effective at inducing T_reg_ cells and may play an important role in establishing tolerance to food antigens (*74*–*77*).

Our transcriptional analysis also revealed a small but distinct population of cytotoxic CD4^+^ T cells, characterized by high expression of Eomes, granzyme K (GzmK), and granzyme A (GzmA), specific for three epitopes but largely dominated by a single DQ2.5–glia-ω1–specific clone. In contrast, a previous study showed marked down-regulation of cytotoxic marker genes *GZMA* and *GZMK* in pooled tetramer-positive compared to tetramer-negative cells (*23*). CD4^+^ cytotoxic T lymphocytes (CTLs) have become increasingly linked with autoimmunity in addition to viral infections and antitumor activity (*49*). The phenotype of the cytotoxic CD4 T cells observed in our study—with high-level expression of GzmK, GzmA, (not GzmB) and checkpoint molecules including TIGIT and CTLA4—corresponds more closely with the long-lived (>10 years) CD4^+^ CAR T cells found in patients that have achieved long-term remission from leukemia (*78*). The cells in that study were associated with chronic antigen stimulation but were still highly functional and capable of mediating cytotoxicity. Thus, the presence of these cytotoxic CD4 T cells in patients with CeD suggests that they may be contributing to the inflammatory pathogenesis of celiac disease.

While transcriptional profiles did not differ based on epitope specificity, they did differ between CeD donors. Epitope-specific CD4^+^ T cells with a highly inflammatory transcriptional profile were observed from one patient, coincident with numerical dominance, higher-level production of IFN-γ by CD4^+^ T cells in response to stimulation with gliadin peptides in vitro and more severe symptoms post-wheat challenge. In contrast, we observed an increased frequency of T_reg_ cells together with more conventional T_H_1 cells in the patient with low IFN-γ levels and mild symptoms upon gluten challenge. The analysis of a larger cohort would determine whether these observations allow a definitive interpretation. However, a recent study has shown that in vitro gluten-stimulated IL-2 secretion correlates with the severity of patient symptoms (*79*), and our study provides important information on the cell populations and phenotypes that may contribute to this severity.

The analysis of TCR usage in gliadin-specific CD4^+^ T cells has been extensively characterized (*9*, *13*, *14*, *18*, *19*, *30*, *33*, *80*–*82*), resulting in the identification of the R motif in T cells responding to DQ2.5–glia-a2 and public TCR sequences observed between multiple individuals. In contrast to previous studies (*9*, *30*, *80*), we investigated core CDR3 motifs—enriched within epitope-specific populations independently of TRAV/J or TRBV/J biases—and identified critical residues predicted to be critical CDR3 contacts mediating specificity (*63*) and comprising up to ∼30% of the epitope-specific repertoires within multiple donors. The strong enrichment of the nongermline-encoded arginine residue within the CDR3β loop of *TRBV7-2/7-3^+^* TCRs in the DQ2.5–glia-α2–specific repertoire (*18*, *19*) has been shown to act as the lynchpin for docking of TCRs onto this epitope (*20*). Similarly, most core motifs identified here were associated with (but not encoded by) a V/(J) gene bias in the same TCR chain or, for the CDR3α motif in the DQ2.5–glia-α1a repertoire, the alternate chain, strongly suggesting that these independent V(J) gene elements work together with the CDR3 core motifs to impart specificity.

In addition to identifying core motifs that define individual antigen specificity, we identified a CDR3β W[D/E]….F motif that was enriched within, and predictive of, CD4^+^ T cell cross-reactivity to the highly homologous DQ2.5–glia-α1a (PFPQPELPY) and DQ2.5–glia-ω1 (PFPQPEQPF) epitopes. A related motif (CDR3β W.….F) was found to impart specificity only to the DQ2.5–glia-ω1 complex. Our structural analyses revealed that the additional D residue within WD motif act as critical molecular switch governing cross-reactivity to DQ2.5 restricted glia-α1a or glia-ω1 complexes. In G2 and the related XPA5 TCRs (*66*), the conserved D residue anchors to R^70^ of the DQ2.5 β chain, while the complementary hydrophobic residues (W113β in G2 and L109α in XPA5) stabilize the P3 to P5 region of the peptide, orienting R^70^ toward the peptide N terminus, thereby enabling dual recognition of both glia-α1a and glia-ω1. In contrast, the conserved D residue in the W316 TCR (*66*) engages R^70^ at P5 to P7 region, shifting R^70^ toward the C terminus and contacting P7-Q to favor glia-ω1 while excluding glia-α1a. Thus, the positioning of D residue, together with hydrophobic counterparts, dictates the orientation of R^70^ (DQ2.5 β chain) and determines whether TCRs achieve cross-reactivity or peptide selectivity. Moreover, we describe a “Gln” motif within the CDR3β loop that imparts specificity toward the Glia-ω2 determinant and thus provide a structural basis underpinning this TCR usage in CeD.

While a D/E motif has been reported from TCRs characterized from an HLA-DQ8 transgenic mouse and some human T cell clones (*83*), this bears limited relevance to DQ2.5-mediated CeD detailed here. Further, related work detailing the structural basis of HLA-DQ8–mediated CeD (*84*–*86*) showed that all TCRs sat atop the HLA-DQ8 Ag-binding cleft in a standard docking mode and hence was very remote from the site of the β57 polymorphism. Thus, unless an unprecedented mode of interaction of the D/E containing TCRs reported in interacting with the base of the Ag-binding cleft where the β57 polymorphic site resides (*83*), the mechanistic basis of this D/E motif remains unclear.

However, our findings, which includes a prominent hydrophobic (W) residue within the CDR3β motif directed toward DQ2.5–glia-α1a and DQ2.5–glia-ω1, does resonate somewhat with previous studies in a mouse model of type I diabetes, where hydrophobic residues within the CDR3β loops engendered self-reactivity toward I-Ag7 (*87*, *88*).

While the W[D/E] motif was detected and enriched within DQ2.5–glia-α1a–specific cells in our scRNA-seq experiment, these cells showed subthreshold binding of the DQ2.5–glia-ω1 dextramer and thus were not originally classified as cross-reactive. We found that of the total CD4^+^ T cells that bound either DQ2.5–glia-α1a– or DQ2.5–glia-ω1–dextramers, an average of only 15% (9.6 to 30.2% across the donors) were cross-reactive by dual dextramer staining. A previous study with HLA tetramers found this frequency to be even lower at ∼6% (0 to 20%) of all DQ2.5–glia-α1a– and DQ2.5–glia-ω1a–specific T cells (*14*). We propose that the competitive nature of the dextramer binding—combined with the fact that the affinity of W[D/E]-containing TCRs for DQ2.5–glia-α1a was substantially higher than for DQ2.5–glia-ω1—likely results in an underestimation of T cells that are cross reactive for DQ2.5–glia-α1a– or DQ2.5–glia-ω1a complexes. This should be noted as a possible limitation of combinatorial tetramer/dextramer-based approaches in the identification of cross-reactive T cells (*89*, *90*).

Previous studies have identified public gliadin antigen-specific CDR3 sequences across multiple patients with CeD (*9*, *30*). The core antigen-specific motifs we identified could also be detected in these public CDR3 sequences, supporting our observation that these motifs are reproducibly used in TCR responses to these epitopes. Moreover, we showed the structural importance of these core motifs and thus their predictive capacity, by accurately predicting TCR specificity from a database of 2129 clonotypes isolated using a pooled cocktail of tetramers for all four immunodominant wheat epitopes, across 50 patients with CeD (*30*). Collectively, we propose that these motifs provide a predictive capacity that may potentially form the basis of standardized biomarkers (*91*) or diagnostics of CeD, or in the identification of dominant antigenic targets, just as public TCRβ sequences generated from peripheral blood have been successfully used to predict cytomegalovirus serostatus (*16*). Moreover, the identification of motifs associated with epitope cross-reactivity may simplify therapy formulations or immune monitoring by narrowing the epitope panel required for screening.

In conclusion, this study provides a detailed analysis of the transcriptional and TCR repertoire landscape of CD4^+^ T cells specific for the four immunodominant DQ2.5-restricted gliadin epitopes in CeD, revealing functionally distinct subsets of activated T cells. Despite transcriptional heterogeneity, we found no evidence that distinct gliadin epitope specificities drive functional specialization among CD4^+^ T cells. Our definition of core CDR3 motifs that underpin epitope specificity and cross-reactivity deepen our understanding of gliadin-specific T cell repertoires and their specificity constraints and offer a foundation for diagnostic and therapeutic advances in CeD.

## MATERIALS AND METHODS

### Human samples

Human experimental work was conducted according to the Australian National Health and Medical Research Council (NHMRC) Code of Practice. Patients with celiac disease were recruited after provision of informed consent (Human Research Ethics Committees: Royal Melbourne Hospital ID: 2003.009; the Walter and Eliza Hall Institute of Medical Research ID: 03/04). All participants gave informed written consent.

All CeD donors were HLA-DQ2.5^+^ (either heterozygous or homozygous for DQA1*05 and DQB1*02). CeD was diagnosed on the basis of evidence of past duodenal histology showing characteristic villous atrophy in conjunction with positive CeD serology. Before gluten challenge, volunteers had followed a strict gluten-free diet for at least the previous 3 months and were confirmed to have normal levels of serum anti-tissue transglutaminase IgA and anti-deamidated gliadin peptide IgG (INOVA Diagnostics) (table S1). Gluten challenge consisted of four slices of white bread daily cut to toasting thickness (Bakers Delight, Victoria) for 3 days in a row, delivering an approximate daily dose of 12 g of wheat gluten. Blood was drawn at baseline (to measure serum antibody levels) and 6 days after commencing gluten challenge (day 6) where gluten ingestion started on day 1. Volunteers completed symptom diaries, recording symptom type and severity (6-point scale for donors 0572 and 0648 and 10-point scale for donors 1314 and 1621) daily until day 6 (table S2). Healthy controls were not included in the study as it has previously been shown that HLA-DQ2.5^+^ individuals without CeD do not have gluten-specific CD4^+^ memory T cells detectable with tetramers (*8*, *34*).

PBMC were isolated from whole blood using Ficoll-Paque Plus density-gradient centrifugation (GE Healthcare) and cryopreserved. PBMC were used in dextramer-based magnetic enrichment (donors CD0572, CD0648, CD1314, and CD1621) or tetramer-based magnetic enrichment (donors CD0572 and CD1597).

### Dextramer generation and dextramer-based magnetic enrichment of HLA-DQ2.5–gliadin–specific T cells

Peptides to HLA-DQ2.5 (HLA-DQA1*05:01, HLA-DQB1*02:01)–restricted gluten-derived T cell epitopes glia-α1a (QPFPQPELPYP), glia-α2 (FPQPELPYPQP), glia-ω1 (QPFPQPEQPFP), and glia-ω2 (PQPEQPFPWQ) were synthesized by GL Biochem (China), and the integrity of the peptides was verified by reverse-phase high-performance liquid chromatography and mass spectrometry. pHLA tetramers were generated by stepwise addition of phycoerythrin (PE)–conjugated steptavidin (BD Biosciences, #554061) to biotinylated peptide-HLA-DQ monomers (DQ2.5–glia-α1a or glia-ω1) at a 1:4 molar ratio in phosphate-buffered saline (PBS).

pHLA dextramers were prepared by stepwise addition of PE-conjugated DNA-barcoded dextramers (dCODE Klickmer; Immudex, dCXC-PfBC0491, dCXC-PfBC0492, dCXC-PfBC0493, and dCXC-PfBC0494) to biotinylated peptide-HLA-DQ monomers (DQ2.5–glia-α1a, DQ2.5–glia-α2, DQ2.5–glia-ω1, or DQ2.5–glia-ω2) at a 1:15 molar ratio in PBS supplemented with 1% (w/v) BSA and maintained at 4°C in the dark until use. The dextramer cocktail was prepared immediately before staining and contained 6 μl of each DNA-barcoded MHC dextramer-PE (160 nM) and 0.6 μl d-biotin (100 μM in PBS) per dextramer premixed to block free binding sites.

CeD donor PBMCs were thawed and rested overnight in complete RPMI (cRPMI) containing RPMI1640 (Thermo Fisher Scientific, #21870), 10% fetal bovine serum (FBS), Hepes, l-glutamine, and penicillin-streptomycin at 37°C with 5% CO_2_. Cells were counted and 53 million (CD0572), 57 million (CD0648), 27 million (CD1314), and 39 million (CD1621) PBMC were resuspended in PBS containing 2% FBS with the addition of 25% anti-human FcR block (Miltenyi Biotec) and treated with 50 nM dasatinib (Cell Signaling Technology) for 30 min at 37°C and then stained with the dextramer cocktail (5 nM final concentration for each dextramer) for 30 min at room temperature (RT). Cells were washed and labeled with anti-PE conjugated magnetic microbeads (Miltenyi Biotec), and dextramer-bound cells were enriched over a magnetic LS column (Miltenyi Biotec). Enriched cells were then stained at 4°C for 30 min in the dark with a cocktail of fluorescently labeled surface antibodies (AF700 anti-human CD14, AF700 anti-human CD19, BV480 anti-human CD3, and BUV395 anti-human CD4 in Brilliant Stain buffer), TotalSeq-C antibodies (anti-human CD14, CD183, CD185, CD194, CD196 CD197, CD25 CD27, and CD278), CD279, CD366, CD38, CD4, CD45RA, CD45RO, HLA-DR, TIGIT, and CD95 plus mouse immunoglobulin G1 (IgG1) κ and mouse IgG2a κ isotype controls and one of TotalSeq-C anti-human hashtag antibody 1-4 (table S12), followed by the addition of LIVE/DEAD Fixable Near-IR dead cell stain (Thermo Fisher Scientific) for 10 min at RT in the dark. Cells were washed three times with PBS containing 2% FBS, and single, live, CD14^−^CD19^−^CD3^+^CD4^+^dextramer^+^ cells from each donor were sorted into PBS containing 20% FBS using a FACSAria III cell sorter with FACSDiva software v8.0.1 (BD Immunocytometry Systems). Cells were pooled and resuspended in PBS containing 0.04% bovine serum albumin and immediately loaded into a 10x Chromium reaction. 10x Genomics Chromium Next GEM Single-Cell 5′ kit v.1.1, Chromium Single Cell V(D)J enrichment kit (Human T Cell) and Chromium Single Cell 5′ Feature Barcode Library Kit were used to generate GEX, VDJ, and Cite-seq libraries according to the manufacturer’s protocol. The cDNA libraries were sequenced on a MGITech MGISEQ2000RS platform using MGIEase V2 chemistry, targeting a median sequencing depth of 50,000, 5000, and 10,000 read pairs per cell for gene expression, TCR, and combined dCODE and TotalSeq-C libraries, respectively.

### Processing of 10x Genomics multiomic data

The multiplexed FASTQ files were processed using the Cellranger Multi command (v.6.0.0) against the GRCh38 human reference (refdata-cellranger-GRCh38-2020-A) for the gene expression data and the 10x VDJ GRCh38 human reference (refdata-cellranger-vdj-GRCh38-alt-ensembl-5.0.0) for the VDJ data. Cell Ranger Multi was configured to run with gene expression data (GEX), VDJ data (VDJ), and Antibody Capture. The feature file for the Antibody Capture contained three sets of features, the CSP barcodes, the HTO barcodes and the dextramer (DEX) barcodes as these were all contained in one fastq file. Paired clonotype calling and additional quality control were performed using the make_10x_clones_file function from CoNGA software package (v.0.1.2).

### Single-cell GEX and cell surface library analysis

The analysis of single-cell GEX and cell surface libraries was performed using Seurat (v.5.1.0) using R language (v.4.4.2). The quality control (QC) to filter out presumably lysed/dead cells and doublets was performed by removing cells with greater than 12% mitochondrial content and expressing fewer than 500 or greater than 4000 genes. After QC, the counts were log-normalized before feature selection, scaling, and PCA. The FindNeighbors, FindClusters, and RunUMAP tools were applied on the PCA embedding space to generate a two-dimensional (2D) projection and neighbor graph of GEX space. TCR clonotype information (described above) was then mapped back to cells in the object by matching their corresponding cellular barcodes. Counts for TotalSeq-C, HTOs, and peptide-MHC dextramer labels were center log ratio normalized. MULTIseqDemux (*92*) was used for demultiplexing HTOs.

Publicly available data from 10x Genomics derived from PBMCs of two healthy individuals (www.10xgenomics.com/datasets/pbm-cs-of-a-healthy-donor-5-gene-expression-and-cell-surface-protein-1-standard-3-0-0 and www.10xgenomics.com/datasets/human-pbmc-from-a-healthy-donor-10-k-cells-v-2-2-standard-5-0-0) were integrated with the gliadin-specific T cell dataset above. HLA typing of these samples using the 10x Genomics libraries revealed that Healthy PBMC_2 was HLA-DQ2.5 positive (heterozygous), while Healthy PBMC_1 was DQ2.5 negative. QC for these samples was performed identically as described above. The CD4^+^ T cells were subset from the individual samples before integration. The counts of each sample were normalized using SCTransform (v2) and Gamma-Poisson linear model. Harmony (v.1.2.1) (*93*) was applied to the PCA embedding space of the merged objects, treating each sample as a batch variable, thereby correcting for chemistry- and processing-associated effects while preserving biological variation. The FindNeighbors, FindClusters, and RunUMAP tools were applied to the Harmony embedding space to generate the integrated 2D projection and neighbor graph of GEX space.

### Donor, cell surface protein, and epitope assignment using feature barcodes

Cells were uniquely labeled with a combination of DNA-barcoded hashing to distinguish subjects, cell surface protein antibodies (BioLegend TotalSeq-C), and a set of DNA-barcoded MHC multimers (Immudex dCODE). Cells matching to each donor were distinguished by TotalSeq-C hashtag labels and demultiplexed using MULTIseqDemux. The assignment of epitope specificity was determined using a dextramer UMI count cutoff of 8 for each dextramer. Where two dextramers passed the UMI count threshold of 8, the cell was considered specific to both. In addition, to correct erroneous or missing dextramer assignments for individual cells within expanded clones, we assigned epitope specificity based on the majority of cells from a clone. Clonotypes with unexpected cross-reactivity were further investigated. Thirty nine unique clonotypes with apparent specificities for all four dextramers were excluded from the dataset as TCR transfectants expressing a selection of these TCR did not bind to tetramers of any of the four epitopes. A further five unique clonotypes with apparent specificities to three dextramers were also excluded. If the most abundant dextramer barcode for each cell was ≤8 UMIs, then no epitope specificity was assigned and the clonotype was excluded from the dataset.

### TCR repertoire analysis

T cell clones were defined as groups of cells with identical nucleotide sequences of both CDR3α and CDR3β. To correct erroneous or missing dextramer assignments at the cell level, epitope specificities were assigned to the level of clonotype where all cells with a shared TCR sequence were assigned on the basis of the majority of assignments within the clonotype. To measure the distance between TCR α/β clonotypes and plot logos for dominant motifs, we used the TCRdist algorithm implementation and plotting functions from tcr-dist python package (*63*). TCR clumping analysis and visualization was performed using the CoNGA package (v.0.1.2) (*26*).

### Tetramer-based magnetic enrichment and analysis of epitope specific CD4^+^ T cells in humans

Cryopreserved PBMC collected 6 days after wheat challenge from two patients with DQA1*05:01/DQB1*02:01^+^ celiac disease (donors CD0572 and CD1597; table S1) were thawed and rested overnight in cRPMI at 37°C with 5% CO_2_. Cells were counted and 8.6 million (CD0572) or 11.8 million (CD1597) PBMC were resuspended in PBS containing 2% FBS with the addition of 25% anti-human FcR block (Miltenyi Biotec) and treated with 50 nM dasatinib (Cell Signaling Technology) for 30 min at 37°C before the addition of HLA-DQ2.5–glia-α1a–PE or HLA-DQ2.5–glia-ω1–PE tetramers (each at 10 μg/ml final concentration) for 1 hour at RT. Cells were washed and labeled with anti-PE–conjugated magnetic microbeads, and tetramer-bound cells were enriched over a magnetic LS column (Miltenyi Biotec). Enriched cells were then stained at 4°C for 30 min in the dark with a cocktail of fluorescently labeled surface antibodies (AF700 mouse anti-human CD14, AF700 mouse anti-human CD19, BV480 mouse anti-human CD3, and BUV395 mouse anti-human CD4 in Brilliant Stain buffer; table S12) followed by FVS700 fixable viability stain (BD Biosciences) for 10 min at RT in the dark. Cells were washed and the entire samples were acquired on a FACSAria III cell sorter with FACSDiva 8.0.1 software (BD Immunocytometry Systems), and live CD19^−^CD14^−^ CD3^+^ CD4^+^ tetramer binding cells were single-cell index sorted into 96-well polymerase chain reaction (PCR) plates (Eppendorf #00301286) and stored at −80°C until use.

### Analysis of epitope-specific T cell repertoires

For plates containing sorted DQ2.5–glia-α1a– or DQ2.5–glia-ω1–specific CD4^+^ T cells, mRNA was reverse-transcribed in 2.5 μl using the SuperScript VILO cDNA Synthesis Kit (Thermo Fisher Scientific, #11754) (containing 1× VILO reaction mix, 1× SuperScript enzyme mix, and 0.1% Triton X-100) and incubated at 25°C for 10 min, 42°C for 120 min and 85°C for 5 min. The entire volume was then used in a 25-μl first-round PCR reaction with 1.5 U Taq DNA polymerase (QIAGEN, #201207), 1× PCR buffer, 1.5 mM MgCl_2_, 0.25 mM deoxynucleoside triphosphates, and a mix of 40 human TRAV external sense primers and a human TRAC external antisense primer, along with 28 human TRBV external sense primers and a human TRBC external antisense primer (*65*, *94*) (each at 5 pmol/μl), using standard PCR conditions. For the second-round nested PCR, a 2.5-μl aliquot of the first-round PCR product was used in separate TRAV- and TRBV-specific PCRs using the same reaction mix described above; however, a set of 40 human TRAV internal sense primers and a human TRAC internal antisense primer or a set of 28 human TRBV internal sense primers and a human TRBV internal antisense primer were used (*65*, *94*). Second-round PCR products were visualized on a gel, and positive reactions were purified with ExoSAP-IT reagent (Thermo Fisher Scientific, #78201). Purified products were used as template in sequencing reactions with human internal TRAC or TRBC antisense primers and sequenced on an ABI 3730 DNA Sequencer at the Monash Micromon Genomics Facility (Monash University), and *TRBV* and *TRAV* gene usage was determined using IMGT/V-QUEST (*95*).

### In vitro TCR expression

Selected P2A-linked TCRαβ gene constructs and full-length TCRα and TCRβ chains were custom ordered from GenScript and cloned, respectively, into pMIGII vector (*96*) (RRID: Addgene_52107; a gift from D.A.A. Vignali) or pLV-EF1α-MCS-IRES vector (Biosettia, #cDNA-pLV). All constructs were sequenced to confirm the correct TCR sequence. Human embryonic kidney (HEK) 293T cells (American Type Culture Collection, #CRL-3216) were maintained in complete Dulbecco’s modified Eagle’s medium (DMEM) (cDMEM) containing DMEM (Thermo Fisher Scientific, #11960), 10% fetal bovine serum (FBS), Hepes, l-glutamine, and penicillin-streptomycin in a humidified incubator at 37°C and 10% CO_2_. HEK293T cells were plated at 3.5 × 10^5^ cells per well of a six-well plate in 3.5 ml of cDMEM. The following day, 7 μl of Lipofectamine 2000 (Thermo Fisher Scientific, #11668) was added to 143 μl of OptiMEM (Thermo Fisher Scientific, #31985) in an Eppendorf tube and incubated for 10 min at RT. The Lipofectamine/OptiMEM mixture was then added dropwise to either (i) 700 ng of pMIGII encoding an αβTCR sequence and 700 ng of pMIGII encoding CD3γδε and ζ subunits (*97*) or (ii) 420 ng of each of pLV-TCRα, pLV-TCRβ, and pLV encoding CD3γδε and ζ subunits (*98*) and incubated for a further 15 min at RT. The Lipofectamine-OptiMEM-DNA mixture was then added dropwise to a well(s) of 293T cells in the six-well plate and swirled to mix gently before returning to the incubator. After 48 hours, the culture medium was aspirated, and cells were detached from the plate by repeated washing with fluorescence-activated cell sorting (FACS) buffer (PBS + 0.1% bovine serum albumin). Transfected HEK293T cells were labelled with individual HLA-DQ2.5-gliadin tetramers for 1 hour at RT, followed by allophycocyanin-conjugated anti-human CD3ε antibody (BioLegend, #300412) and Live/Dead Aqua Blue viability stain (Thermo Fisher Scientific, #L34966). Cells were analyzed on a BD LSRFortessa X20 with FACSDiva software v8.0.1 (BD Immunocytometry Systems). Collected data were analyzed using Flowjo v10.9.0 (FlowJo).

### Protein expression and purification

HLA-DQ2.5–glia-α1a/ω1–restricted αβTCRs were engineered with the extracellular domain of human *TRBC* constant region and the *TRAV*/*TRBV* variable domains, incorporating an engineered disulfide bond within the constant regions, as described previously (*99*). TCRα and TCRβ chains were expressed separately in *Escherichia coli* BL21 (DE3). Inclusion bodies were purified and refolded in buffer containing 100 mM tris (pH 8.0), 5 M urea, 0.4 M l-arginine, 2 mM EDTA, 0.2 mM phenylmethylsulfonyl fluoride, 0.5 mM oxidized glutathione, and 5 mM reduced glutathione for 72 hours, at 4°C with continuous stirring. The samples were dialyzed extensively against 10 mM tris (pH 8.0) and purified by a four-step chromatographic procedure: DEAE anion exchange (Cytiva), size exclusion (Superdex 200, 16/600; Cytiva), hydrophobic interaction (Hi Trap SP HP; Cytiva), and anion exchange (HiTrap Q HP column; Cytiva) chromatography.

The extracellular domains of HLA-DQ2.5 (HLA-DQA*05:01 and HLA-DQB1*02:01) and HLA-DQ8 (HLA-DQA*03:01 and HLA-DQB1*03:02) were covalently linked to gliadin epitopes at the N terminus of DQ β chain, followed by an enterokinase cleavage site, fos and jun leucine-zipper domains, a BirA biotinylation site, and a polyHistidine tag at the C terminus. To enhance the stability of DQ2.5-gliadin complex, a covalent linkage was introduced between position 74 of the HLA-DQ α chain and P11 of the gliadin epitopes. HLA-DQ proteins were expressed in a baculovirus-insect cell system using High Five cells (*Trichoplusia ni* BTI-TN-5B1-4; Thermo Fisher Scientific) (*100*). After 72 hours of baculovirus infection, soluble HLA-DQ proteins were harvested from the culture supernatant, concentrated, and buffer exchanged [10 mM tris (pH 8.0) and 500 mM NaCl] using tangential flow filtration (Cogent M1 TFF system, Merck Millipore). Proteins were purified by immobilized metal affinity chromatography (Nickel-Sepharose 6 Fast Flow; Cytiva) and size exclusion chromatography (Superdex 200, 16/600; Cytiva).

Purified monomeric peptide-HLA-DQ complexes were biotinylated using BirA biotin protein ligase) in buffer containing 50 mM bicine (pH 8.3), 10 mM adenosine 5′-triphosphate, 10 mM MgOAc, 50 mM d-biotin, and 2.5 mg BirA. BirA was prepared according to published protocol (*101*).

### Surface plasmon resonance

Affinity measurements were performed by SPR on a Biacore T200 instrument (Cytiva). Biotinylated HLA-DQ2.5 molecules were immobilized on a streptavidin sensor chip (Cytiva) to a level of ∼3000 response units (RUs). Biotinylated HLA-DQ8–glia-α1 was used as a reference surface to control for nonspecific binding. Serial dilutions (200 to 0 μM) of TCR were injected over the flow cell surface in buffer containing 20 mM Hepes (pH 7.5), 150 mM NaCl, 1 mM EDTA, and 0.005% Tween 20 at a flow rate of 10 μl/min. Sensorgram and dose-response curves were fitted using a one site–specific binding model and analyzed with GraphPad Prism v.10.6.0 (GraphPad Software). Three independent experiments (*n* = 3) were performed for each TCR. Equilibrium response values were normalized against the calculated maximum response before combining datasets. Data are shown as means ± SEM. RA2.7 TCR (*102*) and G9 TCR (*65*) served as negative and positive control, respectively.

### Crystallization, data collection, and processing

Monomeric HLA-DQ2.5-gliadin peptide proteins were pre-treated with enterokinase (New England Biolabs) to remove C-terminal tag before complex formation with G2 TCR for crystallization. G2 TCR-HLA-DQ2.5-peptide ternary complexes were concentrated up to ∼10 mg/ml and subjected to high-throughput crystallization screening at the Monash Molecular Crystallisation Platform using an automated NT8 robotic system. Inititial crystal hits were optimized using hanging-drop vapor diffusion method in 24-well plates, mixing protein and mother liquor at a 1:1 ratio and equilibrated against 500 μl of mother liquor. The G2 TCR ternary complex crystals were obtained in condition containing 0.2 M trisodium citrate, and 16 to 20% (w/v) PEG-3350 (polyethylene glycol, molecular weight 3350). The C82 TCR ternary complex crystal was obtained in condition containing 16 to 20% PEG-3350, 0.1 M 2-morpholinoethanesulphonic acid (pH 6.5), and 0.2 M potassium sodium tartrate solution. Crystals were cryoprotected in mother liquor supplemented with 20% glycerol and flash-frozen in liquid nitrogen. Diffraction data were collected on the MX2 Beamline at the Australian Synchrotron using an Eiger X 16M detector. Data were processed and scaled using XDS and the CCP4 Software Suite version 9.0.

### Structure determination, refinement, and validation

G2 TCR-HLA-DQ2.5-peptide crystal structures were solved by molecular replacement using PHASER (CCP4 Software Suite, version 9.0). Separate search models were used for the αβTCR [Protein Data Bank (PDB) ID: 6V1A (*103*)] and HLA-DQ2.5–glia-ω1 [PDB ID: 6MFF (*14*)]. Iterative cycles of model building were performed in Coot (version 0.9.8.96) (*104*), followed by automated refinement in PhenixRefine [PHENIX (*105*), version 1.21.2-5419]. The quality of the refined ternary structures was validated using PDB data validation and deposition services. G2 and C82 TCR variable domain was numbered according to the IMGT unique numbering system (*106*). Data processing and refinement statistics were summarized in table S6. BSA calculations were performed with Areaimol, and intermolecular contact analyses were conducted using Contacts (CCP4 Software Suite, version 9.0; table S7). Structural figures were generated with PyMOL version 2.4.0.
