# Supplementary material for: Gliadin-specific CD4+ T cells exhibit functional diversity independent of TCR usage or epitope specificity
Source: Sci Adv. 2026 Oct 2;12(40):eaei3175. doi: 10.1126/sciadv.aei3175 (PMC13632718; doi:10.1126/sciadv.aei3175)
Supplement: Supplementary file 1 [file sciadv.aei3175_sm.pdf]

Science Advances
AAAS

Supplementary Materials for

Gliadin-specific $CD4^+$ T cells exhibit functional diversity independent of TCR usage or epitope specificity

Claerwen M. Jones *et al.*

Corresponding author: Jamie Rossjohn, jamie.rossjohn@monash.edu;
Nicole L. La Gruta, nicole.la.gruta@monash.edu

Sci. Adv. **12**, eaei3175 (2026)
DOI: 10.1126/sciadv.aei3175

This PDF file includes:

Figs. S1 to S10
Tables S1 to S12

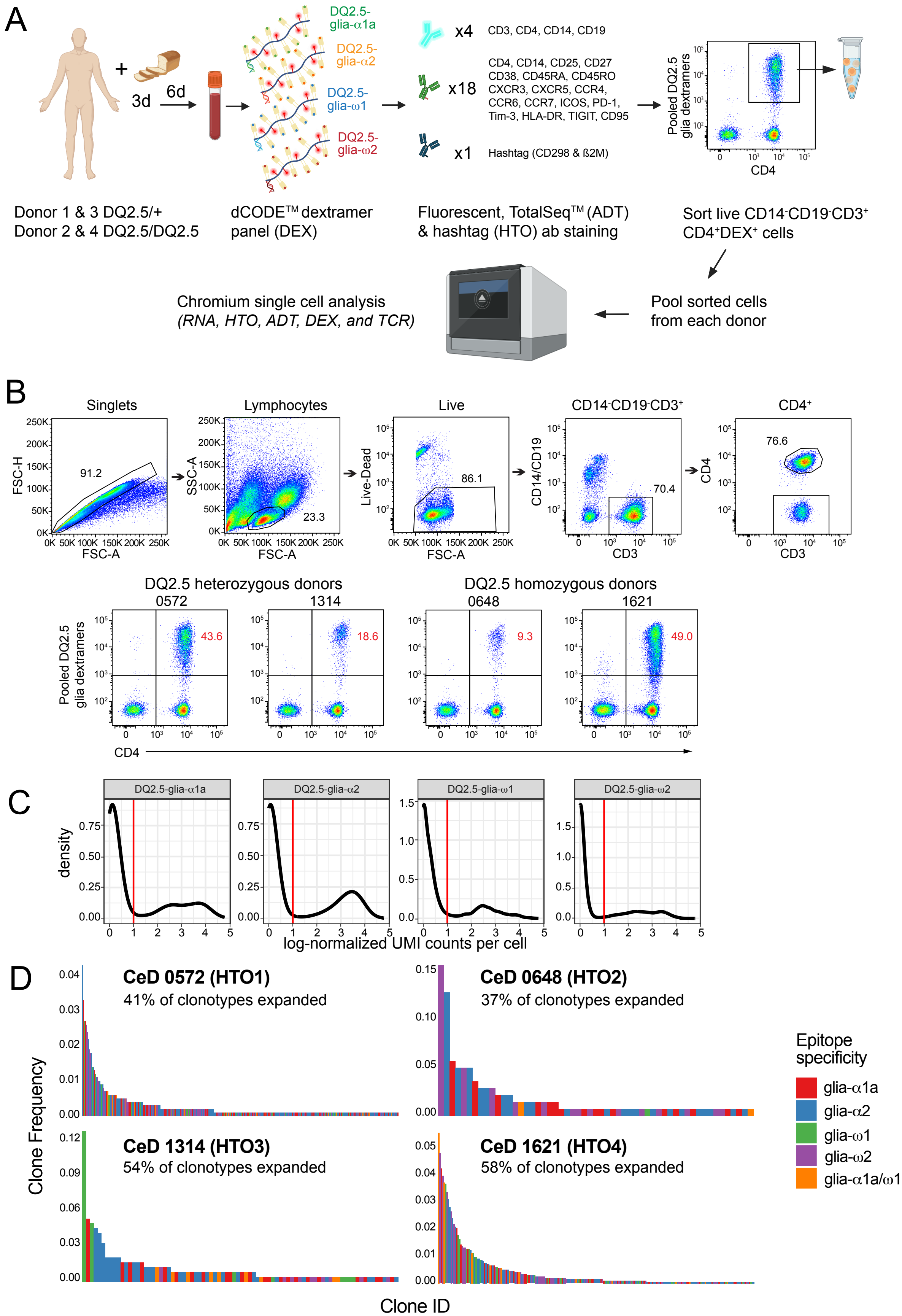

A
Donor 1 & 3 DQ2.5/+
Donor 2 & 4 DQ2.5/DQ2.5
3d
6d
DQ2.5-glia-α1a
DQ2.5-glia-α2
DQ2.5-glia-ω1
DQ2.5-glia-ω2
dCODE™ dextramer panel (DEX)
x4 CD3, CD4, CD14, CD19
x18 CD4, CD14, CD25, CD27 CD38, CD45RA, CD45RO CXCR3, CXCR5, CCR4, CCR6, CCR7, ICOS, PD-1, Tim-3, HLA-DR, TIGIT, CD95
x1 Hashtag (CD298 & ß2M)
Fluorescent, TotalSeq™ (ADT) & hashtag (HTO) ab staining
Pooled DQ2.5 glia dextramers
CD4
Sort live CD14⁻CD19⁻CD3⁺ CD4⁺DEX⁺ cells
Pool sorted cells from each donor
Chromium single cell analysis (RNA, HTO, ADT, DEX, and TCR)
B
Singlets
FSC-H
FSC-A
91.2
Lymphocytes
SSC-A
23.3
Live
Live-Dead
86.1
CD14⁻CD19⁻CD3⁺
CD14/CD19
CD3
70.4
CD4⁺
CD4
76.6
DQ2.5 heterozygous donors
0572
43.6
1314
18.6
DQ2.5 homozygous donors
0648
9.3
1621
49.0
Pooled DQ2.5 glia dextramers
CD4
C
DQ2.5-glia-α1a
DQ2.5-glia-α2
DQ2.5-glia-ω1
DQ2.5-glia-ω2
density
log-normalized UMI counts per cell
D
CeD 0572 (HTO1)
41% of clonotypes expanded
CeD 0648 (HTO2)
37% of clonotypes expanded
CeD 1314 (HTO3)
54% of clonotypes expanded
CeD 1621 (HTO4)
58% of clonotypes expanded
Clone Frequency
Clone ID
Epitope specificity
glia-α1a
glia-α2
glia-ω1
glia-ω2
glia-α1a/ω1

Fig. S1. Generating 10x scRNA seq database.

(**A**) Experimental workflow for single cell analysis of $CD4^+$ T cells from PBMC of four celiac disease patients. Patients (two heterozygous and two homozygous for HLA-DQ2.5) were bled 6d after wheat challenge and PBMC were stained with a panel of dCODE™ dextramers (DQ2.5-glia-α1a, -α2, -ω1, and -ω2). Dextramer-binding cells were magnetically enriched and stained with a panel of fluorescent and TotalSeq™ antibodies plus a TotalSeq™ hashtag antibody for donor identification. Live $CD14^-CD19^-CD3^+CD4^+DEX^+$ cells were sorted from each donor (example shown is from donor 0572), pooled, and single cell high throughput sequencing performed on antibody-derived tags (ADT), hashtag oligos (HTO), TCR, and gene expression (GEX). Created using BioRender. *La Gruta, N. (2025) https://BioRender.com/ajflqpz*.
(**B**) Gating strategy for sorting of single live $CD14^-CD19^-CD3^+CD4^+$dextramer$^+$ cells from celiac disease patients. The percentage of dextramer-binding $CD4^+$ T cells of total $CD4^+$ T cells is indicated in red type. (**C**) Bimodal distribution of the number of dextramer unique molecular identifiers (UMIs) on cells within the 10x scRNA seq dataset for each of the DQ2.5-glia-α1a, -α2, -ω1, and -ω2 dextramers, indicating the cut-offs for positive staining. (**D**) Distribution of clone sizes within individual patients HTO1-4 indicating epitope-specificity.

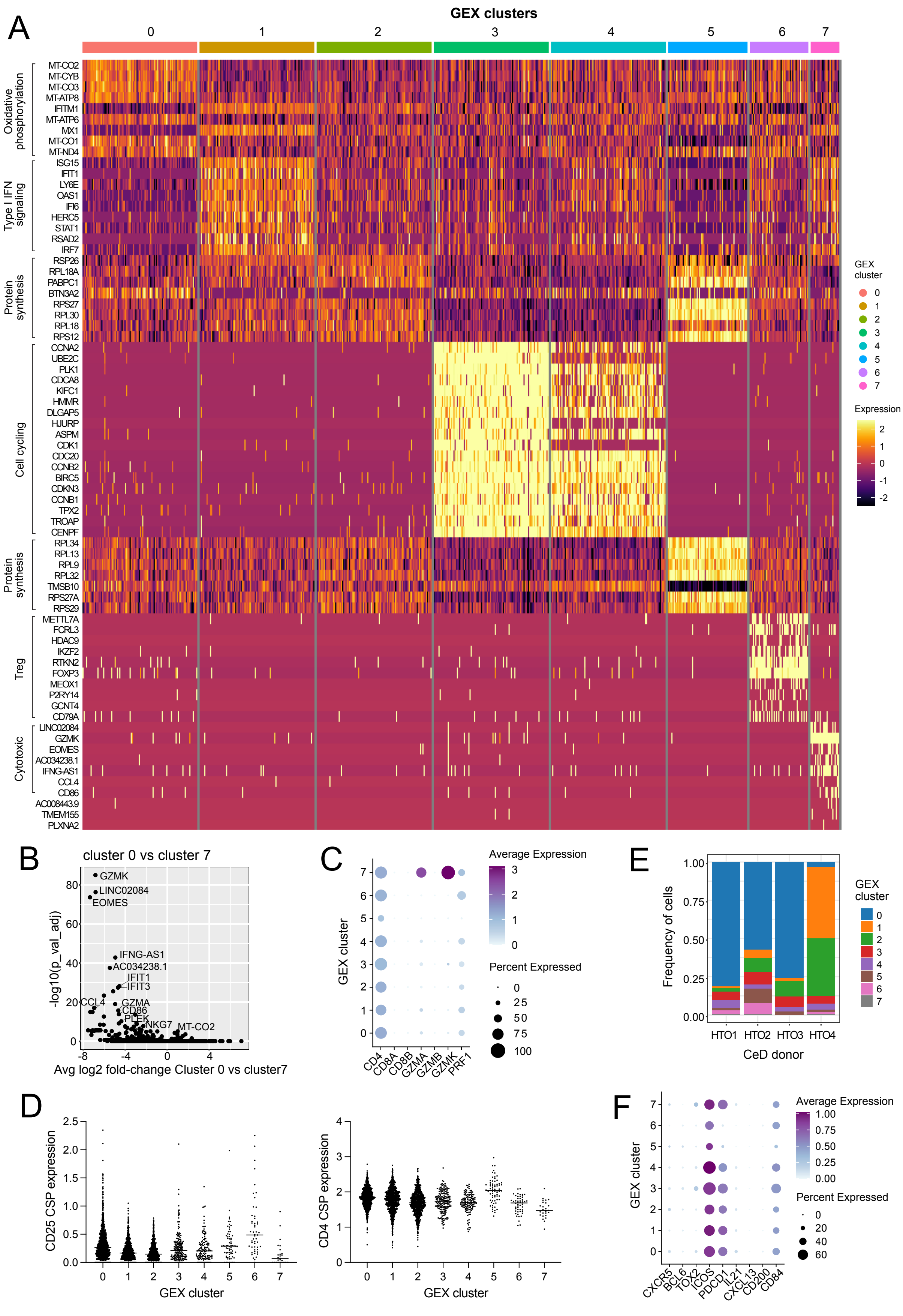
A
GEX clusters
0 1 2 3 4 5 6 7
Oxidative phosphorylation
MT-CO2
MT-CYB
MT-CO3
MT-ATP8
IFITM1
MT-ATP6
MX1
MT-CO1
MT-ND4
Type I IFN signaling
ISG15
IFIT1
LY6E
OAS1
IFI6
HERC5
STAT1
RSAD2
IRF7
Protein synthesis
RSP26
RPL18A
PABPC1
BTN3A2
RPS27
RPL30
RPL18
RPS12
Cell cycling
CCNA2
UBE2C
PLK1
CDCA8
KIFC1
HMMR
DLGAP5
HJURP
ASPM
CDK1
CDC20
CCNB2
BIRC5
CDKN3
CCNB1
TPX2
TROAP
CENPF
Protein synthesis
RPL34
RPL13
RPL9
RPL32
TMSB10
RPS27A
RPS29
Treg
METTL7A
FCRL3
HDAC9
IKZF2
RTKN2
FOXP3
MEOX1
P2RY14
GCNT4
CD79A
Cytotoxic
LINC02084
GZMK
EOMES
AC034238.1
IFNG-AS1
CCL4
CD86
AC008443.9
TMEM155
PLXNA2
GEX cluster
0 1 2 3 4 5 6 7
Expression
2 1 0 -1 -2
B
cluster 0 vs cluster 7
GZMK
LINC02084
EOMES
IFNG-AS1
AC034238.1
IFIT1
IFIT3
CCL4
GZMA
CD86
PLEK
NKG7
MT-CO2
-log10(p_val_adj)
0 20 40 60 80
-8 -4 0 4
Avg log2 fold-change Cluster 0 vs cluster7
C
GEX cluster
7 6 5 4 3 2 1 0
CD4 CD8A CD8B GZMA GZMB GZMK PRF1
Average Expression
3 2 1 0
Percent Expressed
0 25 50 75 100
D
CD25 CSP expression
0.0 0.5 1.0 1.5 2.0 2.5
GEX cluster
0 1 2 3 4 5 6 7
CD4 CSP expression
0 1 2 3 4
GEX cluster
0 1 2 3 4 5 6 7
E
Frequency of cells
0.00 0.25 0.50 0.75 1.00
HTO1 HTO2 HTO3 HTO4
CeD donor
GEX cluster
0 1 2 3 4 5 6 7
F
GEX cluster
7 6 5 4 3 2 1 0
CXCR5 BCL6 TOX2 ICOS PDCD1 IL21 CXCL13 CD200 CD84
Average Expression
1.00 0.75 0.50 0.25 0.00
Percent Expressed
0 20 40 60

G

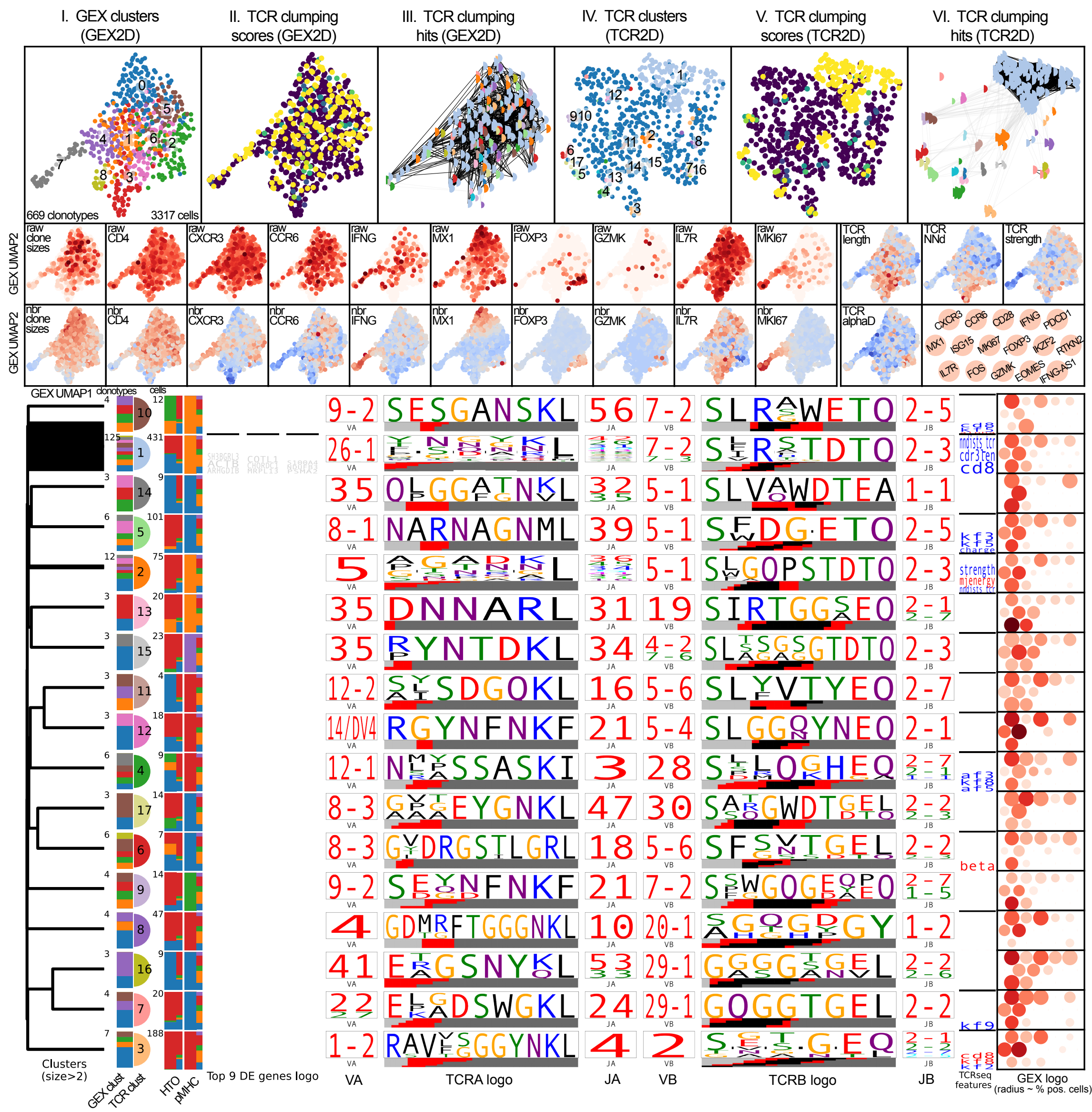

HTO key

Blue	HTO1
Orange	HTO2
Green	HTO3
Red	HTO4

pMHC key

Blue	Glia-α1a
Orange	Glia-α2
Green	Glia-ω1
Red	Glia-ω2
Purple	Glia-α1a/ω1

For each key
Left: frequency within cluster
Right: frequency within dataset

TCR sequence features (from Schattgen et al 2022) (*26*)

length	combined length of the CDR3α and CDRβ
alphaD	ordinal distance between V and J segments
NNdist	nearest-neighbour TCR similarity
strength	enrichment for strongly interacting amino acids in the CDR3
mjenergy	mean value of MJ statistical potential for each amino acid
beta	preference to appear in beta sheets
kf, af	Refers to Kidera and Atchley factors summarizing physiochemical properties of amino acids
cd8	cd8 vs cd4 preference score

Fig. S2. Characterstics of GEX clusters and correspondence with TCR clusters.

(**A**) Gene expression heat map of the Top10 differentially expressed genes (DEGs) defining GEX clusters 0-7 ranked by average log fold change. Scaled gene expression indicated by colour. (**B**) Volcano plots showing the relationships between -log10(adjusted p value) (y-axis) and log2(fold change) (x-axis) for genes differentially expressed by $CD4^{+}$ T cells in cluster 0 vs cluster 7. (**C**) Bubble plot showing *CD4, CD8A/B, GZMA/B/K* and *PRF1* gene expression in GEX clusters 0-7. Circle size shows the proportion of cells expressing the gene, colour shows the gene expression level (scale 0-3). (**D**) Cell surface protein (CSP) expression of CD25 (left) and CD4 (right), as measured by TotalSeq™-C antibody barcoding, on total gluten-specific $CD4^{+}$ T cells within GEX clusters 0-7. Horizontal lines indicate median values. The number of cells in each cluster is listed in Fig. 2A. (**E**) Distribution of cells from donors HTO1-4 in GEX clusters 0-7. (**F**) Bubble plot showing the expression of selected genes associated with follicular helper T cells across GEX clusters 0-7. Circle size shows the proportion of cells expressing the gene, colour shows the gene expression level (scale 0-1). (**G**) Application of CoNGA on the 10x scRNA seq database. At the top are six 2D UMAP projections of clonotypes in the dataset based on GEX similarity (top left three panels) and TCR similarity (top right three panels), coloured from left to right by (I) GEX cluster assignment (nine GEX clusters, 0-8); (II) TCR clumping score; (III) joint GEX:TCR cluster assignment for clonotypes with significant TCR clumping scores, using a bi-coloured disk whose left half indicates GEX cluster and whose right half indicates TCR cluster; (IV) TCR cluster (17 TCR clusters, 1-17); (V) TCR clumping score; and (VI) GEX:TCR cluster assignments for TCR clumping hits, as in III. Below are two rows of GEX landscape plots coloured by (first row, left) raw expression of selected marker genes; (second row, left) *Z*-score-normalized and GEX neighbourhood-averaged (nbr) expression of the same marker genes; and (both rows, right) TCR sequence features (see key under graph for TCR sequence feature descriptions). In the bottom section, GEX and TCR sequence features of TCR clumping hits in clusters with two or more hits are summarized by a series of logo-style visualizations. Each cluster is indicated by a multicoloured "disk" coloured according to TCR cluster (right half) and GEX cluster (left half). The two numbers above each disk show the number of hits within the cluster (on the left) and the total number of cells in those clonotypes (on the right). The dendrogram at the left shows similarity relationships among the clusters based on connections in the GEX and TCR neighbour graphs. For each cluster is shown, from left to right: donor identification (see HTO key below graph); epitope specificity (see pMHC key below graph); differentially expressed genes (DEGs; DEGs with fold-changes less than 2 are shown in gray); TCR sequence logos showing V and J gene usage and CDR3 sequences for the TCR alpha and beta chains; biased TCR sequence scores, with red indicating elevated scores and blue indicating decreased scores relative to the rest of the dataset; and GEX 'logos' for each cluster, consisting of a panel of marker genes shown with red disks coloured by mean expression and sized according to the fraction of cells expressing the gene (gene names are given above). DEG and TCR sequence logos are scaled by the adjusted *P* value of the associations, with full logo height requiring a top adjusted *P* value below 10^{-6}.

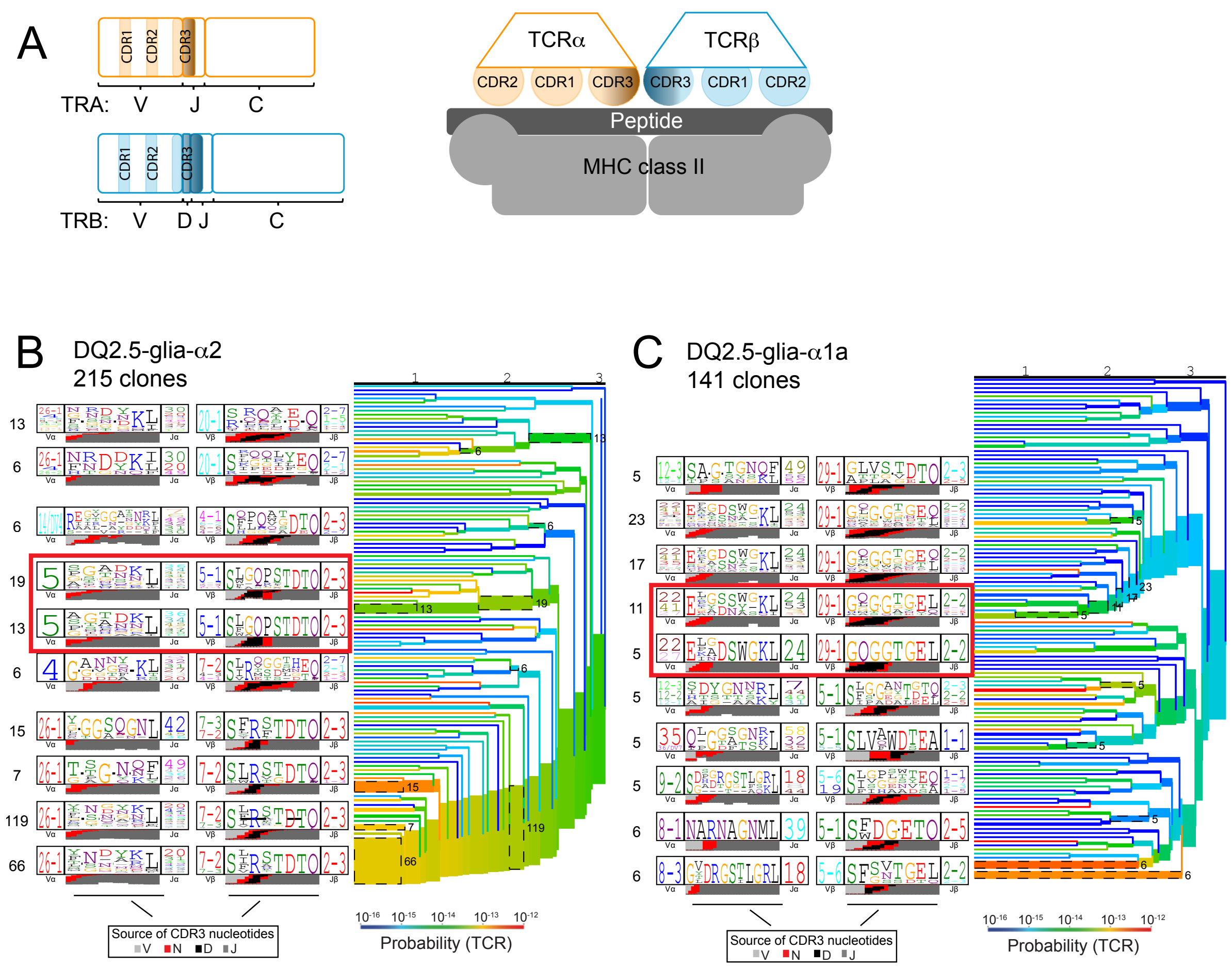

Fig. S3. Epitope reactivities and analysis of TCR usage bias and motifs contributing to specificity.

(**A**) Left: Schematic of variable (V), diversity (D), joining (J) and constant (C) segments in the TCR α- (TRA; orange) and β- (TRB; blue) chains. Also shown are the complementarity-determining regions (CDR) 1, 2 and 3 of the α- and β-chain chains. CDR1 and CDR2 are encoded by V gene segments in both chains while CDR3 encompasses the junction between V and J segments for the α-chain, and V, D and J segments for the β-chain. Non-templated nucleotide insertions and deletions occur at these junctions within the CDR3. Right: Schematic depicting typical interactions between TCR CDR loops and pMHC class II. (**B, C**) TCRdist clustering trees depicting the distance between TCRs within the (**B**) HLA-DQ2.5-glia-α2-specific TCRαβ repertoire (215 clones from 4 CeD patients HTO1-4) and (**C**) HLA-DQ2.5-glia-α1a-specific TCRαβ repertoire (141 clones from 4 CeD patients HTO1-4). Red boxes highlight the pairings referred to in the Results section text. The thickness of the branches is proportional to the number of TCRαβ clones represented by those branches. The TCR logo containing the CDR3 sequence is shown on the left with residue height scaled by frequency. The coloured bars underneath represent the source of the nucleotides that encode the logo, as depicted in key. The branches are coloured according to TCR generation probability as depicted in key.

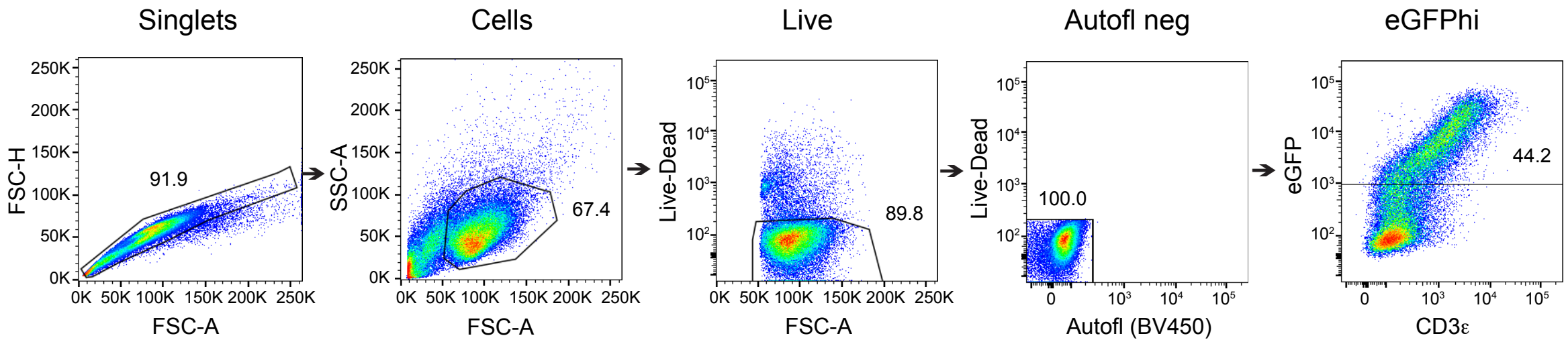

Fig. S4. Representative gating strategy for the detection of tetramer-binding on TCR-transfected 293T cells.

293T cells transiently co-transfected with selected TCR and CD3γδεζ were stained with anti-CD3ε antibody and individual tetramers. Tetramer-binding cells presented in Figs. 4I, 4J, 8B and fig. S10 are single live, eGFPhi cells. Numbers in black type indicate percentage of cells within a gate.

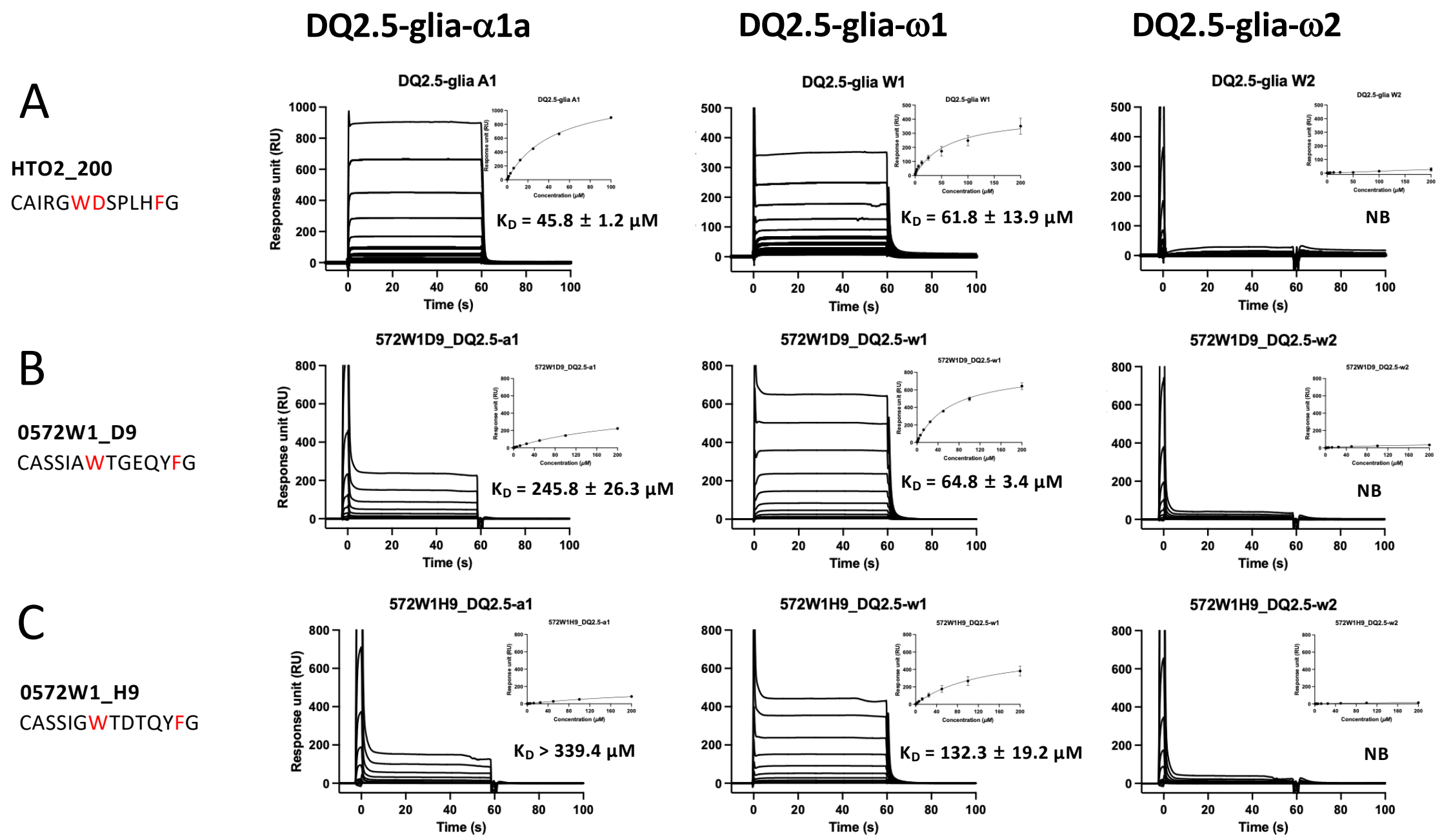

Fig. S5. Surface plasmon resonance binding affinity measurements.

Sensorgram and (inset) dose-response curve fit into one-site specific binding model of (**A**) HTO2_200, (**B**) 0572W1_D9, and (**C**) 0572W1_H9 TCR interaction with HLA-DQ2.5-glia-α1a (left), HLA-DQ2.5-glia-ω1 (middle) and HLA-DQ2.5-glia-ω2 (right). To control for nonspecific binding, HLA-DQ8-glia-α1 was immobilized in the reference flow cell. For K_D determination, all SPR data was derived from two independent experiments, in duplicate. Maximal concentration in the dilution series varied from 100 to 200 µM. Dose-response binding curves show mean ± s.d. from the experiments. K_D, dissociation constant; NB, no binding; ND, not determined; RU, response units. The TCR CDR3β amino acid sequence is shown below each TCR name with residues associated with CDR3β motifs described in Fig. 4F & G in red type.

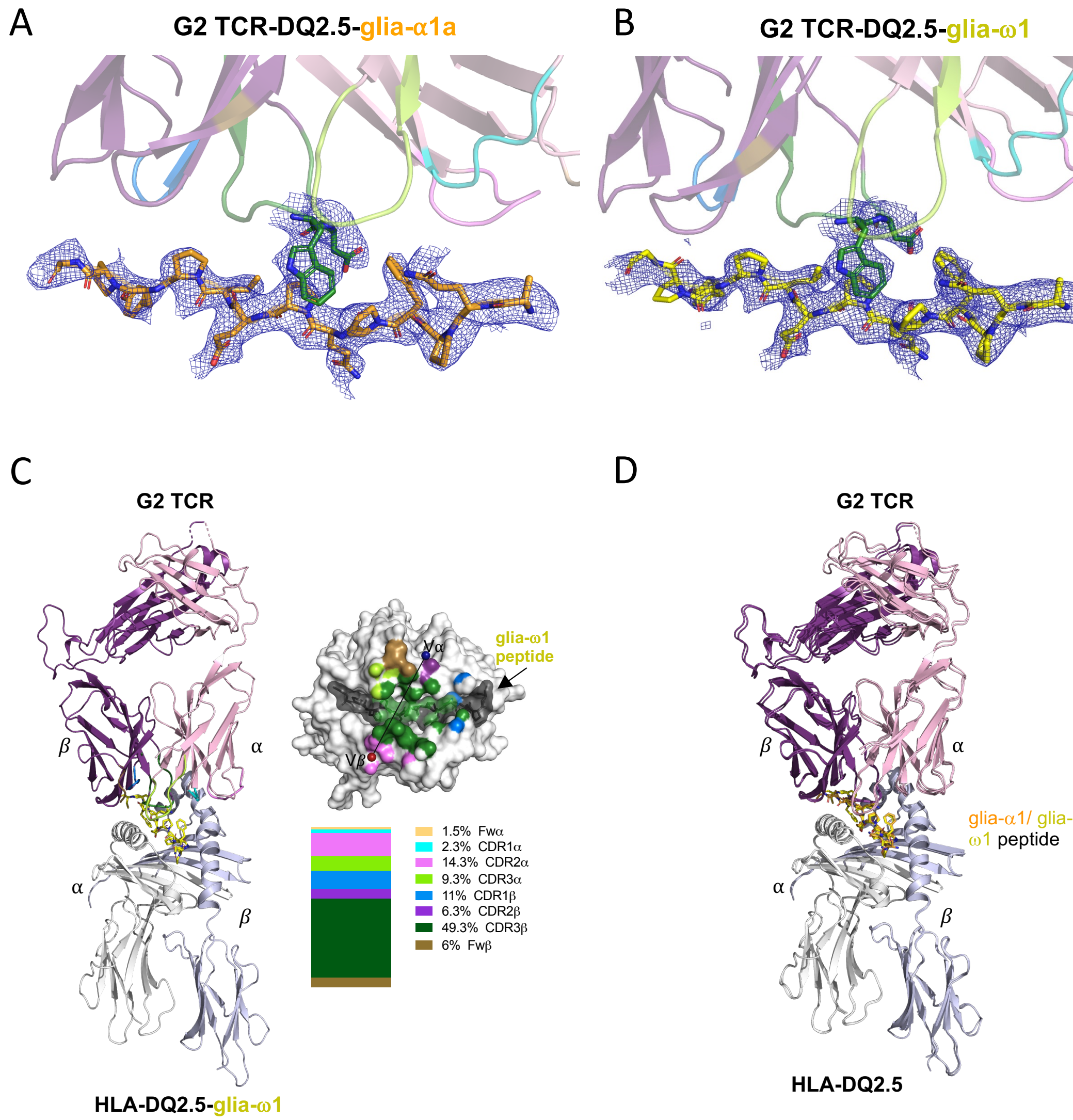

Fig. S6. Overall structure, TCR footprint and composite SA-omit map of the G2-TCR-DQ2.5-glia-α1a and G2-TCR-DQ2.5-glia-ω1 complex.

The σA-weighted 2Fo-Fc map (blue, contoured at 1.0σ) shows electron density for interface residues (peptide and ^{112}WD^{113} motif) for (**A**) G2-TCR-DQ2.5-glia-α1a complex and (**B**) G2-TCR-DQ2.5-glia-ω1 complex. The glia-α1a and glia-ω1 peptides are shown in orange and yellow sticks, respectively. CDR loops are highlighted as follows: CDR1α (cyan), CDR2α (violet), and CDR3α (light green); CDR1β (blue), CDR2β (purple), CDR3β (dark green). The FWβ residue is shown in brown. (**C**) Overall structure of the G2 TCR bound to DQ2.5-glia-ω1, with the TCR footprint coloured according to the nearest TCR contact residue. The centre of mass for Vα and Vβ position is indicated by red and blue spheres, respectively, connected via a black line. The HLA-DQ2.5 α- and β-chains are shown in white and light blue, respectively. The peptide, CDR loops and FWβ residues are coloured as in panel B. (**D**) Superimposed crystal structures of the G2 TCR bound to DQ2.5glia-α1a and DQ2.5glia-ω1 complexes.

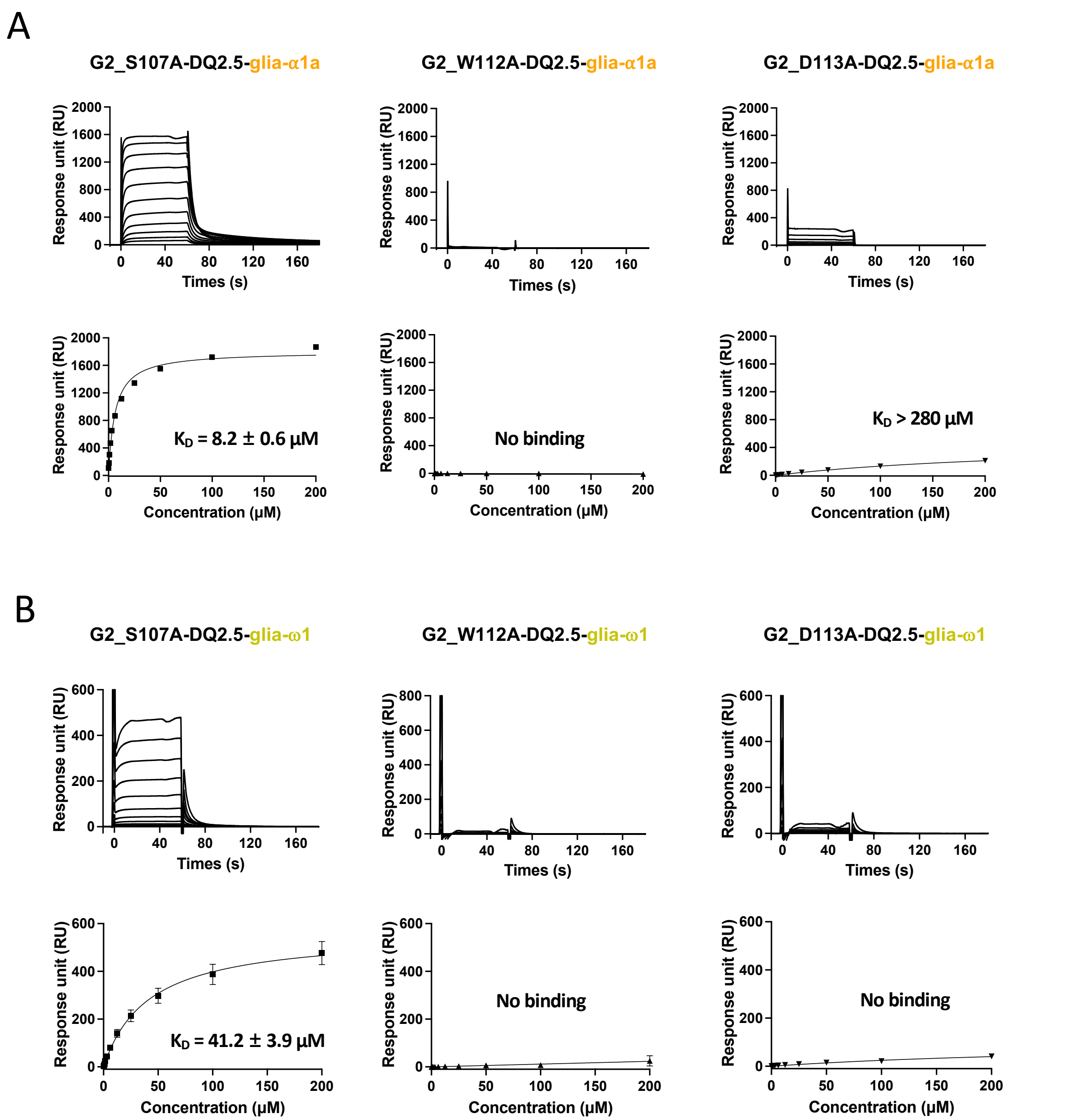

Fig. S7. Affinity analysis of G2 TCR point mutations for DQ2.5-glia-α1a and DQ2.5-glia-ω1.

Binding analysis of G2-TCR mutants toward (**A**) DQ2.5-glia-α1a and (**B**) DQ2.5-glia-ω1 were assessed by SPR. Data were derived from three independent experiments, with equilibrium constant (K_D) calculated using a single ligand binding model. HLA-DQ8-glia-α1 served as reference flow cell to control for nonspecific binding. Equilibrium response curves were normalised to the theoretical maximum response, and measurements were combined. Data points represent mean values from replicate experiments, with error bars indicating ± s.e.m.

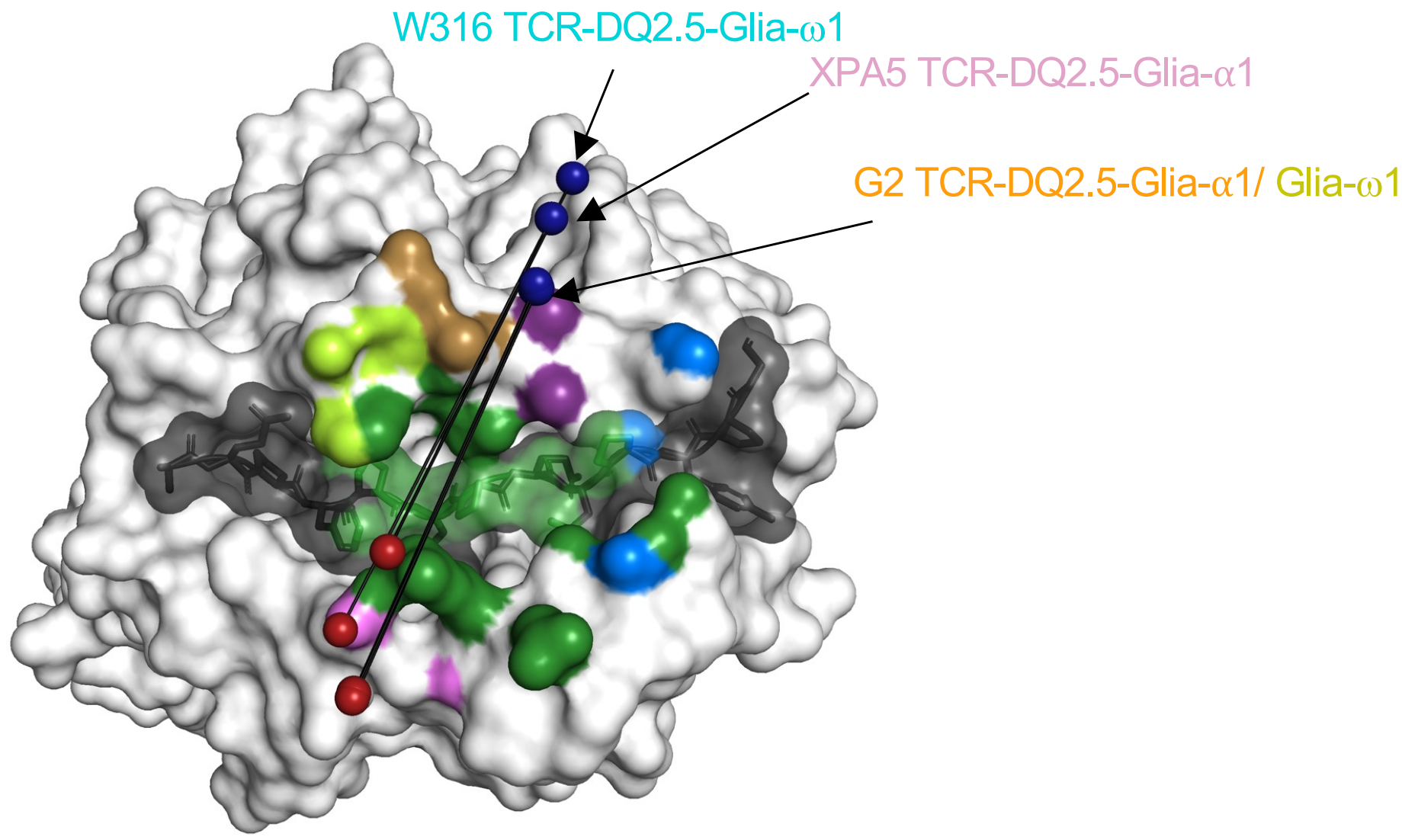

Fig. S8. Structural comparison of DQ2.5-glia-α1a/DQ2.5-glia-ω1 recognition by G2 and related TCRs

Comparison of TCR docking on pHLA from the ^{112}WD^{113}-encoded G2 TCR complexes, D^{112}-encoded XPA5 complex and D^{113}-encoded W316 TCR complex. TCR footprint of G2-TCR-DQ2.5-glia-α1a complex and overlaid centre of mass Vα and Vβ for XPA5, W316 and G2 complexes. The centre of mass for Vα and Vβ position is indicated by red and blue spheres, respectively, connected via a black line. The HLA-DQ2.5 α- and β-chains are shown in white, while the glia-α1a peptide is depicted in gray. The CDR loops and FWβ residues are coloured in accordance with legend for Fig. 5B.

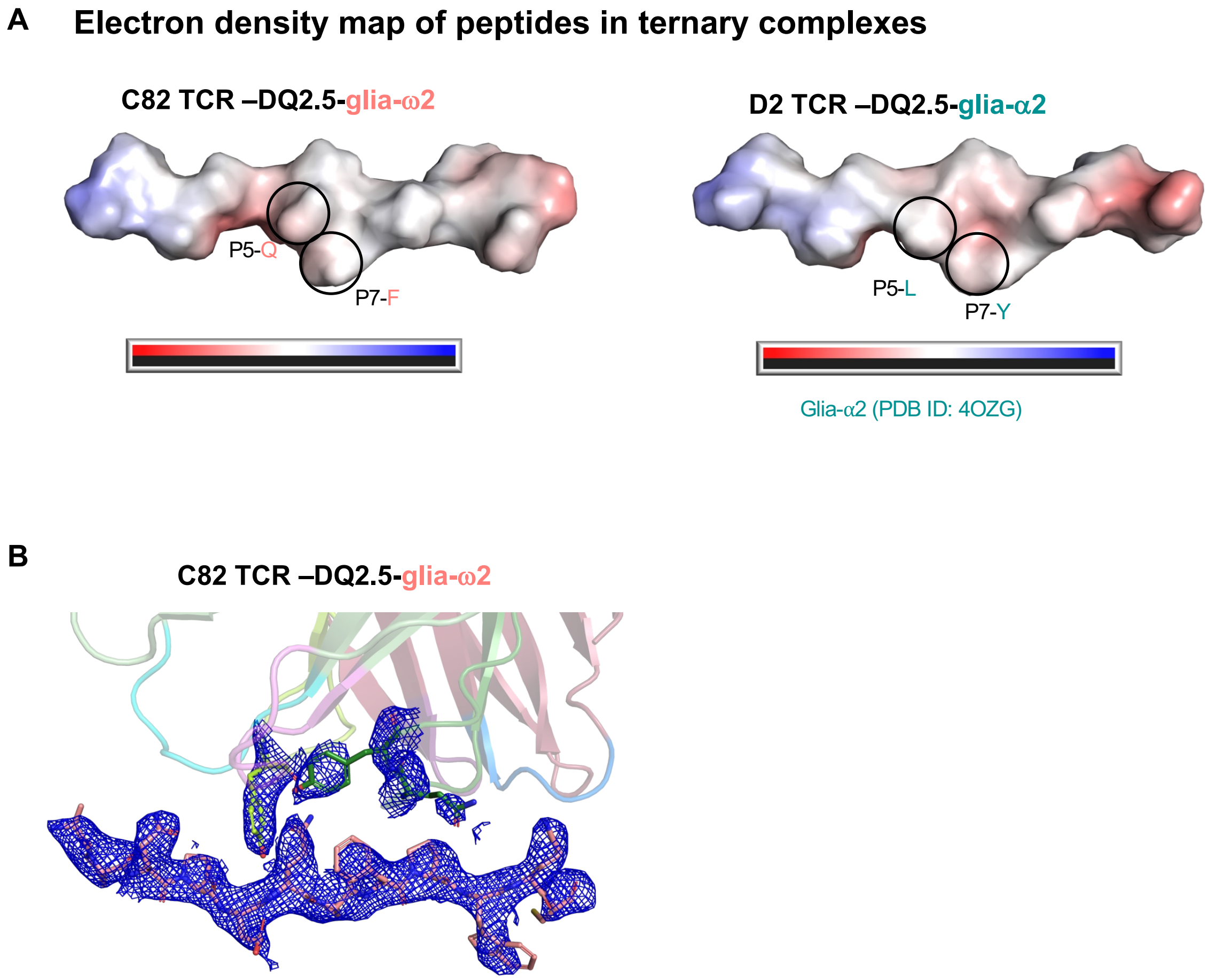

Fig. S9. Adaptive Poisson Boltzmann Solver-generated electrostatic surface of peptide (red, negative; blue, positive; white, neutral) and composite SA-omit map of the C82 TCR-DQ2.5-glia-ω2 complex.

(A) The view from the top of the peptide binding groove of DQ2.5-glia-α2-D2 TCR complex and DQ2.5-glia-ω2-C82 TCR complex. Black circle is the position where P5 and P7 residues are located. **(B)** The σA-weighted 2Fo-Fc map (blue, contoured at 1.0σ) shows electron density for interface residues for C82 TCR-DQ2.5-glia-ω2 complex. The glia-ω2 peptide in pink sticks. CDR loops are highlighted as follows: CDR1α (cyan), CDR2α (violet), and CDR3α (light green); CDR1β (blue), CDR2β (purple), CDR3β (dark green).

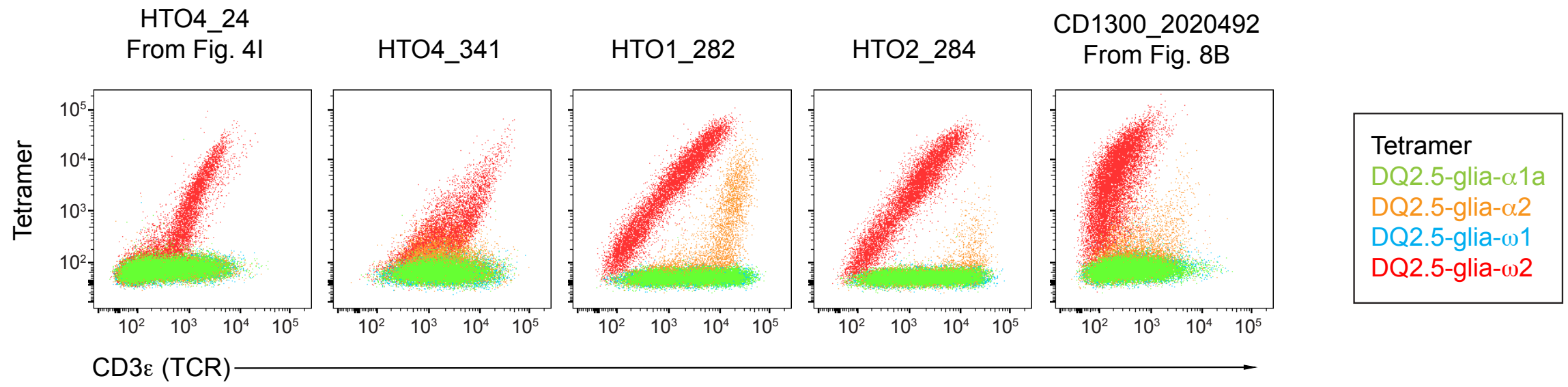

Clonotype ID	CDR3α	CDR3β	TRAV	TRAJ	TRBV	TRBJ
HTO4_24	CLVGDMRFTGGGNKLTF	CSAAGQHYGYTF	4	10	20-1	1-2
HTO4_341	CAVRAVTGGGYNKLIF	CASSATGQIHEQYF	1-2	4	2	2-7
HTO1_282	CVVNMYSSASKIIF	CASSTLQGHEQFF	12-1	3	28	2-1
HTO2_284	CVVNSPSSASKIIF	CASSLLQKHEQYF	12-1	3	28	2-1
CD1300_2020492	CVVNLGSSASKIIF	CASSPLQGHTQYF	12-1	3	28	2-3

Fig. S10. *TRAV12-TRAJ3-TRBV28* gene elements in conjunction with Glia-ω2 CDR3β Q.[H/Y] motif contribute to cross-reactivity with DQ2.5-glia-α2.

293T cells transiently co-transfected with CD3γδεζ and selected TCR from clonotypes using the CDR3β Q.[H/Y] motif predicting specificity for DQ2.5-glia-ω2 (Fig. 4D) were stained with either HLA-DQ2.5-glia-α1a tetramer (green), HLA-DQ2.5-glia-α2 tetramer (orange), HLA-DQ2.5-glia-ω1 tetramer (blue) or HLA-DQ2.5-glia-ω2 tetramer (red). Two of the selected TCR shown include clonotypes HTO4_24 and CD1300_2020492, previously shown in Figs. 4I and 8B, respectively. The CDR3α and CDR3β sequence and TRAV, TRAJ, TRBV and TRBJ usage of each TCR is indicated in the table below, with residues associated with the CDR3β motif in red type. TCR using *TRAV12-TRAJ3-TRBV28* gene elements in conjunction with the CDR3β motif were found to be cross-reactive with DQ2.5-glia-α2.

Table S1. CeD patient information

Subject[1]	HLA-DQ status	Sex	Age	Time since diagnosis [2]	tTG IgA serology [3]	DGP-IgG serology [3]	Co-morbidities	IFNγ ELISPOT to alpha and omega gliadin post wheat challenge (raw spot-forming units per million PBMC) [4]
CD0572 (HTO1)	DQ2.5	F	53	4 years	18	15	Nil	190
CD0648 (HTO2)	DQ2.5/DQ2.5	F	54	7 years	8	4	Nil	16
CD1314 (HTO3)	DQ2.5	M	68	7 years	ND	ND	Nil	56
CD1621 (HTO4)	DQ2.5/DQ2.5	F	68	10 years	4	9	Hashimoto's thyroiditis	292
CD1597	DQ2.5	F	25	2 years	8	11	Nil	40

[1] Post wheat challenge PBMC from donors CD0572, CD0648, CD1314 and CD1621 were used in scRNA seq; Post wheat challenge PBMC from donor CD0572 and CD1597 were used for TCR analysis following tetramer-based single cell flow cytometric cell sorting.
[2] Patients begin a gluten-free diet on diagnosis
[3] Serology performed on serum collected from PBMC donors prior to wheat challenge (day 0). Normal reference range: anti-transglutaminase 2 IgA antibodies (Anti-TG2) 0-20U/mL, anti-deamidated gliadin peptide IgG antibodies (ant-DGP) < 20 Units/mL.
[4] IFN-γ ELISpot (Mabtech) assays were performed and analyzed on matched PBMC collected at day 6 post wheat challenge, as previously described (*32*).

Table S2. Patient symptoms post wheat challenge

Donor CD0572 (HTO1; DQ2.5+): Scale: 0 None; 1 Minor; 2 Mild; 3 Moderate; 4 Moderate to severe; 5 Severe; 6 Very severe. † not included in assessment.

	Abdom-inal pain	Bloating	Consti-pation†	Diarrhoea †	Gas	Lethargy	Nausea	Vomiting
Baseline								
Day 1								
Day 2								
Day 3		2						
Day 4		3			1			
Day 5		4			1			
Day 6		3			1			

Donor CD0648 (HTO2; DQ2.5/DQ2.5): Scale: 0 None; 1 Minor; 2 Mild; 3 Moderate; 4 Moderate to severe; 5 Severe; 6 Very severe. † not included in assessment.

	Abdom-inal pain	Bloating	Consti-pation†	Diarrhoea †	Gas	Lethargy	Nausea	Vomiting
Baseline								
Day 1								
Day 2					1			
Day 3					1			
Day 4					1			
Day 5					1			
Day 6					1			

Donor CD1314 (HTO3; DQ2.5+): Scale 0-10: Nil to Worst possible

	Abdom-inal pain	Bloating	Consti-pation	Diarrhoea	Gas	Lethargy	Nausea	Vomiting
Baseline								
Day 1	3	5	1		5	1		
Day 2	3	5	1		5	1	5	
Day 3	1	1			2			
Day 4		1			2			
Day 5								
Day 6					1			

Donor CD1621 (HTO4; DQ2.5/DQ2.5): Scale 0-10: Nil to Worst possible

	Abdom-inal pain	Bloating	Consti-pation	Diarrhoea	Gas	Lethargy	Nausea	Vomiting
Baseline								
Day 1				3			5	5
Day 2				3			5	5
Day 3							1	
Day 4								
Day 5								
Day 6								

Table S3. TCR repertoires subjected to TCRdist analysis

a) Summary of unique paired[1] TCR sequences in TCRdist analysis

**Epitope specifity[2]**	**DQ2.5-glia-α1a**		**DQ2.5-glia-α2**		**DQ2.5-glia-ω1**		**DQ2.5-glia-ω2**		**DQ2.5-glia-α1a/ω1**		**TOTAL**	
**Donor**	**Cells**	**Clones[3]**	**Cells**	**Clones**	**Cells**	**Clones**	**Cells**	**Clones**	**Cells**	**Clones**	**Cells**	**Clones**
**HTO1**	174	62	268	102	90	26	179	50	36	15	746	252
**HTO2**	27	14	56	17	1	1	45	12	3	2	132	46
**HTO3**	60	25	77	25	55	7	8	8	13	9	213	74
**HTO4**	326	40	495	71	243	21	545	40	247	14	1860	186
**Total**	**587**	**141**	**896**	**215**	**389**	**55**	**777**	**110**	**299**	**40**	**2951**	**558**

b) TCR repertoires subjected to TCRdist analysis

**Epitope-specific repertoires**	**No. of subjects**	**No. of parsed reads**	**No. of clones[3]**	**Clonality[4]**	**Pshare[5]-α**	**Pshare-β**	**Pshare-αβ**
Glia-α1a	4	587	141	0.909	3.225E-03	2.471E-03	1.021E-03
Glia-α1a/ω1	4	299	40	0.717	0.000E+00	0.000E+00	0.000E+00
Glia-α2	4	896	215	0.945	3.784E-03	1.314E-02	1.885E-03
Glia-ω1	4	389	55	0.845	0.000E+00	0.000E+00	0.000E+00
Glia-ω2	4	777	110	0.915	5.028E-03	1.354E-03	1.354E-03

[1]TCRs with a single paired alpha and beta chain. TCR with dual alpha or beta chains were excluded. [2]Epitope specificity is based on dextramer binding. [3]Two TCRs are considered as belonging to the same clone if they are from the same individual and have identical nucleotide sequences. [4]Clonality is measured by first computing 1.0-Simpson's diversity index of the clone size distribution for each subject and then averaging these values over the different subjects with weights based on the size of each subject's repertoire. [5]Pshare is the estimated rate at which a clone drawn from one subject has an identical amino acid sequence to one drawn from another subject.

Table S4. CDR3 loop sequence and gene usage of motif-bearing TCR selected for expression in cell lines.

Figure	Clonotype ID[1]	# sequences	CDR3α	CDR3β	TRAV	TRAJ	TRBV	TRBJ
4I	HTO1_92	8	CIETSGSRLTF	CASSIRSTDTQYF	26-1	58	7-2	2-3
4I	HTO4_11	52	CAESSPGKLIF	CASSLAQPSTDTQYF	5	37	5-1	2-3
4I	HTO4_2	98	CAVRSVFSGGYNKLIF	CASSEIAGGNEQFF	1-2	4	2	2-1
4I	HTO4_24	28	CLVGDMRFTGGGNKLTF	CSAAGQHYGYTF	4	10	20-1	1-2
4I	HTO4_61	13	CAGEKGDSWGKLQF	CSGGQGGTGELFF	27	24	29-1	2-2
4I	HTO1_643	1	CAGQLGGATNKLIF	CASSLVAWDTEAFF	35	32	5-1	1-1
4I	HTO4_161	4	CLVGEPISGGYNKLIF	CASSIAWSTEAFF	4	4	19	1-1
4I	HTO3_685	1	CAMDNAGGTSYGKLTF	CASSSGSEGSPLHF	12-3	52	4-3	1-6
4J	HTO3_275	2	CAVQTGANNLFF	CAWQRTGWDTEAFF	12-2	36	30	1-1
4J	HTO3_261	2	CAENVSGGSNYKLTF	CSVGAWEGTQYF	13-2	53	29-1	2-5
4J	HTO2_200	2	CLVGDENTGTASKLTF	CAIRGWDSPLHF	4	44	6-2	1-6
4J	0572_G2	1	CAGQYGGATNKLIF	CASSLVAWDTEAFF	35	32	5-1	1-1
4J	1597_C8	1	CAASQTGANNLFF	CASSLNWDTEAFF	29/DV5	36	7-8	1-1
4J	HTO4_81	9	CAVGVTEYGNKLVF	CAWSAQGWDTGELFF	8-3	47	30	2-2
4J	HTO4_29	25	CAVQATRGGGADGLTF	CASSIGWSGKLYF	20	45	19	1-4
4J	0572_D9	3	CALSDQGRGSTLGRLYF	CASSIAWTGEQYF	9-2	18	19	2-7
4J	0572_H9	2	CALSEMGRGSTLGRLYF	CASSIGWTDTQYF	9-2	18	19	2-3
4J	HTO1_99	7	CAGQLNAGGTSYGKLTF	CASGGAWGNEQFF	35	52	19	2-1
4J	HTO1_113	6	CAGTNYGGSQGNLIF	CASSIGWVAEQFF	35	42	19	2-1
7	HTO1_82 (C82 TCR)	9	CAMRGYNFNKFYF	CASSLGGQYNEQFF	14/DV4	21	5-4	2-1
8B	CD1375_2019172	1	CAEPGGARLMF	CASSLGQPSTDTQYF	5	31	5-1	2-3
8B	CD114_2023369	1	CAVRAVLSGGYNKLIF	CASSPTGEQFF	1-2	4	2	2-1
8B	CD1300_2020492	18	CVVNLGSSASKIIF	CASSPLQGHTQYF	12-1	3	28	2-3
8B	CD1339_2019970	16	CAVEGGSNYKLTF	CSVGAGGTGELFF	41	53	29-1	2-2
8B	CD1375_2019107	6	CAENDARAGGGADGLTF	CASSIGWVNEQFF	13-2	45	19	2-1
8B	CD1340_2019843	4	CAVGLAEYGNKLVF	CAWSVRGWDTGELFF	8-3	47	30	2-2
8B	CD412_2018680	1	CAGDSGGGADGLTF	CASSGGPRTVYSGNTIYF	35	45	5-1	1-3
S10	HTO4_341	2	CAVRAVTGGGYNKLIF	CASSATGQIHEQYF	1-2	4	2	2-7
S10	HTO1_282	2	CVVNMYSSASKIIF	CASSTLQGHEQFF	12-1	3	28	2-1
S10	HTO2_284	2	CVVNSPSSASKIIF	CASSLLQKHEQYF	12-1	3	28	2-7

[1]Clonotype ID also indicates the donor from which the TCR was isolated. Clonotypes beginning with "HTO1-4" or "0572/1597" are $CD4^+$ T cells sorted from PBMC from wheat challenged CeD donors after staining with barcoded dextramers to HLA-DQ2.5-gliadin-α1a, -α2, -ω1 and -ω2 (HTO1-4) or with individual tetramers to HLA-DQ2.5-gliadin-α1a or -ω1 (0572 & 1597). Clonotypes beginning with "CD" are $CD4^+$ T cells isolated from CeD patient PBMC and/or duodenum with a pooled cocktail of DQ2.5-glia-α1a, -α2, -ω1 and -ω2 tetramers (*30*).

Table S5. Range of dextramer UMI counts per cell for clonotypes expressing the DQ2.5-Glia-α1a-associated CDR3β W[D/E]….F or DQ2.5-Glia-ω1-associated CDR3β W…..F motif

The DQ2.5-Glia-α1a- and DQ2.5-Glia-ω1-associated CDR3β motifs are described in Figures 4F and 4G, respectively. Residues associated with these CDR3β motifs are shown in red type below.

Clono-type ID	HTO ID	# cells per clono type	CDR3α	CDR3β	TRAV	TRAJ	TRBV	TRBJ	Range of dextramer UMI counts per cell for each clonotype			
									DQ2.5 -Glia-α1a	DQ2.5 -Glia-α2	DQ2.5 -Glia-ω1	DQ2.5-Glia-ω2
81	HTO4	9	CAVGVTEYGNKLVF	CAWSAQGWDTGELFF	8-3	47	30	2-2	23-242	0-1	0-3	0-1
93	HTO1	7	CAGQLGGFGNVLHC	CASSLVAWDTEAFF	35	35	5-1	1-1	178-225	0-4	0-11	0-2
163	HTO3	4	CAVGAAEYGNKLVF	CAWSATGWDTGELFF	8-3	47	30	2-2	235-407	0	0-2	0-1
200	HTO2	3	CLVGDENTGTASKLTF	CAIRGWDSPLHF	4	44	6-2	1-6	13-77	0-1	0-2	0
201a	HTO1	1	CAVSPLEYGNKLVF	CSASRTAWDTEAFF	8-6	47	20-1	1-1	171	0	1	0
201b	HTO1	2	CAVSPLEYGNKLVF	CSASRTAWDTEAFF	8-6	47	20-1	1-1	45, 159	0	0, 4	0, 1
224	HTO4	3	CAGQGDSGSRLTF	CASSLVFWDSEAFF	35	58	5-5	1-1	44-82	0-1	0	0
260	HTO1	2	CAVGVSGSARQLTF	CSVADWDGELFF	12-2	22	29-1	2-2	31, 101	3, 0	0	0, 1
261	HTO3	2	CAENVSGGSNYKLTF	CSVGAWEGTQYF	13-2	53	29-1	2-5	199, 299	0, 3	364, 429	0
275	HTO3	2	CAVQTGANNLFF	CAWQRTGWDTEAFF	12-2	36	30	1-1	115, 186	1	50, 65	0
342	HTO4	2	CAGEDPTGFQKLVF	CASSATGWDTEAFF	35	8	7-3	1-1	196, 234	0, 1	0, 4	1, 0
394	HTO1	1	CICEDNNARLMF	CSARITWDTEAFF	26-1	31	20-1	1-1	292	1	0	0
396	HTO4	1	CAGLQGAQKLVF	CSVVMDWDQPQHF	35	54	29-1	1-5	81	2	0	0
423	HTO2	1	CAYSAGMEYGNKLVF	CSARVVWDQPQHF	38-2	47	20-1	1-5	440	0	0	0
643	HTO1	1	CAGQLGGATNKLIF	CASSLVAWDTEAFF	35	32	5-1	1-1	135	1	1	0
644	HTO1	1	CAGQPGGATNKLIF	CASSLVQWDTEAFF	35	32	5-1	1-1	30	0	0	1
648	HTO1	1	CAVVGNNNARLMF	CASSLVWEDEQFF	2	31	7-2	2-1	262	0	3	0
657	HTO1	1	CAPIGTSGSRLTF	CASSLVAWDTEAFF	36DV7	58	5-1	1-1	108	0	1	0
690	HTO3	1	CAASAGGATNKLIF	CASSLTWDTEAFF	29DV5	32	7-8	1-1	202	0	2	0
722	HTO1	1	CAGDENTGFQKLVF	CASSLTGWDTEAFF	35	8	7-8	1-1	39	0	3	0
29	HTO4	22	CAVQATRGGGADGLTF	CASSIGWSGKLFF	20	45	19	1-4	0-17	0-9	0-106	0-2

99	HTO1	6	CAGQLNAGGTSYGKLTF	CASGGAWGNEQFF	35	52	19	2-1	0-2	0-2	63-296	0-2
113	HTO1	6	CAGTNYGGSQGNLIF	CASSIGWVAEQFF	35	42	19	2-1	0-1	0-3	53-252	0-1
161	HTO4	4	CLVGEPISGGYNKLIF	CASSIAWSTEAFF	4	4	19	1-1	0-1	1-2	164-394	0
206	HTO4	3	CAGQLAAGGTSYGKLTF	CASGTSWGSEQFF	35	52	19	2-1	0	0-2	36-97	0-1
322	HTO1	2	CATVDSSYKLIF	CASSIIWGDTEAFF	17	12	19	1-1	0	1, 4	143, 292	0
500	HTO1	1	CANFAGGSNYKLTF	CASSIAWTGELFF	12-3	53	19	2-2	0	1	62	0
516	HTO3	1	CAAPAGGYQKVTF	CASSIAWTAGLFF	23DV6	13	19	2-2	1	3	315	0
519	HTO1	1	CAASSPSASNTGKLIF	CASSIGWSYGYTF	29DV5	37	19	1-2	2	0	85	0
640	HTO3	1	CARVDSSYKLIF	CASSIVWGDTEAFF	17	12	19	1-1	0	0	149	2

Table S6. Data collection and refinement statistics of G2 TCR- and C82 TCR-DQ2.5 complexes

	G2 TCR-DQ2.5-glia-α1a	G2 TCR-DQ2.5-glia-ω1	C82 TCR-DQ2.5-glia-ω2
**Data collection**			
Space group	P212121	P212121	P22121
Cell dimensions			
a, b, c (Å)	90.62, 122.67, 252.86	88.99, 122.21, 249.57	106.92, 144.01, 147.94
α, β, γ (°)	90, 90, 90	90, 90, 90	90, 90, 90
Resolution (Å)[a]	49.59-3.25 (3.36-3.25)	49.25-3.2 (3.31-3.2)	46.65-3.25 (3.39-3.25)
R_{sym} or R_{merge} [a]	0.29 (1.18)	0.25 (0.80)	0.19 (1.21)
I /σ (I)[a]	6.5 (1.9)	5.7 (2.4)	7.7 (1.8)
CC1/2	0.971 (0.639)	0.966 (0.750)	0.992 (0.551)
Completeness (%)[a]	99.9 (99.8)	100.0 (100.0)	99.9 (100.0)
Redundancy [a]	9.2 (9.5)	6.8 (7.1)	6.8 (6.8)
**Refinement**			
Resolution (Å)	49.59-3.25	49.25-3.20	46.65-3.25
No. reflections	45120	45735	36619
R_{work} / R_{free}	0.224/0.257	0.187/0.225	0.221/0.267
No. atoms	12891	13048	12810
Protein	12702	12818	12640
Ligand/ion	156	190	138
Water	33	40	32
B-factors (Å^2)	62.47	54.39	85.75
Protein	62.22	53.99	85.42
Ligand/ion	88.88	85.61	122.99
Water	34.80	32.46	56.46
R.m.s. deviations			
Bond lengths (Å)	0.004	0.003	0.003
Bond angles (°)	0.710	0.568	0.596

[a] Values in parentheses refer to the highest resolution shell

Table S7. TCR-pMHC II complex statistics

**TCR-pMHC**	**BSA** ($Å^2$)	**Vα** (%)	**Vβ** (%)	**Peptide** (%)	**1α** (%)	**2α** (%)	**3α** (%)	**Fα** (%)	**1β** (%)	**2β** (%)	**3β** (%)	**Fβ** (%)
G2 TCR-DQ2.5-glia-α1a	2024	25.4	74.6	19.7	2.9	11.0	10.1	1.4	10.7	5.9	51.4	6.6
G2 TCR-DQ2.5-glia-ω1	2031	27.5	72.5	19.8	2.3	14.3	9.3	1.5	11.0	6.3	49.3	6.0
C82 TCR-DQ2.5-glia-ω2	1960	54.0	46.0	23.9	8.3	15.5	28.1	2.1	4.3	14.0	26.9	0.8

Table S8. Contact table of *TRAV35*$^{+}$*TRBV5-1*$^{+}$ G2 TCR-DQ2.5-glia-α1a

TCR segment	TCR residues	HLA-DQ2.5/α1a	Type of bond
**CDR1α**	T38	R77β	HB
**CDR2α**	Y57 (OH)(O)	D66β, E69β, R70β (NE), R77β (NH1)	VDW, HB
**CDR3α**	G110	D57α, F60α,	VDW
	A111 (N)	D57α (O), Q59α, F60α,	VDW
**CDR1β**	S27 (O)	Y60β (OH)	HB, VDW
	G28	Y60β	
	H29 (NE2)	Y60β, Q64β (OE1)	HB, VDW
	R37 (NH2)	H70α (NE2),	VDW, HB
**CDR2β**	F57	T63α, V67α	VDW
	S58	A66α	VDW
**FWβ**	**R66**(NH2)	Q59α , T63α	HB, VDW
**CDR3β**	L108	Y60β, Q64β, I67β	VDW
	A110	T63α	VDW
	W112 (NE1)	F60α, T63α (O), N64α	VDW, HB
	D113 (O)(OD1)	R70β (NE), R77β (NH2)	VDW, SB
	T114 (O)	R70β (NH1)	HB, VDW
	E115 (OE1)	D66β, R70β (NH1)	VDW, SB
	F117	D66β	VDW
**TCR segment**	**TCR residues**	**glia-α1a peptide**	**Type of bond**
**CDR1β**	G28	P8-P	VDW
	R37	P8-P	VDW
**CDR3β**	L108	P8-P	VDW
	V109	P5-P, P6-E, P7-L, P8-P	VDW
	W112	P3-P, P4-Q, P5-P	VDW
	D113	P2-F, P5-P	VDW

VDW: Van der Waals interaction (cut-off at 4 Å)
HB: hydrogen bond (cut-off at 3.5 Å)
SB: salt bridge (cut-off at 4.5 Å)

Table S9. Contact table of *TRAV35*$^{+}$*TRBV5-1*$^{+}$ G2 TCR-DQ2.5-glia-ω1

TCR segment	TCR residues	HLA-DQ2.5/ω1	Type of bond
**CDR1α**	N37	R77β	VDW
	T38 (OG1)	R70β, R77β (NH2)	HB, VDW
**CDR2α**	Y57 (OH)(O)	D66β, E69β, R70β, A73β, R77β (NE)	VDW, HB
	K58	D76β	VDW
**CDR3α**	Q107 (OE1)	R70β (NH1, NH2)	HB, VDW
	G109	F60α	VDW
	G110	F60α	VDW
	A111 (N)	D57α (OD2), Q59α, F60α,	HB, VDW
**CDR1β**	S27 (O)	Y60β (OH)	HB, VDW
	G28	Y60β	VDW
	H29 (NE2)	Q64β (NE2)	HB, VDW
	R37	H70α	VDW
**CDR2β**	F57	T63α, A66α, V67α	VDW
	S58	A66α	VDW
**FWβ**	R66(NH1)(NE)	Q59α (OE1)	HB, VDW
**CDR3β**	L108	Y60β, Q64β, I67β	VDW
	V109	V67α	VDW
	A110	T63α	VDW
	W112 (NE1)	F60α, T63α (OG1), N64α	HB, VDW
	D113 (O)(OD1)	R70β (NE), R77β (NH1, NH2)	VDW, SB
	T114 (O)	R70β (NH1, NH2)	HB, VDW
	E115 (OE2)(N)	D66β (O), R70β (NE)(NH1)	HB, VDW, SB
	F117	D66β	VDW
**TCR segment**	**TCR residues**	**glia-ω1 peptide**	**Type of bond**
**CDR1β**	G28	P8-P	VDW
	R37	P8-P	VDW
**CDR3β**	L108	P7-Q, P8-P	VDW
	V109	P6-E	VDW
	W112	P3-P, P4-Q, P5-P	VDW
	D113	P2-F	VDW

VDW: Van der Waals interaction (cut-off at 4 Å)
HB: hydrogen bond (cut-off at 3.5 Å)
SB: salt bridge (cut-off at 4.5 Å)

Table S10. Invariant chain and gluten epitopes sequences

Peptides	Amino acid sequence	Sequence identity
DQ2.5-glia-α1a	QPFPQPELPYP	100%
DQ2.5-glia-ω1	QPFPQPEQPFP	82%
DQ2.5-glia-α2	APQPELPYPQP	73%
DQ2.5-glia-ω2	APQPEQPFPWQ	55%
DQ2.5-glia-γ2	QGIIQPEQPAQLIR	45%
DQ2.5-hordein 3	QPIPEQPQPY	50%

Underlined sequences denote peptide-binding register to HLA-DQ from P1-P9

Sequence identity was aligned using the DQ2.5-glia-α1a peptide sequence as reference

Table S11. Contact table of *TRAV14*+*TRBV5-4*+C82 TCR-DQ2.5-glia-ω2

TCR segment	TCR residues	HLA-DQ2.5/ω2	Type of bond
**CDR1α**	D29	F57α	VDW
	P30	R113β	VDW
	S36 (O)	R113β (NE)	HB, VDW
	Y37 (O)	R106β (NH2)	HB, VDW
**FWα**	Y55 (OH)	D102β (O), R106β (NH2)	HB, VDW
**CDR2α**	G57	D102β, R106β	VDW
	S58 (O)(OG)	R106β (NE, NH2)	HB, VDW
	Y59 (O)	E105β (OE1), R106β, A109β, R113β	HB, VDW
	D62	E105β	VDW
**CDR3α**	Y109 (OH)	F57α, T60α, N61α (OD1, ND2)	HB, VDW
	N110 (OD1)	Q56α(O), F57α, T60α (OG1)	HB, VDW
	F113	T60α, A63α	VDW
	N114 (OD1,ND2)	Q56α (NE2), T60α (OG1)	HB, VDW
**CDR1β**	N37	H67α	VDW
**CDR2β**	Y57 (OH)	A63α (O), V64α, H67α	HB, VDW
	R58 (NH2)	H67α (ND1), N70α	HB, VDW
**CDR3β**	L108	Y96β	VDW
	G109	V64α	VDW
	G110	V64α	VDW
	Q112 (OE1,NE2)	Y96β(OH), Q100β (NE2)	HB, VDW
	Y113	D102β, R106β	VDW
	N114	Q100β	VDW
**TCR segment**	**TCR residues**	**glia-ω2 peptide**	**Type of bond**
**CDR1α**	D29 (OD1)	P2-Q (NE2)	HB, VDW
**CDR3α**	Y109 (OH,O)	P3-P, P4-E (O), P5-Q (OE1), P6-P	HB, VDW
**CDR1β**	G28 (O)	P10-Q (OE1, NE2)	HB, VDW
**CDR3β**	L108	P8-P, P10-Q	VDW
	G109	P8-P	VDW
	G110	P7-F, P8-P	VDW
	Q112	P8-P	VDW
	Y113 (OH)	P5-Q (OE1), P7-F	HB, VDW

VDW: Van der Waals interaction (cut-off at 4 Å)
HB: hydrogen bond (cut-off at 3.5 Å)
SB: salt bridge (cut-off at 4.5 Å)

Table S12. Antibodies

Antibody name [clone]	Source	Catalog number
AF700 mouse anti-human CD14 [M5E2]	BD Biosciences	557923
AF700 mouse anti-human CD19 [HIB19]	BD Biosciences	557921
BV480 mouse anti-human CD3 [UCHT1]	BD Biosciences	566105
BUV395 mouse anti-human CD4 [SK3]	BD Biosciences	563550
Brilliant Stain buffer plus	BD Biosciences	566385
TotalSeq™-C0081 anti-human CD14 [M5E2]	Biolegend	301859
TotalSeq™-C0140 anti-human CD183 (CXCR3)	Biolegend	353747
TotalSeq™-C0144 anti-human CD185 (CXCR5)	Biolegend	356939
TotalSeq™-C0071 anti-human CD194 (CCR4)	Biolegend	359425
TotalSeq™-C0143 anti-human CD196 (CCR6)	Biolegend	353440
TotalSeq™-C0148 anti-human CD197 (CCR7)	Biolegend	353251
TotalSeq™-C0085 anti-human CD25	Biolegend	302649
TotalSeq™-C0154 anti-human CD27	Biolegend	302853
TotalSeq™-C0171 anti-human/mouse/rat CD278 (ICOS)	Biolegend	313553
TotalSeq™-C0088 anti-human CD279 (PD-1)	Biolegend	329963
TotalSeq™-C0169 anti-human CD366 (Tim-3)	Biolegend	345049
TotalSeq™-C0389 anti-human CD38	Biolegend	303543
TotalSeq™-C0072 anti-human CD4 [RPA-T4]	Biolegend	300567
TotalSeq™-C0063 anti-human CD45RA	Biolegend	304163
TotalSeq™-C0087 anti-human CD45RO	Biolegend	304259
TotalSeq™-C0159 anti-human HLA-DR	Biolegend	307663
TotalSeq™-C0089 anti-human TIGIT (VSTM3)	Biolegend	372729
TotalSeq™-C0156 anti-human CD95 (Fas)	Biolegend	305651
TotalSeq™-C0090 mouse IgG1 κ isotype control	Biolegend	400187
TotalSeq™-C0091 mouse IgG2a κ isotype control	Biolegend	400293
TotalSeq™-C0251 anti-human Hashtag 1	Biolegend	394661
TotalSeq™-C0252 anti-human Hashtag 2	Biolegend	394663
TotalSeq™-C0253 anti-human Hashtag 3	Biolegend	394665
TotalSeq™-C0254 anti-human Hashtag 4	Biolegend	394667
